# Causes of death after biannual azithromycin treatment: A community-level randomized clinical trial

**DOI:** 10.1371/journal.pone.0250197

**Published:** 2021-09-24

**Authors:** Evan M. Bloch, Zakayo Mrango, Jerusha Weaver, Beatriz Munoz, Thomas M. Lietman, Sheila K. West

**Affiliations:** 1 Department of pathology, Johns Hopkins School of Medicine, Baltimore, MD, United States of America; 2 National Institute for Medical Research, Kilosa, Tanzania; 3 Dana Center for Preventive Ophthalmology, Johns Hopkins School of Medicine, Baltimore, MD, United States of America; 4 Francis I Proctor Foundation, University of California, San Francisco, San Francisco, CA, United States of America; PLOS, UNITED KINGDOM

## Abstract

The MORDOR study, a masked, community-level randomized clinical trial conducted in Niger, Malawi and Tanzania (2015 to 2017), showed that biannual administration of single-dose azithromycin to preschool children reduced all-cause mortality. We sought to evaluate its impact on causes of death in children aged 1–59 months in Tanzania. A random sampling of 614 communities was conducted in Kilosa District, Tanzania, with simple random assignment of communities to receive either azithromycin or placebo. In these communities, a census was carried out every 6 months and children aged 1–59 months received biannual (every 6 months), single-dose azithromycin (~20mg/kg) or placebo depending on community assignment, over a 2-year period. Mortality was determined at the time of the biannual census. For child deaths, a verbal autopsy was performed to ascertain the cause using a standardized diagnostic classification. A total of 190- (0.58 /100 person-years) and 200 deaths (0.59/100 person-years) were reported in the azithromycin and placebo arms, respectively. Malaria, pneumonia and diarrhea, accounted for 71% and 68% of deaths in the respective arms. Overall, the mortality was not different by treatment arm, nor were the distribution of causes of death after adjusting for community clustering. The cause-specific mortality for diarrhea/pneumonia was no different over time. In children aged 1–5 months, 32 deaths occurred in the placebo arm and 25 deaths occurred in the azithromycin arm; 20 (62.5%) deaths in the placebo- and 10 (40%) in the azithromycin arm were attributed to diarrhea or pneumonia. Neither differences in the number of deaths nor the diarrhea/pneumonia attribution was statistically significant after adjusting for community clustering. In conclusion, azithromycin was not associated with a significant decline in deaths by specific causes compared to placebo. The non-significant lower rates of diarrhea or pneumonia in children <6 months who received azithromycin merit further investigation in high-mortality settings.

**Trial registration:**NCT02048007.

## Introduction

Since 2000, there has been a 44% reduction in mortality in children aged under 5 years [1] which—in large part—is attributable to the successful prevention and/or treatment of pneumonia, measles, diarrhea and malaria [2, 3]. Nonetheless at time of transition to the Sustainable Development Goals (SDGs) in 2015 [4], less than a third of countries had achieved a two thirds reduction in child mortality as targeted by the original Millennium Development Goals [5]. Ninety percent of deaths in children under five still occur in low middle-income countries (LMICs) where pneumonia, diarrheal disease, and malaria remain the leading causes of death [2]. In 2017 alone, there were nearly 5.5 million deaths of children under 5, approximately half of which occurred in sub-Saharan Africa [6]. By 2030, childhood deaths (in those under age 5) are expected to increase proportionately in sub-Saharan Africa given reductions in deaths in other developing regions of the world [7]. Importantly, much of the mortality burden in LMICs is preventable or treatable.

In 2017, the MORDOR (Macrolides Oraux pour Réduire les Décès avec un Oeil sur la Résistance) trial of biannual single-dose azithromycin showed an aggregate reduction in all-cause mortality in pre-school children [8]. However, the benefit on mortality was shown to be significant only in Niger, not in Tanzania or Malawi. The benefit also appeared to be greatest for those children aged less than 6 months. The mechanism for a protective effect remains unclear. An antimicrobial effect seems the most plausible explanation given that azithromycin has a broad-spectrum effect, including action against an array of respiratory and gastrointestinal pathogens, which are leading causes of mortality in low-income countries [2]. Azithromycin has also some efficacy against malaria, which is a major mortality risk particularly in sub-Saharan Africa [9, 10]. The purpose of this analysis was to examine the effect of azithromycin compared to placebo on the specific causes of death in those subjects who were enrolled in the Tanzanian site of the MORDOR trial.

## Materials and methods

### Overview

A cluster-randomized, placebo-controlled, double-blinded clinical trial was conducted in 614 randomly selected communities in Kilosa District, Tanzania (January 2015 to August 2017) as part of the MORDOR trial, to evaluate the effect of biannual, single-dose azithromycin on mortality in children under age 5. The associated methods and results for all-cause mortality across all sites have previously been described [8]. We present a pre-specified sub-analysis to determine the effect of azithromycin on cause-specific mortality in children aged 1–59 months old in Tanzania. While the MORDOR study was conducted in Niger, Malawi and Tanzania. This sub-analysis is restricted to data from the Tanzanian study sites.

### Eligibility

**Fig 1:**
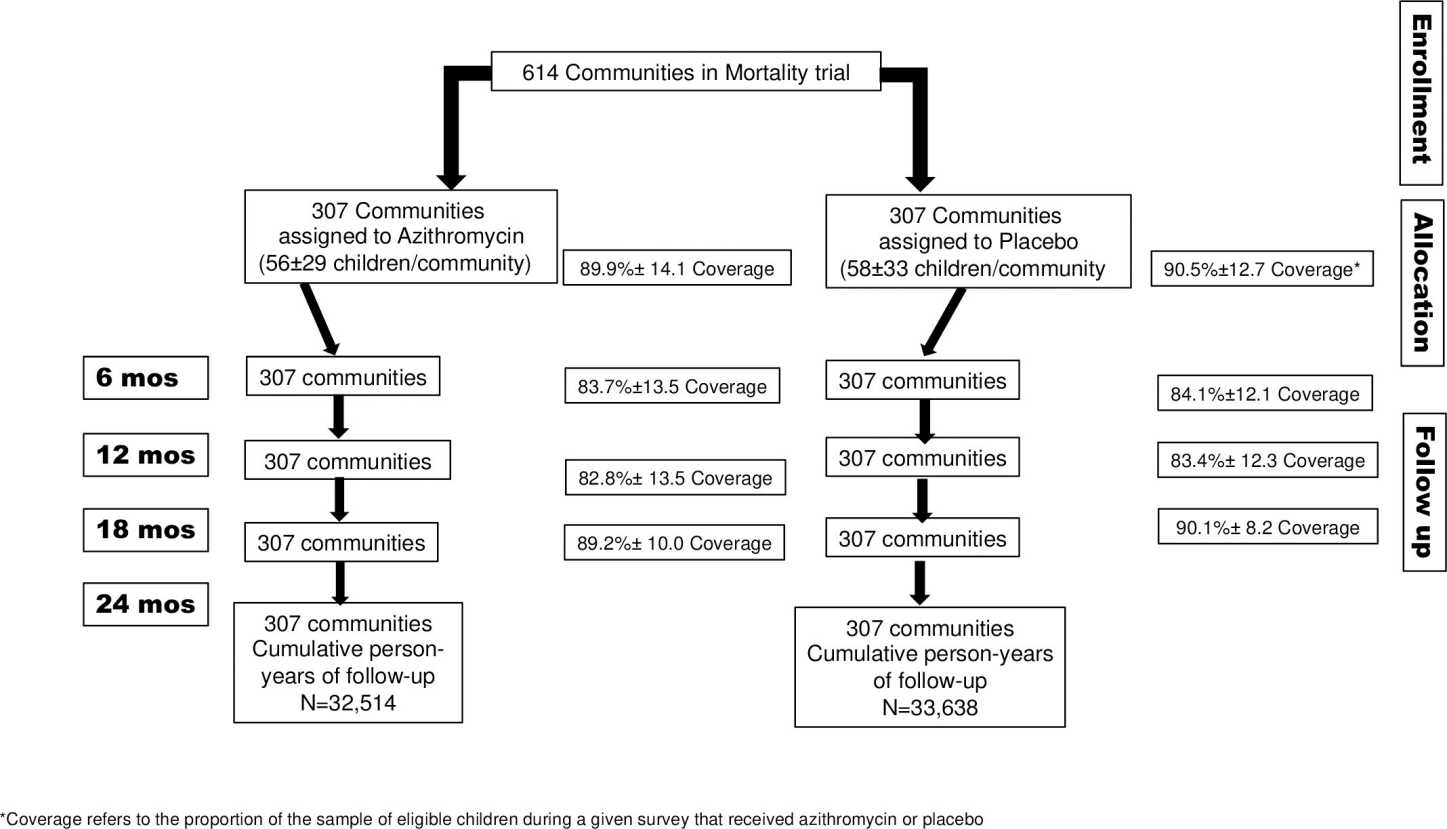
CONSORT diagram: Enrollment, randomization, treatment and follow up of communities in Tanzania.

All communities that were located in Kilosa district were eligible to participate in the trial (Fig 1). At each 6-monthly census, all households in the participating communities were eligible to participate if they had a child aged 1 to 59 months at the time of the census. Children who were younger than one month or who weighed less than 5 lbs were not eligible. At each census, children who newly aged into the study were added and those who aged out were disenrolled. In the communities, all children aged 1–59.9 months were offered oral azithromycin at 20mg/kg, provided in powder form every 6 months, or an identical placebo formulation. Children who were noted to have an adverse reaction to their study medication were excluded from future rounds of treatment. No adverse events were noted. The communities were a random sample of communities in Kilosa district and are considered representative of a larger rural population in sub Saharan Africa.

### Recruitment

The communities were initially approached through the village leadership and a series of community meetings to gain approval for participation. Once the community agreed, a date was set for the census and obtaining of individual consent from each family. Thus, the families already knew about the study in their community and individual consent was sought from the parents in each family at the time of census. New families were enrolled at each subsequent census if they had a child in the appropriate age range, and new children were enrolled if they met the age criteria.

*Randomization to treatment arm and masking*. The randomization sequence was generated by the unmasked statistician at University of California San Francisco, using a series of 8 letters with 1:1 allocation, and implemented by the Tanzanian study team. The 8 letters refer to assignment to study medication (4 each for azithromycin and control). The study statistician performed a simple randomization of communities without stratification into azithromycin and placebo arms. The trial was double-blinded such that the treatment assignment was unknown to the participants and study teams. The azithromycin and placebo powders, supplied in identical containers, were indistinguishable in appearance and taste.

### Intervention

The intervention was azithromycin (20mg/kg) or placebo (Pfizer, New York, NY, USA); The treatments were administered every six months for 24 months.

### Primary outcome

The distribution of causes of death in children aged 1-59months, where the cause of death was determined using a modified version of a published, computer-based algorithm [11].

### Verbal autopsies

A census of the participating communities was conducted at 6-monthly intervals. At each follow-up visit, those conducting the census would enquire specifically about new children aged 1 to 59 months in the household, and any deaths in children in the interval (i.e. since the time of the previous census). In the event of a reported death, a physician (ZM) trained in verbal autopsy methods would return to the household within one month of reporting to conduct a verbal autopsy [12, 13].The verbal autopsy was comprised of an interview with the deceased child’s primary guardian (e.g. mother) using the WHO abbreviated Verbal Autopsy questionnaire [14]. If the child’s guardian was unavailable, a relative or neighbor was interviewed. The interview was conducted in Swahili or the local language of the interviewee. Variables that were captured by the questionnaire included date of death, age of child at date of death, type and duration of symptoms.

### Classification of cause of death

**Fig 2:**
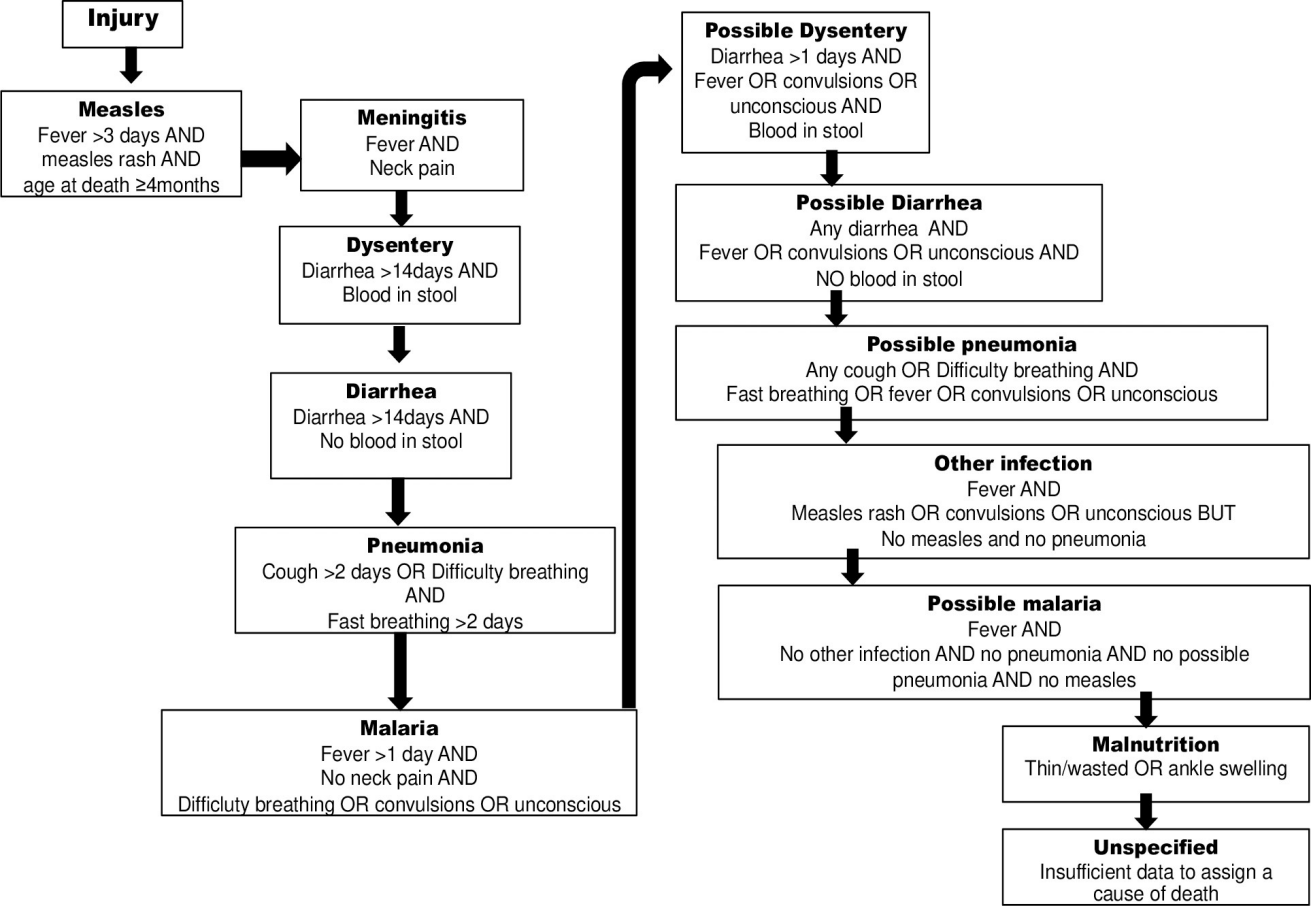
Causes of death as assigned using a modified verbal autopsy hierarchy [11].

The cause of death was classified into one of fourteen categories using a computer-based algorithm as previously described [8, 11] (Fig 2). All cases of malnutrition and “unspecified” were reviewed by the investigators (EB and SW) who were blinded to treatment assignment. Cases where the symptoms suggested one cause but the duration of symptoms before death was not long enough to categorize the outcome accordingly often defaulted to “malnutrition”; the investigators allowed these cases to be re-categorized as a possible alternative. Data are presented as originally categorized as well as according to re-categorization.

### Data management and statistical analyses

The number of deaths from baseline to the end of the study were divided by the number of person years of observation to determine the overall mortality rate. As such, each child contributed a certain number of months of observation depending on how long they had participated in the study. For example, if a subject participated in all phases of the study, they will have contributed 24-person months. In the event that a child moved during the study and returned at a later census update, their participation was not terminated at the time of the move and they were able to recover the months of observation. If a child was determined to be in a hospital or clinic (“not in the community”) at the time of the census update, and subsequently died at the next update, the child’s death was counted in the census period in which they died. Children who died during a census period were given half the person months of observation for that period.

The pre-specified primary analysis was conducted using a negative binomial regression of the number of deaths per community, with treatment group as predictor and total person-time at risk as an offset. Incidence rate ratios and 95% confidence intervals were derived from the model. Hypothesis testing was two-sided, with an overall alpha level of 0.05. Data were analyzed with SAS version 9.4 software (SAS, Raleigh, NC).

Given the equivalences of the overall mortality rate, we analyzed the proportion of deaths at each time point attributable to specific causes. Given the broad spectrum of action of azithromycin, we were particularly interested in the impact on infectious causes of death. Pneumonia and diarrheal diseases were therefore combined accordingly.

### Sample size calculation

The pre-specified sample size calculation found that the inclusion of approximately 620 communities would provide 80% power to detect 10% difference in all-cause mortality between the azithromycin versus the placebo arms. This assumes that the average community size would be 600–800 residents, of whom 16.7–19% would be children aged 1–59 months, and that the coefficient of variation would be between 0.4 and 0.51.

A sample size was not pre-specified for differences in the mortality by individual causes, as the sample size was driven by the all-cause mortality aim. The power to detect differences between the azithromycin and placebo arms, or in specific age groups, was less than that for the all-cause mortality analysis.

### Ethical review and trial oversight

Ethical approval was obtained from the Tanzanian National Institute for Medical Research and the Institutional Review Boards of the Johns Hopkins School of Medicine and University of California San Francisco. Children were included in the study on the basis of documented written informed consent from guardians. The study is registered at clinical trials.gov (NCT02048007). A data and safety monitoring committee provided trial oversight.

## Results

A total of 614 communities were randomized to azithromycin or placebo (Fig 1). The respective numbers of children in the ages of interest per community (56±29 vs. 58±33) and coverage i.e. proportion of eligible children at time of a given survey that received azithromycin or placebo (89.9±14.1% vs. 90.5±12.7%) were not statistically different.

Overall, 190 deaths were reported in the azithromycin arm and 200 deaths were reported in the placebo arm, in 32,514 and 33,638 person-years of follow up respectively (Fig 1). This corresponds to 0.58 and 0.59 deaths/100 person- years for the azithromycin and placebo arms respectively over the course of the study. Aggregating at community level, the median (IQR) rates were 0.0 (0.00, 0.99) per 100 person-years of follow-up in the azithromycin arm and 0.0 (0.0, 1.03) in the placebo arm (median two-sample test p = 0.51).

**Fig 3:**
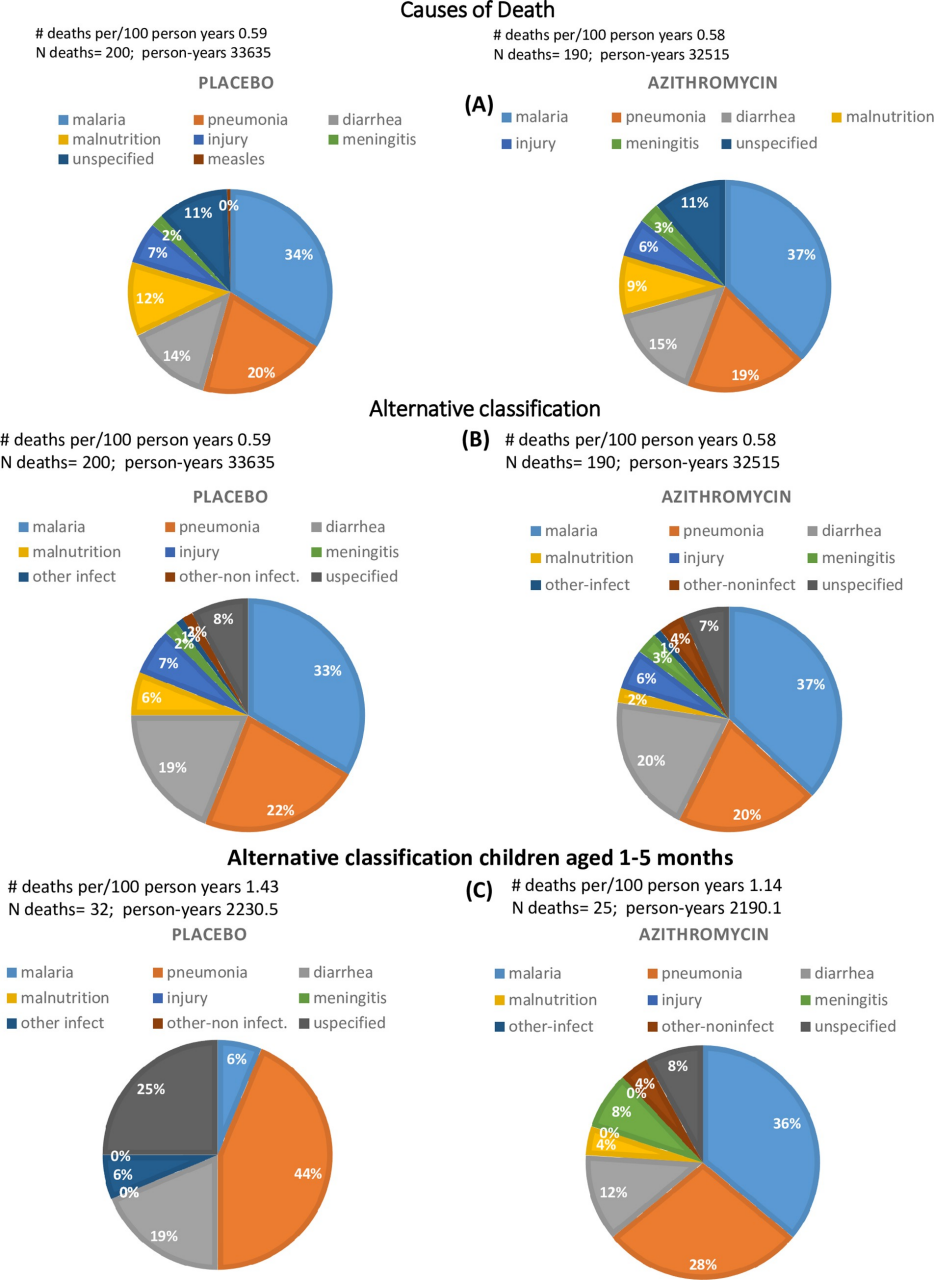
Causes of death. Causes of death among study participants as determined using the Black classification (A), using an alternative classification of malnutrition deaths (B) and alternative classification restricted to children aged 1-5months (C).

There were no significant differences in cause of death by treatment assignment (Fig 3). The leading causes of death in both the azithromycin and placebo arms were malaria (37% vs 34%), pneumonia (19% vs 20%) and diarrhea (15% vs 14%), together accounting for 71% and 68% of deaths in the respective arms (Fig 3A). Malnutrition accounted for 9% and 12% of deaths in the azithromycin and placebo arms respectively using the modified computer-based algorithm [11]. Following reclassification of malnutrition cases, malaria, pneumonia and diarrhea accounted for 78% and 76% of deaths in the azithromycin and placebo arms respectively (Fig 3B). A decline in overall mortality was shown in both arms over time (Table 1). A difference by treatment assignment was not significant after adjusting for community clustering.

**Table 1:** Mortality all causes by age group and arm.

Age group	Placebo			Azithromycin			Incidence rate ratio* (95% CI)	Reduction in mortality	P-value
	# deaths	Person years	Incidence rate per 100 person years	# deaths	Person years	Incidence rate per 100 person years			
1–5 months	32	2230.5	1.43	25	2190.1	1.14	0.80 (0.46–1.39)	20% (-39% to 54%)	0.42
6–11 months	45	3597.1	1.25	42	3599.2	1.17	0.92 (0.57–1.48)	8% (-48% to 43%)	0.73
12–23 months	60	7926.0	0.76	59	7564.1	0.78	1.03 (0.72–1.48)	-3% (-48% to 38%)	0.87
24–59 months	63	19888.5	0.32	64	19165.5	0.33	1.05 (0.74–1.50)	-5% (-50% to 26%)	0.78

*From age group specific negative binomial regression models at the community level.

For those children aged 1–5 months, 32 deaths occurred in the placebo arm and 25 deaths in the azithromycin arm representing a 20% 95% CI (-39% to 54%) reduction in mortality observed in the azithromycin arm (Table 1). Of the total numbers of deaths in this age stratum, 20 of the 32 deaths (62.5%) in the placebo arm and 10 of 25 (40%) in the azithromycin arm were attributed to diarrhea or pneumonia (Table 2). Regression models at community level suggest reduction in cause-specific mortality due to diarrhea/pneumonia in the youngest age group; nonetheless, this was not statistically significant by treatment assignment (Table 2). Further, an increased risk in the older age group was suggested; however, this was also not significant by treatment assignment (Table 2).

**Table 2:** Mortality due to pneumonia/diarrhea by age group and arm.

Age group	Placebo			Azithromycin			Incidence rate ratio* (95% CI)	Reduction in mortality	P-value
	# deaths	Person years	Incidence rate per 100 person years	# deaths	Person years	Incidence rate per 100 person years			
1–5 months	20	2230.5	0.90	10	2190.1	0.46	0.51 (0.23–1.13)	49% (-13% to 77%)	0.10
6–11 months	20	3597.1	0.56	17	3599.2	0.47	0.81 (0.40–1.67)	19% (-67% to 60%)	0.57
12–23 months	23	7926.0	0.29	22	7564.1	0.29	1.00 (0.56–1.80)	0% (-80% to 44%)	0.99
24–59 months	20	19888.5	0.10	28	19165.5	0.15	1.45 (0.81–2.59)	-45% (-159% to 19%)	0.21

*From age group specific negative binomial regression models at the community level.

## Discussion

Community-based azithromycin administration to children aged 1–59 months did not confer a difference in mortality or in the distribution of causes of mortality over time. The causes of death in both the treatment and placebo arms were overwhelmingly infectious in nature, with malaria, diarrheal disease and pneumonia accounting for over two thirds of reported deaths. In contrast, non-infectious causes (i.e. injury) accounted for less than a tenth of reported deaths. In those children aged less than 6-months, the findings suggested a difference in deaths attributable to pneumonia and diarrheal disease by treatment assignment. However, with so few deaths in this age group, the study was underpowered to detect a significant difference. In the other MORDOR countries (Niger and Malawi) [8], the strongest protective effect was observed in children under 6 months who received azithromycin, and our data suggest that further research should target this age group and—specifically—should evaluate protection against pneumonia and diarrheal disease.

Malaria accounted for a third of trial deaths independent of treatment assignment. Kilosa district is known to be endemic for malaria; the prevalence as defined by rapid diagnostic test (RDT) and peripheral smear positivity is 17.5% and 14.2% respectively [15]. However, attribution of death to malaria is complicated because the clinical presentation overlaps with a number of other disorders, potentially rendering clinical diagnosis—and certainly a verbal autopsy attribution—unreliable. In a study in Northern Tanzania, 60.7% of children who presented with fever were diagnosed with malaria, yet only 1.6% were ultimately confirmed as such [16]. Another study, of febrile children who presented to a clinic in Dar es Salaam and underwent comprehensive clinical and laboratory evaluation found over two thirds (70.5%) of presentations to be due to a viral etiology. Nonetheless, an analysis of participating children (n = 1030) at baseline (i.e. prior to intervention) in the Tanzanian arm of the MORDOR study found that almost a fifth (18.1%) were malaria RDT positive, lending support to malaria’s being a major mortality risk in this population [17].

A similar spectrum of cause of death was reported in the Malawi and Niger sites of the MORDOR trial. In Malawi, the leading causes of death were malaria (~44%), HIV/AIDS (~15%), pneumonia (~15%) and diarrhea (~8%), collectively accounting for 78–83% of deaths depending on which algorithm was used to ascertain cause of death [18]. With the exception of deaths associated with HIV/AIDS, the distribution of causes of death was similar between the azithromycin and placebo arms of the trial. In Niger, the leading causes of death were malaria (~28%), pneumonia (~16%) and diarrhea (~15%) [19]. However, those communities that received azithromycin demonstrated significantly lower number of deaths due to malaria, dysentery, meningitis and pneumonia.

This study highlights the challenges surrounding classification of death in low resource settings, which have implications for surveillance, resource allocation, public health planning and intervention. In 2010, only 2.7% of deaths in children younger than 5 years were medically certified [2]. The verbal autopsy is a valuable tool in this regard, comprising a standardized interview-based tool, whereby the cause of death is gleaned through questioning those in close contact with the deceased, such as a parent or guardian. The verbal autopsy has been designed specifically for use in countries where vital reporting systems are lacking [20]. One of the early iterations was validated in Cebu, Philippines by correlating symptoms and signs of illness using standardized questionnaires, with the actual cause of death, as determined by formal clinical, laboratory and radiographic determination [20]. This enabled categorization into one of a limited number of causes of death. The algorithms have been since been refined to optimize sensitivity and specificity [21].

However, the verbal autopsy is far from perfect [22]. First, its sensitivity and specificity for a given diagnosis or diagnostic category is highly variable. In the original validation study in Philippines [20], the verbal autopsy was highly sensitive (98%) and specific (90%) for measles, yet had comparatively low sensitivity (86%) and specificity (47%) for the diagnosis of acute lower respiratory tract infection (ARI)(20). Similarly, other causes of death—notably malaria, gastroenteritis, and meningitis—were found to have sensitivities less than 50% [23]. In part, this variation is inherent to the diagnosis or diagnostic category. For example, clinical measles has a relatively discrete presentation with characteristic rash and mucosal findings. In contrast, ARI or diarrheal disease may be the manifestation of a range of pathologies. The probability of a diagnostic category also changes depending on the rates of diseases in populations (e.g. measles is currently a rare disease where vaccination rates are high) and location of assessment. The original verbal autopsy did not include certain disease categories that are highly prevalent in other locations (e.g. malaria in Africa). Therefore, the autopsy should be adapted to the local setting and updated periodically. When optimized accordingly, a reasonable degree of correlation with laboratory confirmed diagnosis is achievable [23]. Similarly, one can obtain expert agreement: in one study in Kenya, a consensus diagnosis (i.e. agreement between three doctors with local pediatric experience) was reached in 296 of 303 (98%) of cases [23].Another consideration is that the diagnostic categories are not mutually exclusive, as for example, malnutrition which may be part of a pneumonia or diarrheal disease syndrome [24, 25]. Finally, we found that an arbitrary selection of duration of symptoms complicated the categorization; for example, a child who died within five days of diarrheal disease onset had the cause of death default to “malnutrition” because the duration was not longer than 14 days.

Other factors impact the reliability of the verbal autopsy. The educational level of the interviewer and interviewee as well as nuanced aspects of the culture will impact the rapport, interpretation and reliability of the responses. The instrument should ideally be validated in the target setting, with translation into the local vernacular and modification of phraseology guided by local input. Success is contingent on the skills of the interviewer, recognizing the challenges of communication with bereaved family members, in low-resourced settings, sometimes months (1–6 months in our study) following the event. Ideally, the primary caregiver (e.g. mother) is interviewed. However, one must sometimes rely on relatives or a neighbor, introducing unknown bias.

Our results are similar to a large, household-randomized trial conducted in Burkina Faso and Mali, which reported that the addition of azithromycin to seasonal malaria chemoprophylaxis(4 three-day cycles at monthly intervals during malaria season) failed to reduce deaths and hospital admissions in pre-school children (i.e. aged 3 months to 5 years). They did observe a lower burden of gastrointestinal infections, upper respiratory tract infections and nonmalarial febrile illnesses in the azithromycin arm with this intensive treatment regimen [26]. One could argue for a benefit of all children in a community receiving azithromycin, rather than randomization at household level where children from a placebo household might infect children from an azithromycin household; nevertheless, even with different randomization platforms, we observed similar results. There may still be an argument to be made for further research targeting children 1–6 months with azithromycin, and if shown to be effective in reducing infectious causes of deaths, to consider implementation alongside malaria interventions, vaccination or intermittent preventive treatment of malaria in infancy.

The study had limitations, some of which have previously described [8]. One is the structure of the algorithm itself. Given the need for consistency, there was adherence to stringent criteria for categorization of cause of death. Those criteria are somewhat arbitrary as described above. Second was the decision to include deaths as being study vs. non-study related based on prescribed inclusion criteria. In selected cases this was challenging given migration into and out of the communities; given that a childhood death is a rare event, inclusion or exclusion has a disproportionate effect on the results. As noted, if the child was “not in the village” due to being in hospital or clinic at the time of census and subsequently died, we counted that death. While randomization should control for these effects, loss of a death affects power to detect differences. A significant limitation is the small sample sizes in both the cause-specific and age strata. The trial assumed 80% power to detect a 10% lower mortality, assuming a mortality rate between 14 and 20 deaths/1000 person-years. While we found a 49% lower mortality rate in children aged 1–6 months due to pneumonia and diarrhea, the actual death rates were 9/1,000 person-years for the placebo arm and 4.5/1,000 in the azithromycin arm. With small numbers of deaths, these differences were not significant after adjusting for community clustering.

In conclusion, a significant reduction in mortality, and causes of mortality in children aged 1–59 months was not shown in the Tanzanian arm of the MORDOR study. Although 49% lower mortality rates attributable to diarrhea or pneumonia in those children age less than 6-months old were found in the azithromycin arm, this was not statistically significant. However, further investigation of these causes as the potential rationale for the decline in deaths in this age group is warranted in future trials in high mortality risk populations.

## Supporting information

S1 Checklist CONSORT 2010 checklist of information to include when reporting a randomised trial*. (DOCX)

S1 Data (XLSX)

S1 File (PDF)

S2 File (DOCX)

## References

[1] UN. Progress towards the Sustainable Development Goals: Report of the Secretary-General. United Nations:; 2017 11 May 2017. Contract No.: E/2017/66.

[2] Liu L, Johnson HL, Cousens S, Perin J, Scott S, Lawn JE, et al. Global, regional, and national causes of child mortality: an updated systematic analysis for 2010 with time trends since 2000. Lancet. 2012;379(9832):2151–61. doi: 10.1016/S0140-6736(12)60560-1 22579125

[3] Gething PW, Casey DC, Weiss DJ, Bisanzio D, Bhatt S, Cameron E, et al. Mapping Plasmodium falciparum Mortality in Africa between 1990 and 2015. N Engl J Med. 2016;375(25):2435–45. doi: 10.1056/NEJMoa1606701 27723434 PMC5484406

[4] UN. Sustainable Development Goals: 3 Good Health and Well-being 2018 [cited 2018 November 2]. Available from: https://www.un.org/sustainabledevelopment/health/.

[5] Golding N, Burstein R, Longbottom J, Browne AJ, Fullman N, Osgood-Zimmerman A, et al. Mapping under-5 and neonatal mortality in Africa, 2000–15: a baseline analysis for the Sustainable Development Goals. Lancet. 2017;390(10108):2171–82. doi: 10.1016/S0140-6736(17)31758-0 28958464 PMC5687451

[6] UNICEF. Under-five mortality 2018 [cited 2018 November 1]. Available from: https://data.unicef.org/topic/child-survival/under-five-mortality/.

[7] Liu L, Oza S, Hogan D, Perin J, Rudan I, Lawn JE, et al. Global, regional, and national causes of child mortality in 2000–13, with projections to inform post-2015 priorities: an updated systematic analysis. Lancet. 2015;385(9966):430–40. doi: 10.1016/S0140-6736(14)61698-6 25280870

[8] Keenan JD, Bailey RL, West SK, Arzika AM, Hart J, Weaver J, et al. Azithromycin to Reduce Childhood Mortality in Sub-Saharan Africa. N Engl J Med. 2018;378(17):1583–92. doi: 10.1056/NEJMoa1715474 29694816 PMC5849140

[9] Dunne MW, Singh N, Shukla M, Valecha N, Bhattacharyya PC, Dev V, et al. A multicenter study of azithromycin, alone and in combination with chloroquine, for the treatment of acute uncomplicated Plasmodium falciparum malaria in India. J Infect Dis. 2005;191(10):1582–8. doi: 10.1086/429343 15838784

[10] van Eijk AM, Terlouw DJ. Azithromycin for treating uncomplicated malaria. Cochrane Database Syst Rev. 2011(2):Cd006688. doi: 10.1002/14651858.CD006688.pub2 21328286 PMC6532599

[11] Kalter HD, Roubanatou AM, Koffi A, Black RE. Direct estimates of national neonatal and child cause-specific mortality proportions in Niger by expert algorithm and physician-coded analysis of verbal autopsy interviews. J Glob Health. 2015;5(1):010415. doi: 10.7189/jogh.05.010415 25969734 PMC4416334

[12] Black RE, Cousens S, Johnson HL, Lawn JE, Rudan I, Bassani DG, et al. Global, regional, and national causes of child mortality in 2008: a systematic analysis. Lancet. 2010;375(9730):1969–87. doi: 10.1016/S0140-6736(10)60549-1 20466419

[13] Johnson HL, Liu L, Fischer-Walker C, Black RE. Estimating the distribution of causes of death among children age 1–59 months in high-mortality countries with incomplete death certification. Int J Epidemiol. 2010;39(4):1103–14. doi: 10.1093/ije/dyq074 20519334

[14] WHO. International standard verbal autopsy questionnaires: verbal autopsy standards—ascertaining and attributing cause ofdeath (death of a child aged 4 weeks to 14 years). Geneva.

[15] Mazigo HD, Rumisha SF, Chiduo MG, Bwana VM, Mboera LEG. Malaria among rice farming communities in Kilangali village, Kilosa district, Central Tanzania: prevalence, intensity and associated factors. Infect Dis Poverty. 2017;6(1):101. doi: 10.1186/s40249-017-0315-1 28676077 PMC5497374

[16] Crump JA, Morrissey AB, Nicholson WL, Massung RF, Stoddard RA, Galloway RL, et al. Etiology of severe non-malaria febrile illness in Northern Tanzania: a prospective cohort study. PLoS Negl Trop Dis. 2013;7(7):e2324. doi: 10.1371/journal.pntd.0002324 23875053 PMC3715424

[17] Bloch EM, Kasubi M, Levin A, Mrango Z, Weaver J, Munoz B, et al. Babesia microti and Malaria Infection in Africa: A Pilot Serosurvey in Kilosa District, Tanzania. Am J Trop Med Hyg. 2018;99(1):51–6. doi: 10.4269/ajtmh.18-0012 29637884 PMC6085789

[18] Hart JD, Kalua K, Keenan JD, Lietman TM, Bailey RL. Effect of Mass Treatment with Azithromycin on Causes of Death in Children in Malawi: Secondary Analysis from the MORDOR Trial. The American journal of tropical medicine and hygiene. 2020;103(3):1319–28. doi: 10.4269/ajtmh.19-0613 32342837 PMC7470551

[19] Keenan JD, Arzika AM, Maliki R, Elh Adamou S, Ibrahim F, Kiemago M, et al. Cause-specific mortality of children younger than 5 years in communities receiving biannual mass azithromycin treatment in Niger: verbal autopsy results from a cluster-randomised controlled trial. Lancet Glob Health. 2020;8(2):e288–e95. doi: 10.1016/S2214-109X(19)30540-6 31981558 PMC7025321

[20] Kalter HD, Gray RH, Black RE, Gultiano SA. Validation of postmortem interviews to ascertain selected causes of death in children. Int J Epidemiol. 1990;19(2):380–6. doi: 10.1093/ije/19.2.380 2376451

[21] Mpimbaza A, Filler S, Katureebe A, Quick L, Chandramohan D, Staedke SG. Verbal Autopsy: Evaluation of Methods to Certify Causes of Death in Uganda. PLoS One. 2015;10(6):e0128801. doi: 10.1371/journal.pone.0128801 26086600 PMC4472780

[22] Garenne M, Fauveau V. Potential and limits of verbal autopsies. Bull World Health Organ. 2006;84(3):164. doi: /S0042-96862006000300004 16583068 PMC2627293

[23] Snow RW, Armstrong JR, Forster D, Winstanley MT, Marsh VM, Newton CR, et al. Childhood deaths in Africa: uses and limitations of verbal autopsies. Lancet. 1992;340(8815):351–5. doi: 10.1016/0140-6736(92)91414-4 1353814

[24] Rice AL, Sacco L, Hyder A, Black RE. Malnutrition as an underlying cause of childhood deaths associated with infectious diseases in developing countries. Bull World Health Organ. 2000;78(10):1207–21. 11100616 PMC2560622

[25] Ibrahim MK, Zambruni M, Melby CL, Melby PC. Impact of Childhood Malnutrition on Host Defense and Infection. Clin Microbiol Rev. 2017;30(4):919–71. doi: 10.1128/CMR.00119-16 28768707 PMC5608884

[26] Chandramohan D, Dicko A, Zongo I, Sagara I, Cairns M, Kuepfer I, et al. Effect of Adding Azithromycin to Seasonal Malaria Chemoprevention. New England Journal of Medicine. 2019;380(23):2197–206. doi: 10.1056/NEJMoa1811400 30699301

