# Supplementary material for: Causes of death after biannual azithromycin treatment: A community-level randomized clinical trial
Source: PLoS One. 2021 Sep 24;16(9):e0250197. doi: 10.1371/journal.pone.0250197 (PMC8462712; doi:10.1371/journal.pone.0250197)
Supplement: S1 File — (PDF) [file pone.0250197.s003.pdf]

Protocol

This trial protocol has been provided by the authors to give readers additional information about their work.

Protocol for: Keenan JD, Bailey RL, West SK, et al. Azithromycin to reduce childhood mortality in sub-Saharan Africa. N Engl J Med 2018;378:1583-92. DOI: 10.1056/NEJMoa1715474

This supplement contains the following items:

1. Original protocol, final protocol, summary of changes.
2. Original statistical analysis plan, final statistical analysis plan, summary of changes.

Mortality Reduction After Oral Azithromycin

Manual of Operations and Procedures

The Francis I. Proctor Foundation, Global Health Sciences
University of California, San Francisco
The Carter Center
Niger Ministry of Health

The Dana Center, Bloomberg School of Public Health
Johns Hopkins University
Tanzania National Institute for Medical Research and Muhimbili Medical Center

The London School of Hygiene & Tropical Medicine
College of Medicine, University of Malawi, Blantyre
Malawi Ministry of Health

Table of Contents

ABBREVIATIONS **5**

CHAPTER 1: OVERVIEW **6**

1.1. EXECUTIVE SUMMARY 6
1.2. OBJECTIVES 6
1.3. STUDY SITES 7

CHAPTER 2: CONTEXT **7**

CHAPTER 3: STUDY DESIGN **10**

3.1. RANDOMIZATION 11

CHAPTER 4: STUDY ELIGIBILITY **11**

4.1. ELIGIBLE COMMUNITIES 11
4.2. ELIGIBLE INDIVIDUALS 12
4.3. STUDY SCHEDULE 13

CHAPTER 5: CORE AND NON-CORE STUDY ELEMENTS **14**

5.1. MORTALITY STUDY 14
5.1.1. Core Elements (Malawi, Niger, and Tanzania) *14*
5.2. MORBIDITY/RESISTANCE STUDY 15
5.2.1. Core Elements (Malawi, Niger, and Tanzania) *15*
5.2.2. Non-core Elements *15*

CHAPTER 6: CENSUS **16**

6.1. CENSUS 16
6.2. RANDOM CENSUS VERIFICATION 18
6.3. VERBAL AUTOPSY 19
6.4. VALIDATING THE MORTALITY OUTCOME 20
6.4.1 Niger *20*
6.4.2 Malawi *21*
6.4.3 Tanzania *22*

CHAPTER 7: REGISTERING PARTICIPANTS FOR SPECIMEN COLLECTION **22**

CHAPTER 8: BLOOD SAMPLES **23**

8.1. FINGERSTICK 23
8.2. DRIED BLOOD SPOT FOR MALARIA 24
8.3. HEMOGLOBIN TEST 25
8.4. THICK AND THIN SMEARS FOR MALARIA 27
8.5. MATERIALS FOR BLOOD COLLECTION 27

CHAPTER 9: SPECIMEN COLLECTION FOR RESISTANCE TESTING **28**

9.1. POPULATION 28
9.2 NASOPHARYNGEAL SWABS 29
9.2.1 Materials for Swab Collection for Resistance Testing *29*
9.2.2 Protocol for Tubing and Handling of Samples *30*
9.3 STOOL SAMPLES 31
9.3.1 Stool Specimen Collection *31*
9.3.2 Materials for Stool Specimen Collection *33*
9.4 QUALITY CONTROL MEASURES FOR SPECIMEN COLLECTION 34

CHAPTER 10: COST-EFFECTIVENESS 34
10.1. COSTS 34
10.2 EFFECTIVENESS 34
CHAPTER 11: TRAINING 35
11.1 STANDARDIZATION 35
11.2 PILOT STUDIES 35
CHAPTER 12: SAMPLE ORGANIZATION, TRANSPORT, AND STORAGE 36
12.1 DE-IDENTIFICATION 36
12.2 SPECIMEN TRANSPORT 36
12.3 SPECIMEN STORAGE 37
12.3.1 *Short-term Sample Storage* *37*
12.3.2 *Long-term Sample Storage* *37*
12.4 CATALOG SPECIMENS 38
CHAPTER 13: MICROBIOLOGY AND MOLECULAR LABORATORY PROCEDURES 39
13.1 STORAGE AND TRANSPORT MEDIA 39
13.1.1 *Preparation of STGG Transport Medium* *39*
13.1.2 *Preparation of Amies Transport Medium* *40*
13.1.3 *Preparation of Nutrient Agar Medium/Slants* *40*
13.2 METHODS FOR MALARIA TESTING 41
13.2.1 *Blood Smears* *41*
13.2.2 *Dried Blood Spots* *41*
13.3 METHODS FOR RESISTANCE TESTING 47
13.3.1 *Isolation & Identification of Organisms Using Microbiological Techniques* *47*
13.3.2 *Microbiological Resistance Testing for Swabs* *49*
13.3.3 *Nucleic Acid Based Resistance Testing for Stool Specimens* *51*
13.4 METHODS FOR *CHLAMYDIA TRACHOMATIS* (NON-CORE) 53
13.4.1 *Description of Abbott RealTime Assay* *53*
13.4.2 *Processing of Samples for PCR* *53*
13.4.3 *Procedure for Masking Samples* *53*
13.4.4 *Procedure for Pooling Samples* *54*
13.4.5 *Quality Control* *54*
13.4.6 *Quantification* *54*
CHAPTER 14: STUDY MEDICATION 55
14.1 STUDY MEDICATION DESCRIPTION (FROM PFIZER, INC.) 55
14.1 DOSAGE INFORMATION 55
14.2 MEDICATION PROCUREMENT/DONATION 55
14.3 MEDICATION QUALITY CONTROL 55
14.4 ANTIBIOTIC DISTRIBUTION & MONITORING COVERAGE 56
14.5 ADVERSE REACTIONS/SIDE EFFECTS 56
14.6 ADVERSE EVENTS SYSTEMS 57
14.6.1 *Passive Adverse Events Monitoring* *57*
14.6.2 *Active Adverse Events Monitoring* *57*
14.6.3 *Adverse Events Data* *59*
14.8 SUPPLY ISSUES 59
CHAPTER 15: PROTECTION OF HUMAN SUBJECTS 59
15.1 INSTITUTIONAL REVIEW BOARD APPROVAL 60

15.1.1. Niger *60*
15.1.2 Tanzania *60*
15.1.3 Malawi *60*
15.2 INFORMED CONSENT 60
15.3 RISKS AND BENEFITS OF STUDY PROCEDURES 61
15.3.1 Verbal Autopsy *61*
15.3.2 Swabbing Procedures *61*
15.3.3 Stool Collection *61*
15.3.4 Blood Testing *61*
15.3.5 Anthropometric Measurements *62*

CHAPTER 16: STUDY MONITORING 62

CHAPTER 17: DATA AND SAFETY MONITORING COMMITTEE CHARTER 62

17.1 PRIMARY RESPONSIBILITIES OF THE DSMC 62
17.2 DSMC MEMBERSHIP 63
17.3 CONFLICTS OF INTEREST 63
17.4 TIMING AND PURPOSE OF THE DSMC MEETINGS 63
17.5 PROCEDURES TO ENSURE CONFIDENTIALITY AND PROPER COMMUNICATION 64
17.6 STATISTICAL MONITORING GUIDELINES 65
17.7 DSMC CONTACT INFORMATION 66

CHAPTER 18: DATA COLLECTION, MANAGEMENT, AND SECURITY 67

18.1. SCOPE OF DATA 67
18.2 DATA STORAGE, MANAGEMENT, AND SECURITY 67

REFERENCES 69

APPENDIX 73

Abbreviations

DCC: Data Coordinating Center
GPS: global positioning system
IRB: Institutional Review Board
JHU: The Johns Hopkins University
LSHTM: London School of Hygiene and Tropical Medicine
MUAC: mid-upper arm circumference
NP swabs: nasopharyngeal swabs
PCR: polymerase chain reaction
STGG: skim milk tryptone glucose glycerin media
TF: Trachomatous Inflammation – Follicular
TI: Trachomatous Inflammation – Intense
TS: Trachomatous Scarring
TT: Trachomatous Trichiasis
UCSF: University of California San Francisco
WHO: World Health Organization

Chapter 1: Overview

1.1. Executive Summary

An estimated 7.7 million pre-school aged children die each year, the majority from infectious diseases.[1] Recently, we demonstrated that mass azithromycin distributions for trachoma may have the unintended benefit of reducing childhood mortality.[2,3] This surprising result was observed in an area of Ethiopia with highly prevalent trachoma. Another trial is necessary to determine whether a similar effect will be seen in areas not eligible for trachoma treatments. If mass azithromycin can be proven to reduce childhood mortality, then widespread implementation in developing countries could be feasible and perhaps integrated with other interventions.

Our long-term goal is to more precisely define the role of mass azithromycin treatments as an intervention for reducing childhood morbidity and mortality. We propose a multi-site, cluster-randomized trial comparing communities randomized to oral azithromycin with those randomized to placebo. To assess the generalizability of the intervention, we will monitor for antibiotic resistance, which could potentially limit adoption of mass antibiotic treatments. We will also begin to investigate possible explanatory factors for mortality reduction by assessing several measures of the burden of infectious diseases. We hypothesize that mass azithromycin treatments will reduce childhood mortality even in areas with low trachoma prevalence, and will be accompanied by an acceptable level of antibiotic resistance.

1.2. Objectives

1: To test the hypothesis that mass distributions of oral azithromycin targeted to 1-60 month old children reduces childhood mortality, in a cluster-randomized trial

2: To assess the cost-effectiveness of mass azithromycin in reducing childhood mortality

3: To investigate the most plausible factors that could explain an effect of mass azithromycin on childhood mortality

4: To assess for emergent macrolide resistance among children following mass azithromycin treatments

5: To collect and securely store samples from the nasopharynx, nares, conjunctiva, blood, and stool after azithromycin distributions to assess infectious burden and resistance

Ancillary Aim 1 (non-core): To assess the effect of mass azithromycin treatments on microbial diversity in the conjunctival, nasopharyngeal, nasal, and intestinal biome of children

1.3. Study Sites

A consortium of experienced researchers and program implementers will conduct the study in 3 sites (Malawi, Niger, and Tanzania), chosen for diversity of geography and comorbidities, existence of a functioning national azithromycin distribution program, and previous investigator experience:

- **Southern Africa:** Mangochi district, Malawi (Malawi Ministry of Health, University of Malawi, Blantyre, and London School of Hygiene & Tropical Medicine)
- **West Africa:** Loga and Boboye departments, Niger (Carter Center Niger, Niger Ministry of Health, University of California, San Francisco)
- **East Africa:** Kilosa district, Tanzania (National Institute for Medical Research, Kilosa, Johns Hopkins University)

Chapter 2: Context

Reduction of childhood mortality is an international development goal. The United Nations Millennium Development Goals call for a two-thirds reduction in childhood mortality by 2015.[4] Despite progress this past decade, under-5 mortality remains unacceptably high.[1,5,6] In Africa, where the burden of childhood mortality is greatest, three-quarters of all childhood deaths occur after the neonatal period, with malaria, pneumonia, and diarrhea accounting for the majority.[7] Management is often case detection and individualized therapy, but novel strategies such as mass treatment may play a role.[8-10]

The proposed research will assess whether childhood mortality can be reduced using a novel approach—mass antibiotic administrations similar to those used for trachoma. The World Health Organization (WHO) recommends repeated annual distribution of single-dose oral azithromycin to each resident of trachoma-endemic communities, though more frequent treatments may be necessary.[11-13] While azithromycin effectively clears the ocular strains of chlamydia that cause trachoma,[12,14,15] the drug also has activity against major causes of childhood mortality, including respiratory infections, diarrhea, and malaria.[16-18] Mass treatments have been shown to be effective for these non-chlamydial infections. For example, in a study we conducted in Nepal, the prevalence of diarrhea in children decreased from 32% before a mass

azithromycin treatment, to 11% ten days post-treatment (OR 0.26 [95% CI 0.14 – 0.43]).[19] Others in our consortium have found that malaria parasitemia decreased by 44% after mass azithromycin treatments.[20] It is feasible that periodic mass azithromycin distributions could reduce the burden of these infections sufficiently enough to reduce childhood mortality.[2]

Mass azithromycin treatments targeted to children may maximize the benefit of antibiotics, while limiting resistance. Pre-school children have the most to gain from mass azithromycin treatment because the burden of treatable, life-threatening infection is highest in this age group.[21] Treatment of children may also provide indirect protection to untreated members of the community due to a herd effect.[22] In a trial in Ethiopia, quarterly mass azithromycin treatments targeted to children aged 1-10 years resulted in a 47% reduction in ocular chlamydia in *untreated* community members.[23] Although we demonstrated this indirect protection for ocular chlamydial infection, other infectious diseases may be similar. Reducing the infectious burden of respiratory pathogens, malaria, and diarrhea might benefit children who missed a distribution or were too old for treatment. Given that community antibiotic resistance correlates with the volume of antibiotic consumption,[24-26] targeting children may result in less antibiotic resistance in a community. Targeting children would also be easier logistically and require fewer doses of donated antibiotic, both of which lower costs of administrations.

The finding of a reduction in childhood mortality after mass azithromycin treatment warrants replication in other settings to increase generalizability. The current proposal differs from our earlier trial in several important aspects. First, we will conduct the study in areas with little or no trachoma to enhance generalizability. Second, we will monitor a subset of communities for measures of infectious and nutritional morbidity, to identify explanatory factors for any mortality reduction. Third, we will randomize control communities to placebo, reducing the chance of differential co-intervention and differential outcome assessment. Fourth, we will treat children 1 month and older, as opposed to 12 months and older. Fifth, we will test whether restricting azithromycin to only children will limit selection of community antibiotic resistance in *Streptococcus pneumoniae* and other organisms. Sixth, we will monitor for a longer period of time than the previous study, to assess whether mass azithromycin results in a more sustained mortality reduction. Finally, we will enroll a larger number of children and randomize a larger number of clusters, providing considerably more statistical power to detect even a smaller reduction in mortality.

Mass drug administrations can be implemented relatively easily and potentially have a major impact on morbidity and mortality. Mass treatments

are simple interventions and considerable expertise in their deployment already exists in developing countries.[27] They can have a dramatic impact in some settings. For example, a study of mass distribution of vitamin A for xerophthalmia in the 1980s found that treated communities had lower childhood mortality compared to untreated communities.[28] Trials in the following decade largely confirmed these results.[29-34] Now, vitamin A distributions are provided in most countries that have high childhood mortality, with a majority of programs achieving the targeted 70% coverage.[35] A report estimated that vitamin A could prevent as many as a quarter million childhood deaths each year.[36] This same study estimated that treatment of diarrhea, sepsis, pneumonia, and malaria could prevent over 3 million deaths each year. If mass azithromycin treatments can prevent or treat even a fraction of these infections, this could have a tremendous effect on childhood mortality.

Millions of azithromycin treatments are currently distributed each year. Since 1999, over 225 million treatments of azithromycin have been donated through the International Trachoma Initiative for distribution by local partners in 19 countries.[37] Many Ministries of Health and non-governmental organizations have experience procuring and transporting the drug, organizing local health workers for antibiotic distribution, and monitoring antibiotic coverage. These activities have proven to be sustainable; excluding the cost of the antibiotic itself, mass distribution costs an estimated $0.25-0.40 per person treated.[38] Although the costs of azithromycin would add to this, generic azithromycin is available, and economies of scale would likely reduce drug costs if mass treatments became more widespread.

Chapter 3: Study Design

We will assess childhood mortality over two years, comparing communities where children aged 1-60 months receive biannual oral azithromycin (“Azithro” arm), to communities where the children receive biannual oral placebo (“Control” arm). We will also randomize a separate set of communities to these treatment strategies, but monitor instead for infectious disease morbidity and macrolide resistance (“Azithro-Plus" and "Control-Plus”). These communities will not be included in the mortality comparison, since identification and treatment of morbidities could interfere with any mortality effect of azithromycin. All biologic specimens collected as part of the morbidity and resistance outcomes will be stored and made available to other investigators for further laboratory testing at the conclusion of the study, per Gates Foundation guidelines.

Figure 1: Trial Profile

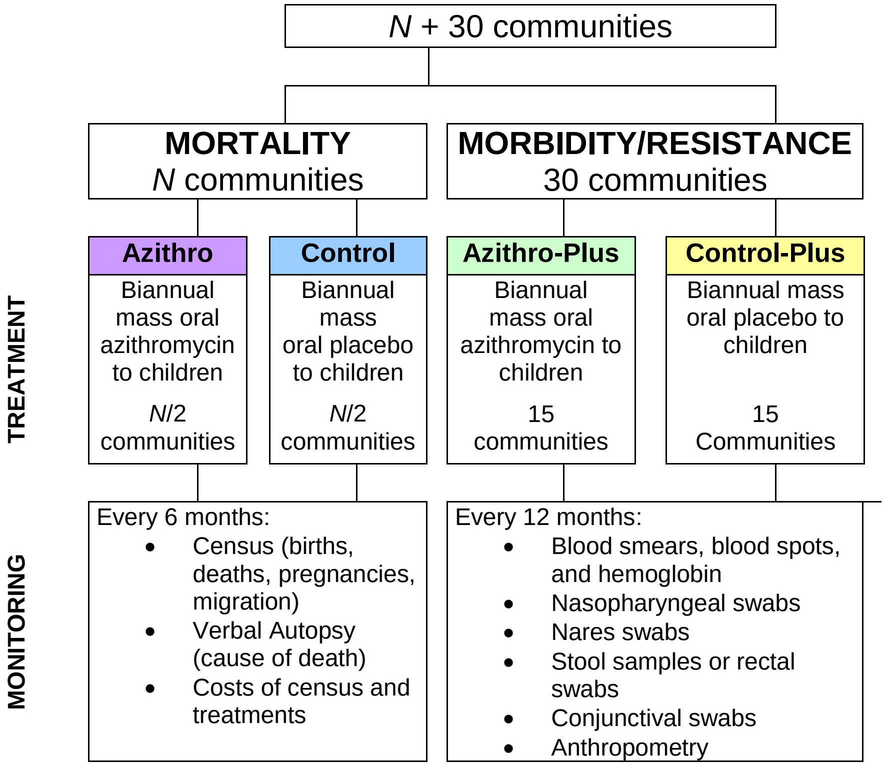

3.1. Randomization

Randomization of Treatment Allocation. In each site, communities within a contiguous area of 300,000 to 600,000 individuals will be randomized to azithromycin or placebo for the mortality study. An additional 30 communities will be randomized into one of two study arms for embedded, cost-efficient, morbidity studies. Since mortality and morbidity study communities will be drawn from the same pool, findings from one study will be relevant to the other. Each site has a different set of prevalent co-morbidities and different access to health interventions.

No stratification or block randomization is planned within each country. The allocation of community assignments will be conducted for each country separately using simple random sampling. For each country, all study arms (mortality and morbidity) will be assigned from the same list of eligible communities. A pseudorandom number scheme will be implemented using the statistics package R. Randomization will be conducted by T. Porco. Procedural and algorithmic details are provided in an appendix to the Statistical Analysis Plan.

Study Participants: At months 0, 12, and 24, a random sample of children will be selected using a computer-generated simple random sample for exams and sample collection to monitor for morbidity and resistance.

Chapter 4: Study Eligibility

4.1. Eligible Communities

To be eligible for the trial, a community must meet the following criteria:

1. The community location in target district.
2. The community leader consents to participation in the trial (this does not obviate the need for individual consent, but without overall leadership consent, the community as a whole cannot be part of the trial).
3. Eligible communities estimated population of between 200–2,000 people.
4. The community is not in an urban area.

4.2. Eligible Individuals

Mortality Study:

Census: The study can be thought of as consisting of four 6-month segments, each of which starts with a census and ends with a follow-up census. All children in the study communities aged 1-60 months (up to but not including the 5th birthday) at the initial census of each segment are eligible to participate in the subsequent 6-month segment of the study. Note that the information for children erroneously entered into the census (e.g., children younger than 1 month or ≥ 60months) can be corrected at the subsequent treatment, subsequent census, or at a verbal autopsy later in the study. However, these changes will not be applied retroactively; these misclassified children will still be included in the study population for that 6-month segment and any deaths will be counted toward the primary outcome. In addition, all children listed on the initial census for that 6-month segment will be included in the outcome, regardless of whether they received the study drug.

Treatment: Individuals allergic to macrolides or azalides will not be given the study antibiotic azithromycin, but will be included in the outcome.

Morbidity/Resistance Study:

Census: The criteria for being included in the census are the same as the Mortality Study, as described above.

Treatment: The inclusion and exclusion criteria for treatment are the same as for the Mortality Study, as described above.

Examination & Sample Collection: A random sample of children aged 1-60 months (up to but not including the 5th birthday) are eligible for examination and sample collection. As described above, the random sample will be a simple random sample based on the previous census. These individuals will likewise be selected from the previous census.

> **Note:** All individuals must be registered on the official census in order to be considered part of the study.

4.3. Study Schedule

The schedule for examination and treatment is shown below in Table 1: MORDOR Study Schedule

	MORTALITY		MORBIDITY / RESISTANCE	
	**Azithro**	**Control**	**Azithro-Plus**	**Control-Plus**
	**Oral Azithromycin (*N/2* communities)**	**Oral Placebo (*N/2* communities)**	**Oral Azithromycin (N = 15 communities)**	**Oral Placebo (N = 15 communities)**
**MORDOR 0 Fall 2014**	Census<br>Azithro	Census<br>Placebo	Census<br>Swabs<br>Blood<br>Stool<br>Anthropometry<br>Azithro	Census<br>Swabs<br>Blood<br>Stool<br>Anthropometry<br>Placebo
**MORDOR 6 Spring 2015**	Census<br>Verbal Autopsy<br>Azithro	Census<br>Verbal Autopsy<br>Placebo	Census<br>Azithro<br>*NP Swabs, Blood, Stool* [a]	Census<br>Placebo<br>*NP Swabs, Blood, Stool* [a]
**MORDOR 12 Fall 2015**	Census<br>Verbal Autopsy<br>Azithro	Census<br>Verbal Autopsy<br>Placebo	Census<br>Swabs<br>Blood<br>Stool<br>Anthropometry<br>Azithro	Census<br>Swabs<br>Blood<br>Stool<br>Anthropometry<br>Placebo
**MORDOR 18 Spring 2016**	Census<br>Verbal Autopsy<br>Azithro	Census<br>Verbal Autopsy<br>Placebo	Census<br>Azithro<br>*NP Swabs, Blood, Stool* [a]	Census<br>Placebo<br>*NP Swabs, Blood, Stool* [a]
**Malaria collection**			Blood[b]	Blood[b]
**MORDOR 24 Fall 2016**	Census<br>Verbal Autopsy<br>**Azithro**[c]	Census<br>Verbal Autopsy<br>**Azithro**[c]	Census<br>Swabs<br>Blood<br>Stool<br>Anthropometry<br>**Azithro**[c]	Census<br>Swabs<br>Blood<br>Stool<br>Anthropometry<br>**Azithro**[c]

Only children aged 1 month to 60 months per community will be treated
Arms Azithro+ & Control+: swabs, blood testing, and stool samples or rectal swabs from 40 randomly selected children aged 1 to 60 months per community; anthropometry from all children aged 1 to 60 months per community; NP swabs from 40 individuals aged 7 – 12 years (Niger only)

a: Tanzania only
b: Niger only
c: Decision to treat ALL communities with azithromycin at 24-months if there is a significant mortality difference (in preparation for possible contingent study, Niger only)

Chapter 5: Core and Non-Core Study Elements

An overview of core and non-core elements for the mortality and morbidity study is provided here, but will be described in more detail in the following chapters.

5.1. Mortality Study

5.1.1. Core Elements (Malawi, Niger, and Tanzania)

In all three study sites, we will conduct the following study activities for the mortality study:

Training

Before the baseline visit, the University of California San Francisco (UCSF) team will certify local team members at all three study sites. Standardization activities before each biannual census will consist of didactic classroom instruction and mock census activities, followed by in-field training to harmonize protocols.

Census

An enumerated population census for 0 – 60 month olds (focusing on this age group) will be conducted every 6 months by trained field workers masked to study arm, recording births, deaths, and migration of children eligible for treatment. Pregnant women will be noted at each census, to maximize inclusion of newborns on the subsequent census.

Random Census Verification

A repeat census will be conducted in a random selection of households at each study visit. Personnel will be different from the original census. This will allow us to assess whether the census team identified all births, deaths, and migratory episodes relative to the previous census.

Verbal Autopsy

Each of the study sites will perform a verbal autopsy for all children (aged 1-60 months) identified as having died during the study.[39]

Treatment

Children aged 1-60 months on the current census will be offered weight- or height-based (<1 year weight and children who cannot stand; ≥1 year height), directly observed, oral azithromycin suspension (or oral placebo) every 6 months for 2 years as performed in trachoma programs in each of the 3 countries. Specifically, individuals are eligible on or after their one month birthday, and prior to the day of their fifth birthday.

Antibiotic Coverage Surveillance
We will estimate antibiotic coverage from the most recent biannual census records. At the end of each treatment round at months 6, 12, and 18, we will identify any children who have missed 2 or more consecutive treatments, and forward this information to the census team.

Costs
We anticipate recording the cost of distributions in a random subset of 12 villages at the 6 or 12 and 24 month visits.

5.2. Morbidity/Resistance Study

5.2.1. Core Elements (Malawi, Niger, and Tanzania)

All core elements described in 5.1.1 will also be conducted in the morbidity / resistance study. The enumerated population census for the morbidity/resistance communities will include 0-12 year olds.
The designation "core elements" means that all study sites will be performing the study activity.

In all study sites, we will perform the following tests on a random sample of 40 children aged 1-60 months from each Azithro-Plus and Control-Plus community at baseline, 12, and 24 months:
- Blood samples for malaria and anemia
- Nasopharyngeal swabs for pneumococcal macrolide resistance
- Stool samples or rectal swabs to assess for macrolide resistance
- Assessment of costs of a census and mass drug administration (at 6 or 12 and 24 month visits only)

Samples will be processed in-country when possible; otherwise they will be shipped to UCSF.

5.2.2. Non-core Elements
The designation "non-core" element means that study sites may choose if they wish to perform the study activity. If the study sites perform the study activity, the methodology will be performed according to this protocol so that the 3 sites may be more easily compared. Sites may choose to perform several non-core morbidity assessments at the 0, 12, and 24 month visits:
- Ear swabs for chronic suppurative otitis media and microbiome analysis
- Nasal swabs for macrolide resistance in *Staphylococcus aureus*
- Conjunctival swabs for *Chlamydia trachomatis*
- Conjunctival examination and photography to assess for trachoma

- Anthropometric assessments
- Nasopharyngeal swab collection at local health posts to compare macrolide resistance in the community with that in the health post
- Symptom questionnaire of randomly selected children

In addition, there are several non-core activities that occur outside the annual morbidity/resistance assessment:
- Additional study visit during the first malaria season to collect blood samples for malaria and anemia testing
- Clinic-based case finding for childhood illnesses at all health clinics in the study area

Details of the non-core elements for each study site can be found in the Appendices.

Chapter 6: Census

6.1. Census

Census Team

Census workers will be selected by the site study coordinator. These individuals may have different qualifications and educational backgrounds in the 3 sites, but, at a minimum, each census team member should be computer-literate, such that they are able to operate a tablet computer and type on its keyboard. Census workers will ideally be local community dwellers, and familiar with the communities being enumerated. In addition, several supervisors will be present for the duration of the census to monitor census workers.

Census Training

Census workers will be trained at the beginning of the study and refresher trainings will be offered as needed for the duration of the study. Training will start with reviewing the census data collection software on the tablet computer, care of the tablets, charging of the tablets, etc. The training will then proceed to a demonstration of the use of the software at a mock household, including common problems that staff may encounter (e.g., no one at home, GPS function not working, software crashing). In the final part of the training, the study coordinators and investigators will accompany team members to several communities and observe the census activities.

Census Software

The census will be directly entered into a tablet computer. The software will capture information about each child aged 0-5 (or 0-12 in morbidity

communities) in each household: name, age, sex, father's name, and mother's name, and will also register any pregnant women. The GPS coordinates will be documented for each household at the entrance to the household. At follow-up censuses, team members will identify each household on the existing census, and will update the status for each child:

- STATUS: Alive, slept in household last night
- STATUS: Alive, but not in household
    - ABSENCE: <1 month
        - Is he/she coming back within 1 week?
            - Yes (mop-up)
            - No
            - I don't know
        - INFORMANT: household member, neighbor, village chief, other
    - ABSENCE: ≥ 1 month
        - MOVE: Moved within community
            - INFORMANT: household member, neighbor, village chief, other
        - MOVE: Moved outside of community
            - INFORMANT: household member, neighbor, village chief, other
- STATUS: Died
    - PLACE: Child living in community when died
        - INFORMANT: household member, neighbor, village chief, other
    - PLACE: Child had moved out of community when died
        - INFORMANT: household member, neighbor, village chief, other
- STATUS: Unknown
    - INFORMANT: household member, neighbor, village chief, other

Whenever a new individual is added to the census, the software will automatically assign each individual to a universal unique identification number as well as a study identification number.

Census Data Uploading

The census will be collected on tablet computers with 3G mobile and Wi-Fi capabilities. There will be 3 options for uploading data to the database. First, and most desirable, a local SIM card with data plan can be purchased for each tablet, and the data uploaded via cell towers twice per day (midday and at the end of the day). This option is most desirable because it minimizes data loss in the case of a lost, stolen, or damaged device. In addition, this option will not require each

tablet computer to be in contact with a Wi-Fi hub. As a second option for uploading, each census supervisor will have access to a Wi-Fi hub, and the supervisor can visit the census teams to upload data regularly. This option is less desirable, because the data will be uploaded less frequently. As a third option, the data can be uploaded at a central study site, either via Wi-Fi or micro USB cable directly into a computer. This option is least desirable because is not feasible to take the tablet computers to the central site regularly given the large geographical areas of the study.

Census Supervision
The site study coordinator will supervise all census activities. Formal checks of census quality will be conducted through the random census verification. In addition, the study coordinator will visualize all censused households using imagery from GoogleMaps. The goal of this activity will be to minimize the chances of missing large neighborhoods or specific regions within study communities. The study coordinator will also check the data entry progress for each community, paying special attention at the follow-up censuses, as to whether there are any missing data for the "vital status" variable (i.e., present, dead, absent). Once the study coordinator is confident that the entire community has been reached, and that the amount of missing data are acceptable (defined as <10% of children in a community), the study coordinator will "lock" the census data collection for that community, and no changes can made be made to the census until the next census. Changes can be made to the record at different time points, but these changes will not be reflected until the subsequent census. All changes will be time and date stamped in the database.

Census Timing
The census will be performed prior to each mass azithromycin/placebo distribution. Study sites may choose to perform the census activities over a discrete time period (e.g., all communities completed over a 1-month period, requiring census activities to take place simultaneously in many communities at once) or alternatively in a "rolling" fashion (e.g., all communities completed over a 6-month period, requiring fewer census teams to be active at once). In either case, each community must be censused every 6 months, so it may not take more than 6 months to complete all communities. The census must be completed (i.e., "locked") at the household level before treatment can be given.

6.2. Random Census Verification
A random re-census of households will be conducted at each study visit by supervisors, additional census team members, or local community monitors, as appropriate for the study site. Verification will be performed at the household level, with a minimum of 200 households being resurveyed during the 5 study

visits per country (approximately 40 per visit). Each team must have at least one household census verified at each study visit.

Households will be selected using a different mechanism than the mechanism used by the original census. The primary method for selecting random households will be from aerial visualization (Google maps, AfriPop, etc.) If aerial visualization is not possible in an area, then another method for obtaining households can be used, such as a random walk.

Both census teams will use the same electronic template (i.e., no prior records at MORDOR 0, and the census records from the prior census at each follow-up visit). We will select a random sample of communities stratified by census team. Once the census has been completed, the trial biostatistician will select the communities for verification.

The trial biostatistician will compare the results of the original census and the re-census to identify any discrepancies. The steering committee will determine any corrective actions once this comparison is made. At the very minimum, the site study coordinator will inform the original census team of the discrepancies and will conduct a refresher training session to minimize data collection errors.

6.3. Verbal Autopsy

Verbal autopsy questionnaires will be completed for all deceased children (aged 1-60 months).

Staff

Verbal autopsy interviews will be conducted by trained staff. Training will focus on conducting sensitive interviews with persons who may still be in mourning; discussion of verbal autopsy questions; reviewing the format of the paper and electronic questionnaires (including skip logic); and demonstration of the verbal autopsy technique on 5 mock deaths. The 5 first verbal autopsies will be observed by the study coordinator to ensure that proper procedures are followed. Each verbal autopsy interviewer will be responsible for a distinct geographic area, and will be responsible for regular contact with the key informant from each community.

Identification of Deaths

Deaths will be identified in 2 ways: from the biannual census, and from the key informant system. The trial biostatistician will provide a list of all deaths to the site study coordinator after each census. This list will include information on the deceased child's name, age, gender, and unique identification number; community name; and father's and mother's names. The study coordinator will

in turn deliver this list to the appropriate verbal autopsy interviewer. The verbal autopsy interviewer will also keep a record of all deaths identified by the key informants. In each case, the key informant will report the community name, child's name, and parents' names, and the deceased child will be located in the census database.

Questionnaire Administration

Each site will choose between the full-length WHO questionnaire (2007 version) or an abbreviated form (which utilizes questions from both WHO 2007 and 2012), translated into the local language. Questionnaires will be administered at the home of the deceased child. The informant will be the deceased child's parent or guardian. If this person is not available, the verbal autopsy interviewer will try to arrange a time to return to interview this person. If the parent or guardian is not present on the third visit, they will complete the questionnaire by interviewing another family member, or as a last resort, a neighbor. All interviews will be completed in the local language. Our goal is to perform each verbal autopsy within 2 months of death. The child's name and unique identification number will be entered on the verbal autopsy record for identification purposes.

Assigning the Cause of Death

As recommended by the WHO, we will use automated methods to assign causes of death based on the verbal autopsy questionnaire, rather than physician review. We will treat all individuals under 4 weeks as one subpopulation, and individuals 1-60 months as a separate subpopulation for determination of cause-specific mortality fractions.

Translation

The verbal autopsy will be translated into the local language by a professional translator and then back-translated into English by a second translator. Discrepancies that are noted in the back-translation will be provided to the original translator, and adjustments made as needed.

At each country site, the questionnaire will be validated and reviewed to ensure that it is culturally appropriate.

6.4. Validating the mortality outcome

6.4.1 Niger

Deaths from the biannual census will be validated from local death registries. In an effort to improve the quality of existing death registries in villages and health facilities, the Study Coordinator will conduct sensitization activities in study communities and CSIs before the study begins. During sensitization, the Study

Coordinator will work closely with local village chiefs, mayors, and health centers to develop a strategy for obtaining more accurate death registers during the study.

CSIs and villages maintain local death registries. Health professionals record deaths that occur in the CSI. The CSI death registries include: name, age, village, cause of death. The village chief also maintains a death registry in the village. The village death registry includes: name, age, village, cause of death.

The Study Coordinator will visit each CSI and record in an Access database all deaths from children aged 0-9 years. We collect a larger age group than that collected in the census since age misclassification may occur either on the census or on the death registry. The Study Coordinator will record all information present in the registry for all children aged 0-9 years, including name and village.

The Data Manager will be responsible for linking the death registry file with the census file, using the name, age, and village of deceased children. We expect that both the census and the registry will miss deaths.

Verbal autopsies will be performed on all deaths picked up by the census and also all deaths from 0-5 year-olds picked up by the death registry. Note that the deaths from the death registry will not be used in the primary outcome.

6.4.2 Malawi

Deaths will be identified in 2 ways: from the biannual census, and from the key informant system. The trial biostatistician will provide a list of all deaths to the site study coordinator after each census. This list will include information on the deceased child's name, age, gender, and unique identification number; the community name; and the father's and mother's names. The study coordinator will in turn deliver this list to the appropriate verbal autopsy interviewer. The verbal autopsy interviewer will also keep a record of all deaths identified by the key informants. In each case, the key informant will report the community name, child's name, and parents' names, and the child will be located in the census database. We will provide phone credit as appropriate for the key informants.

Trained verbal autopsy interviewers will be responsible for a distinct geographic area, and will be responsible for being in regular contact with the key informant from each community. We will aim to perform all verbal autopsies within 2 months of death. The interviewee will be the deceased child's parent or guardian. If this person is not available, the verbal autopsy interviewer will try to arrange a time to return to the home to interview this person. If the parent or guardian is not present on the third visit, they will complete the questionnaire by

interviewing another family member, or as a last resort, a neighbor. All interviews will be completed in Chichewa, Yao or English, as appropriate, on a tablet computer.

6.4.3 Tanzania

Community Monitors will visit a random sample of households identified on the census as having children. They will visit their assigned households each month to monitor for deaths. These records will be kept apart from the data collected by the census teams (at six month intervals). The two lists will be compared and any discrepancies followed up.

Chapter 7: Registering Participants for Specimen Collection

Samples will be collected with reference to age, gender, household, and community, but participant names will not be included in laboratory records to ensure privacy. Samples will thus not be associated with an individual's name, but with a random identification number and/or QR code, masking laboratory personnel and preventing identification of individuals.

At each time point, each child selected for examination/specimen collection will be assigned an identification number for database anonymity.

Prior to clinical examination, the registration worker will greet the participant and his/her guardian, identify the participant, verify his/her age, and assign the next random identification number or QR code from the sticker roll. A custom mobile application will be used to complete the registration process. For children registered for anthropometry, the registration worker will take a photo of the child's face. This photo will serve as a reference for the anthropometry team at the next exam phase.

The registration worker will place a QR code sticker on a colored bracelet and write the child's gender (F or M) and age on the bracelet. The registration worker will then put the bracelet on the child's right hand and scan the QR code.

Color bracelet	Age	Examinations
Green	1 – 60 months	Anthropometry only
Yellow	1 – 60 months	Anthro, blood, swabs
Pink	7 – 12 years	Blood and NP swab only

After registration, the child and his/her guardian will be directed to the appropriate examination stations.

Protection of Examiner and Study Participant

Prior to examinations at the blood station and the swab station, the examiner and tuber must be gloved. The examiner will put latex gloves on both of his/her hands prior to touching the participant and a new pair of gloves will be used for each participant in order to avoid transmitting infection between participants. Purell® Instant Hand Sanitizer will be available for hand sanitization when needed.

Chapter 8: Blood samples

We will collect:

1) Thick and thin smears, assessed for malaria parasitemia and gametocytemia by trained local microbiologists,
2) Microcuvettes, analyzed for hemoglobin in the field using a HemoCue analyzer (HemoCue AB, Ängelholm, Sweden), and
3) Dried blood spots, collected on FTA Elute cards (Whatman, Kent, UK; or appropriate substitution) and sent for laboratory testing for malaria using a nested PCR assay[40] and/or TropBio cards (Tropbio Pty Ltd, QLD, Australia) and sent to the CDC for serological testing.

The order of events at the blood collection station is: 1) finger prick; 2) blood spots on filter paper; 3) hemoglobin test; 4) thin and thick smears for malaria.

The assistant will enter data (e.g. scan QR code of child and his/her specimens) using a custom mobile application on a tablet computer.

It is important to handle all blood specimens with care to minimize risk of infection

Wear gloves. New gloves must be worn for each child.
Clean spills. In the event of a blood spill or splash, clean immediately with approved disinfectant (10% bleach or chlorhexidine solution) and wipe with absorbent material.
Disposal of sharps. All lancets must be disposed of properly in sharps containers.
No food. Food and drink are not allowed at the blood collection station.

8.1. Fingerstick

Inform the mother that her child's finger will be pricked to obtain blood to test for malaria and anemia. Describe the finger prick procedure, reassure her, and

answer all questions. The blood specimen should be collected as described below to minimize the discomfort of the child and to ensure sufficient blood volume collection.

A finger stick of capillary blood will be collected for thin and thick blood smears to assess for malaria, hemoglobin testing, and dried blood spots to be stored for later testing. Blood will be collected by a gloved health worker using aseptic technique. Gloves will be changed between each participant. The fingerprick or heelstick site will be disinfected using a 70% isopropyl alcohol swab.

Fingerstick procedure:

1. Prepare the disposable lancet. Use a NEW disposable lancet for each child. **Do not** re-use lancets!
2. The recorder will scan the child's QR code, and place random number stickers on the TropBio filter paper and the (right edge of the) slide.
3. Position the child for the finger stick. Make sure that the child's right hand is warm and relaxed. Hold the child's thumb, middle, or ring finger on his/her right hand (from the top of the knuckle to the tip of the finger) between your left thumb and finger and disinfect in small outward circles with an individually packaged alcohol wipe.
4. After the alcohol dries, use the thumb to lightly press the child's thumb or finger from the top of the knuckle towards the fingertip to stimulate blood flow towards the sampling point (puncture site). For the best blood flow and least pain, prick the side of the thumb/fingertip, not the center. While applying light pressure towards the thumb/fingertip, hold the lancing device in your hand and prick the thumb/finger. If the finger prick is performed properly, a single prick should be sufficient to collect the required amount of blood.
5. Allow the blood to ooze out. Wipe away the first 2 or 3 drops of blood with gauze. If necessary, re-apply light to moderate pressure towards the thumb/fingertip (approximately 1 cm behind the site of the finger prick) until another drop of blood appears.
   ***Note:* Do not** squeeze forcefully. Avoid "milking" as it may dilute the blood with tissue plasma.

8.2. Dried Blood Spot for Malaria

Collecting the FTA Elute filter paper sample:

1. Label the filter paper with a random number sticker.
2. Place 2-4 large drops of blood directly from the thumb or finger onto the large circle on the filter paper (if it is difficult to obtain 4 drops of blood, it is sufficient to collect 2 drops of blood).

3. Leave the filter paper to air dry for a few minutes, then place the sample into a small plastic bag along with a desiccant packet.
4. Leave the bag open for a few minutes more, and when the blood is **completely** dry, roll down the top of the bag and close with a piece of masking tape.
5. Store the filter paper samples (in small plastic bags) in a larger Ziploc bag. Keep all filter paper samples in a safe, dry place at room temperature.
6. Blood spots will be stored at room temperature in a locked cabinet in the study coordinators' office.

Collecting the TroBio filter paper sample:

1. Label the filter paper with a random number sticker.
2. Grip the filter paper on the side without small circles. Place a droplet of blood directly from the thumb or finger onto five of the six circles, leaving one blank. Be sure to fill each circle completely.
3. Carefully slide the filter paper onto a pencil to air dry for at least an hour. There should be about 1 cm in between each sample. Secure the pencil into a Styrofoam surface in a cardboard box.
4. When the filter paper is dry, place each sample into a small zip plastic bag (individually). Place the small bags into a larger Ziploc bag with five desiccant packets.
5. Ensure the large Ziploc bag is sealed tightly, as moisture will damage the samples. Transport these filter paper samples to a freezer.

8.3. Hemoglobin Test

A portable spectrophotometer (HemoCue, Anglom, Sweden) will be used for hemoglobin testing.

Set up HemoCue Analyzer

1) Remove the HemoCue analyzer from the case. If a small battery symbol appears on the top right side of the display, the batteries are low. The HemoCue will still give accurate results, but it is strongly recommended to replace the batteries as soon as possible.
2) Pull the cuvette holder out to the loading position. Press and hold the left button until the display is activated (ALL symbols appear on display). The display will show the version number of the program, an hour-glass symbol and "Hb." At this time, it will perform an automatic SELFTEST to verify the performance of the device. After 10 seconds, the display will show three flashing dashes and the HemoCue symbol. This means that the HemoCue has passed the SELFTEST and is ready for use. If the SELFTEST fails, an error code will be displayed.

Collecting a blood sample for hemoglobin (HemoCue):

1. Remove a cuvette from the container. Reseal the container immediately. (The recorder can help the examiner with this.)
2. When the blood drop is large enough, fill the microcuvette in one continuous process. **Do not refill!** If there is not enough blood to fill the microcuvette, you must start again with a new microcuvette. Wipe any excess blood from the sides of the microcuvette with clean gauze or a paper towel, but be careful to avoid touching the open end of the microcuvette so blood is not removed.
3. Look for any air bubbles in the filled microcuvette. If air bubbles are present, discard the microcuvette and obtain a new drop of blood using a new microcuvette. (Small bubbles around the edge of the microcuvette can be ignored.)
4. Place the filled microcuvette in the cuvette holder. Gently slide the cuvette holder to the measuring position to be analyzed immediately. (This **must** be performed within 10 minutes after filling the microcuvette.)
5. After 15 – 60 seconds, the hemoglobin value will be displayed. The examiner should read the hemoglobin value aloud so the recorder can enter it into the tablet computer. The value will remain on display as long as the cuvette holder is in the measuring position. The analyzer will turn off automatically after 5 minutes.
   Note: For children 6 months to 5 years, if the hemoglobin is <11.0 g/dL, the child is anemic. For children 5 – 11 years, if the hemoglobin is <11.5 g/dL, the child is anemic (WHO/UNICEF/UNU, 1997). If a child is found to be severely anemic, the examiner must refer him/her to the nearest health center for treatment.
6. Carefully dispose of the used microcuvette in the sharps container.
7. **At the end of the day:** Turn off the HemoCue analyzer. Press and hold the left button until the display reads OFF. The display should be blank.

Cleaning the HemoCue Analyzer

1) To clean, pull the cuvette holder to the loading position.
2) Carefully press the small catch (upper right corner of the cuvette holder). Continue to press the catch and carefully rotate the cuvette holder as far left as possible, and then carefully pull the cuvette holder out of the analyzer.
3) Clean the cuvette holder with alcohol or a mild detergent. Push a clean cotton tipped swab moistened with alcohol (without additive) into the opening of the cuvette holder and move from side to side 5 – 10 times. If the swab is dirty, repeat with a new clean swab until the cuvette holder is clean. A dirty cuvette holder may cause the HemoCue analyzer to display an error code.
4) After 15 minutes (or less time, depending on the climate), you may replace the cuvette holder and use the analyzer. The cuvette holder must be completely dry before you replace it.
5) Put the Hemocue analyzer back into its case.

8.4. Thick and Thin Smears for Malaria

1. Label the slide with a random number sticker.
2. For the thick blood smear:
    a. Place a drop of blood in the center (1 cm from the edge of the slide) of a clean, dust-free, and grease-free slide.
    b. Spread the drop of blood evenly with a disposable wooden applicator or with another clean slide into a circle with a diameter of 1 cm.
    c. The blood smear should be about 1cm away from the edge of the slide. The correct thickness of a thick blood smear is one through which newsprint is barely visible when the blood is still wet.
3. For the thin blood smear:
    a. Place a smaller drop of blood on the slide.
    b. Using another slide angled at 45°, create a feathered edge before reaching the other end of the slide.
4. Allow the blood smears to air dry flat. Do not heat the slides, as this will damage the parasites. Be sure to protect the slide from dust and insects. Do not refrigerate slides, as this may cause the smears to detach from the slide during the staining procedure.
5. When dry, place the thick and thin blood smears into the slide box.
6. Smears will be transported at room temperature each day to a diagnostic facility near the study area.
7. Within 24 hours of thick and thin blood smear collection, the smears will be stained with 2% Giemsa stain for 30 minutes. (The thin smear will be fixed by submerging it in 100% methanol for 30 seconds and then let it air dry for 1-2 minutes prior to the Giemsa stain.)
8. Parasite density will be measured by a masked reader using a microscope at the diagnostic facility.

Smears will be stored at room temperature in the study coordinators' office.

A rapid diagnostic test (RDT) could be substituted for thick smears if approved by the Steering Committee.

8.5. Materials for Blood Collection

<u>Fingerprick</u>
Gloves
Disposable lancets
Alcohol wipes
Cotton balls
Gauze

10% household bleach or 4% chlorhexidine solution to clean spills
Absorbent material for spills
Sharps container

Dried Blood Spots
FTA Elute cards
Small zip plastic bags
Desiccant packs
Masking tape
Large Ziploc bags (handful)
TropBio circular cards
Small zip plastic bags
Desiccant packs
Large Ziploc bags (handful)
Materials for drying apparatus: 12 sharpened pencils, Styrofoam, empty cardboard box

Hemoglobin Test
HemoCue machine
Extra set of AA batteries
Cuvettes
Q-tips (handful)

Thick and Thin Blood Smears
Glass slides
Slide box

Rolls of random number stickers

Google Nexus 7
External battery pack

Chapter 9: Specimen Collection for Resistance Testing

9.1. Population

We will collect nasopharyngeal and stool samples on a random set of 40 children aged 1-60 months from each of the communities in the Morbidity study. Children will be selected from the current study census. The swabbing visits will occur after the census but **before** treatment. The list of children will be provided to the site study coordinator before the swabbing visit.

At each swabbing visit, the Registration worker will register the child as described in Chapter 7. S/he will put a label with a QR code on the child's bracelet with his/her age and sex. At the swab station, the tuber will use a custom-mobile application to scan the child's QR code and QR codes affixed to all specimens for that child. These QR codes are each linked to a random number and this system will link the specimens with the child in the database.

9.2 Nasopharyngeal Swabs

Nasopharyngeal swabs will be stored in STGG media, and standard microbiologic techniques will be used to isolate *S. pneumoniae* and test for resistance to azithromycin, penicillin, and clindamycin. Resistant isolates will be assessed for the most common genetic resistant determinants (*ermB* and *mefA*) using a PCR-based assay.[41] Serotype will be assessed using a nested PCR reaction for the most common serotypes, followed by the Quellung reaction for any untyped isolates.[42]

The examiner will:

1. Place a pediatric flocked swab with a nylon tip through the right nostril and down the nasopharynx of each participant. Note that if the swab is not perpendicular to the frontal plane of the face, it is likely not in the inferior turbinate.
2. Once you reach the nasopharynx, rotate the swab 180° as you remove the swab from the nose.
3. Place the swab in a tube containing 1.0 mL of STGG (skim milk, tryptone, glucose, and glycerin) media, cut the handle off using sterile scissors, and close the cap of the STGG tube with the swab immersed.
4. The nasopharyngeal swab samples in STGG will initially be stored in the field at 4°C using an insulated storage bag with Fisher brand ice gel packs, and then transferred to -20°C.
5. The scissors used to cut calcium alginate swabs will be sterilized with alcohol pads or cleaned with bleach wipes between participants.

Do not attempt to collect the NP swab if you are not successful after three attempts.

9.2.1 Materials for Swab Collection for Resistance Testing

Swabs

NP specimens will be collected using sterile, individually-wrapped pediatric flocked swabs with a plastic swab shaft (manufactured by Copan). Nasal sites will be swabbed with a sterile, Dacron polyester-tipped swab with a plastic shaft (manufactured by Fisherbrand).

Sample Tubes
All field samples for DNA testing will be collected into sterile 2.0ml microcentrifuge tubes, manufactured by Sarstedt®.

Cooler Bags with Frozen Ice Packs
Insulated cooler bags will be used to carry samples to and from the field. In addition, frozen gel ice packs designed to thaw slowly will be used to maintain the temperature in the cooler bags during transport.

-20°C Freezer
A standard -20°C freezer located at the local health center will be used strictly for the storage and freezing of ice packs and samples. This freezer is kept in a locked room on the grounds of the Health Center, which is under 24-hour security guard supervision.

9.2.2 Protocol for Tubing and Handling of Samples

The tubing and handling protocol must be carefully followed in order to prevent contamination and ensure the safe transport of the samples back to the local microbiology laboratory and/or to the US for processing. The person in charge of labeling, tubing, arranging, and handling the samples needs to perform this task in the most orderly and attentive manner.

1. Both hands of the tuber should be gloved at all times. The tuber's gloves only need to be changed when any potential contamination of the gloves occurs. The tuber opens the capped, hinged lid of a microcentrifuge tube, which has been labeled with the participant's random identification number.
2. The swab is inserted by the examiner into the microcentrifuge tube held by the tuber. The swab shaft should only be inserted until the swab head is fully in the tube. The tuber will cut the swab shaft with sterile scissors.
3. The tuber should screw the cap of the microcentrifuge tube tightly and place it in the sample collection box, located in the cooler bag filled with frozen ice packs. The flap of the cooler bag should be closed between each patient. The cooler bag should be in as cool a place as possible in the field, in a shaded area out of the sun.
4. Upon returning from the field each day, the samples will be immediately taken to a local health center and stored in a commercial -20°C freezer, reserved solely for storage of specimens and ice packs. All samples will be in sample boxes, labeled with the village name for easy future identification.

9.3 Stool Samples

All study sites will collect either stool samples or rectal swabs for resistance testing, but methodology will depend upon the type of samples collected and consist of either culturing/microbiological testing or nucleic acid based testing.

Rectal Swabs for Culturing/Microbiological Testing

Rectal swabs will be collected and placed into Amies transport media. We will use standard microbiologic techniques to isolate *Escherichia coli*. Resistance to azithromycin, ampicillin, and co-trimoxazole will be determined. Isolates can be further classified into commensal and diarrheagenic subtypes using a multiplex PCR assay.[43]

Stool Samples for Nucleic Acid Based Testing

Stool specimens will be used to look for the presence of E. coli and macrolide resistant determinants typically associated with resistant strains of E. coli, using a resistome approach. This will be carried out by isolating DNA from stool specimens and detecting the presence of E. coli as well as genes associated with antibiotic resistance (i.e. *erm, mef, and mph* genes) via PCR assays.[45] Other possible experiments may eventually include using PCR to detect the presence of toxins or virulence factors (i.e. *eae, stx, bfp-A,VT-1, VT-2*), which are linked to diarrheagenic E. coli strains,[46-47] or exploring the complete microbiome of the stool specimens.

9.3.1 Stool Specimen Collection

Rectal Swab Collection for Culturing/Microbiological Testing

The test will require that the child's parent and examiners work together to obtain a good sample. Is it important to describe the test to the parent so that they can best assist with keeping the child still during the procedure, if necessary.

In place of stool specimens, rectal swabs can be collected in the following way:

1. Give the mother or guardian a baby wipe to wipe the child's bottom prior to sample collection.
2. Put on a clean pair of gloves.
3. Partially open the fecal swab package and remove the top section of the collection vial (this can be discarded).
4. Position the child:
    - Lie the child on his/her back, hold legs in the air (it is useful to have assistance).
    - Or have the child lay on his/her stomach across the mother/guardian's lap

5. Remove the swab from the package. Take care that the cotton tip is not touched. If it is touched, throw the swab away and begin with a new one.
6. Insert the tip of the swab into the child's anus only as far as needed to contact fecal material (1-3cm) and rotate 180 degrees. The tip should be a brownish color when removed.
7. Place swab into the preservative in the Norgen Stool Nucleic Acid Collection Tube. Make sure the swab is fully submerged in the liquid preservative and then break the swab off using the pre-scored breaking point.
8. Screw the cap back on the tube and make sure that it's tightened. Wrap the area where the cap meets the tube with Parafilm to ensure that the sample will not leak, and then place the tube into the appropriate sample box.
    - If the swab cannot be broken off while the tip is fully submerged in the liquid, try twirling the swab in the liquid first (to release the contents of the sample into the preservative) before breaking it off. Avoid rubbing the sample on the tip of the swab off on the side of the tube where there is no liquid.
9. Place a random number label on the collection tube.
10. Place the tube the rectal swab container.
11. **Swab storage for Genetic analysis:** Store samples at room temperature. According to the manufacturer, the preservative in the tube will preserve DNA for 5 months at room temperature (7 days for RNA), and thereafter can be frozen (-20°C or -80°C) for long-term storage.

Stool Specimen Collection for Nucleic Acid Based Testing

For the study participant/parent of the child:

1. Collect the initial stool specimen on a piece of plastic.
2. Transfer a few heaping spoonfuls of the fresh stool into the smaller, 4 oz disposable plastic container that has a locking lid, using the spoon provided. Return this to the trained field worker.

For the trained field worker:

1. Wearing fresh gloves, carefully place a portion of the stool sample from the disposable 4 oz plastic container into a labeled Norgen Stool Nucleic Acid Collection and Transport Tube (15 ml collection tube that contains preservative), using the small spatula that is attached to the tube's cap. Fill up to the line as indicated by the tube.[44] Make sure to spoon the stool into the tube without touching the rim or outside of the tube to avoid any contamination.
2. Once the stool sample has been added, place the cap tightly back onto the tube.

3. Mix gently until the stool is well submerged under the preservative. Do not shake the tube up and down, just gently swirl.
4. Wrap the lid of the tube with a piece of Parafilm to seal it.
5. Place the tube into the storage box and store at room temperature.
6. Once the final stool sample has been collected in the Norgen Stool Nucleic Acid Collection and Transport Tube, wrap up the initial stool sample in the large receptacle container using the plastic lining and properly dispose of it. Also, dispose of the stool sample in the 4 oz plastic container and the spoon that was used.

9.3.2 Materials for Stool Specimen Collection

Rectal Swab Collection for Culturing/Microbiological Testing
Swab
An individually-wrapped Copan flocked swab with a plastic shaft will be used to collect the rectal swab and then placed into a Stool Nucleic Acid Collection and Transport Tube containing Norgen Stool Preservative.

Sample Tube with Amies Transport Medium
The specimen will be in a sterile Stool Nucleic Acid Collection and Transport Tube containing Norgen Stool Preservative with a cap that will be tightened firmly.

Stool Specimen Collection for Nucleic Acid Based Testing
Plastic Lining
Each participant will be given a piece of plastic that will be placed on the ground to collect the initial stool sample.

Spoon
Wooden medical spoon used to transfer a portion of the initial stool specimen to the small plastic container, which will be brought to the trained field worker by the parent.

Small Plastic Container
4 oz disposable plastic container with locking lid used to transport a portion of the initial stool specimen to the trained field worker.

Norgen Sample Tube with Preservative
A portion of the initial stool specimen will be transferred to a sterile Norgen Stool Nucleic Acid Collection and Transport Tube (15ml) containing preservative, with a small spatula attached to the tube's cap.

9.4 Quality Control Measures for Specimen Collection

Negative Field Controls
Negative field control swabs for NP and stool will be taken in each community to assess for contamination: one control swab each (NP and stool) are taken before specimen collection begins in a community; and another (NP and stool) upon completion of specimen collection.

1. For each negative field control, the examiner will open a new swab as described above.
2. Wave the swab in the air, without making contact with anyone/anything.
3. Tube the swab in media, as described above.

Duplicate swabs
Duplicate NP swabs and rectal swabs/stool specimen will be collected from two children per community.

Chapter 10: Cost-Effectiveness

10.1. Costs

We will record the costs of distributions in a random subset of 12 villages at MORDOR 6 ***or*** 12 and MORDOR 24. We will assume a societal perspective. Programmatic costs will be included, such as: azithromycin, antibiotic distributors, supervisors, drivers, fuel, and the time of community volunteers, and the costs of azithromycin will be estimated using the market price for generic formulations. See appendix for the study forms to be used. Collection of certain costs will be incorporated into the electronic data capture system, including the duration of time that the antibiotic distributors worked, the number of antibiotic distributors per team, the number of doses of azithromycin/placebo given, and the number of doses of azithromycin/placebo wasted. Other costs will be collected on paper forms from the appropriate parties: the drivers will complete paper forms describing costs of fuel and automobile maintenance, and the study coordinator will complete paper forms describing the costs of consumables purchased for the study, as well as the overhead costs of maintaining the mass drug administration office.

10.2 Effectiveness

To estimate effectiveness, we will estimate the childhood mortality rate differently from the primary outcome of the study, in that we will account for all person-time in the community for children aged 1-60 months. Children who are born or move into the study area after the initial census will have their person-time counted in the mortality outcome, regardless of whether they are

enumerated at the previous census. Similarly, we will count all childhood deaths in the study area, even if the child never appears on the census. The effectiveness outcome will therefore include children who are born and die within an inter-census interval. Estimating the effectiveness will require several aspects from the census data collection:

- **Pregnancies.** At each census, we will ask whether any household members are pregnant, and at the follow-up census, inquire about the status of the fetus, recording the date of births and deaths.
- **Dates of Births, Deaths, and Migrations.** These dates will be obtained in 2 ways. First, all households will be asked whether any children were born since the previous census, including any infants who already have died, and dates will be recorded. Census workers will also document the date that new households moved into the community, and will ask whether any children were born or have died since making the move. If a child is discovered to be deceased but did not appear on any previous census, the census worker will ask the next of kin about the birthdate, and for how long the child lived in the community being censused. Second, the key informant system will record dates of all births, deaths, and migrations among children aged 1-60 months, and will report these data to the verbal autopsy interviewer. The verbal autopsy interviewer will enter these data into a separate section of the electronic data capture system.

Chapter 11: Training

11.1 Standardization

The three collaborating institutions – UCSF, JHU, and LSHTM – will work together prior to MORDOR 0 to standardize all study procedures. We will review the format, general logistics, and procedures for the house-to-house census. The importance of capturing the vital status of every individual in the study area, including individuals not on the previous census (i.e., new births, deaths and migrations) will be stressed. The importance of capturing those individuals who were born and died in the time period between two censuses will also be highlighted. Census workers who have successfully completed the training will be certified, although certification can be revoked on subsequent quality control checks. Ongoing training activities (before each biannual census) should consist of didactic classroom instruction and mock census activities, followed by in-field training, reviewing the use of the electronic data capture, including charging devices and troubleshooting technical problems.

11.2 Pilot Studies

Prior to MORDOR 0, non-study communities at each study site will be randomly selected as "pilot" villages from the same pool as the mortality and morbidity

communities. The research team will conduct all of the study procedures as described in the mortality study and morbidity study (census, sample collection, and treatment) in these pilot villages for training purposes. Similar to the morbidity and resistance communities, samples will be collected from 40 children, aged 1 – 60 months and 40 children, aged 7 – 12 years. For morbidity, blood samples (thick smears, microcuvettes, dried blood spots), conjunctival swabs, and anthropometric measurements (height, weight, MUAC) will be collected. For resistance, nasopharyngeal, nares swabs, and stool specimens will be collected. Consent will be obtained from the village chiefs and the parents/ guardians of each child prior to sample collection. Everyone in pilot communities will receive one dose of oral azithromycin after sample collection. If the results of the trial are positive, everyone in these villages will also be treated with mass oral azithromycin at the end of the study.

Chapter 12: Sample Organization, Transport, and Storage

12.1 De-identification

All specimens will be labeled in the field with a random identification number linked to the census in the electronic data capture system, but to facilitate masking, only the DCC will have access to the key linking the ID with census information. Age, gender, and community of residence will be available for each specimen, but names will be kept confidential. Therefore, all specimens will be de-identified.

12.2 Specimen Transport

After sample collection, samples from the field will be transported to a local health facility or research center at each study site.

During any international specimen transport, the temperature of the shipper boxes will be documented by a temperature recording device.

Blood Samples

Blood smears (thin and thick) will be transported at room temperature to a local laboratory (depending on capacity at each study site). FTA Elute cards will be transported at room temperature and stored at the health clinic before being transported for processing. TropBio cards will be transferred on ice to the health center and stored at -20°C prior to shipment.

Swabs
Conjunctival swabs and swabs in STGG media will be initially stored in the field at -4°C using a closed, insulated container until arrival at a securely locked freezer at -20°C.

Clinic-based Swabs (non-core)
Swabs will be stored in the -20°C freezer at the health clinic, and then transported for processing .

Stool Samples
Rectal Swabs for Culturing/Microbiological Testing
Rectal swabs preserved in Amies transport medium should be refrigerated until processed. If specimens will be kept more than 2 to 3 days before being cultured, it is preferable to freeze them immediately at -70°C. It may be possible to recover pathogens from refrigerated specimens up to 7 days after collection; however, the yield decreases after the first 1 or 2 days. Frozen specimens should be transported on dry ice.

Stool Samples for Nucleic Acid Based Testing
Specimens preserved in Norgen Stool Nucleic Acid Collection and Transport Tubes can be left at room temperature for 7 days (if preserving RNA) or up to 5 months (if preserving DNA). Specimens can also be transported in the preservative at room temperature. If the samples will be kept for long term storage, they can be placed in a -20°C or -80°C freezer.

12.3 Specimen Storage
Sample storage will occur in two stages: short-term and long-term

12.3.1 Short-term Sample Storage
Samples will be labeled with study ID only and are unidentifiable without access to the study database. Lab capacity and sample export regulations vary between study sites. If there is capacity to perform sample processing in a quality manner (as dictated by IRBs in Tanzania and Malawi), samples will be processed in-country. Otherwise, samples will be shipped to UCSF, JHU, or LSHTM to process core samples and any secondary processing.

12.3.2 Long-term Sample Storage
Depending on each study site's sample export regulations, allowable de-identified samples from Malawi, Niger, and Tanzania will be shipped to UCSF for longer-term storage at the UCSF Oyster Point Facility, which is designed particularly for secure long-term (5 years) storage of biological specimens, at -80°C for future analyses by investigators and other interested parties.

12.4 Catalog Specimens

We will create a list of study data and specimens, including the age, gender, village identification number, treatment assignment, whether treatment was received, vaccination record, and symptom questionnaire. We will also list the date of collection and transport, and the storage conditions while in the field and while banked at UCSF. This will facilitate identification of specimens for future analyses.

Chapter 13: Microbiology and Molecular Laboratory Procedures

Participant names will not be included in laboratory records and samples will be de-identified. Therefore, laboratory personnel will be masked, preventing identification of the individuals infected. Lab results will not be available for weeks, if not months. Thus, all individuals will be treated according to their study arm, whether or not their lab tests reveal evidence of infection.

13.1 Storage and Transport Media

13.1.1 Preparation of STGG Transport Medium

Intended Use

STGG was originally used for long term storage of bacteria at -70C. This media has also been used to successfully transport and recover bacteria from specimens collected on swabs.

Formula

	**Ingredients per number of 1 ml vials**							
Ingredients	1	50	100	200	300	400	500	1000
Skim milk powder (Difco, Detroit, Mich.) Cat.232100 500g	.02g	1g	2.0g	4g	6g	8g	10g	20
Tryptone soy broth (Oxoid-CM129 500g) Fisher cmo129	.03g	1.5g	3.0g	6g	9g	12g	15g	30g
Glucose (Difco-Dextrose cat 215530, 500g)	.005g	.25g	.5g	1g	1.5g	2g	2.5g	5g
Glycerol (Difco- cat 228220 500g)	.1ml	5	10ml	20ml	30ml	40ml	50ml	100ml
Distilled water	1ml	50ml	100ml	200ml	300ml	400ml	500ml	1000ml

Procedure

1. Combine four ingredients in flask and swirl to dissolve (about ½ hour). Use glass cylinder to measure Glycerol. Dispense solution in 1.0ml amounts into screw-cap 1.5ml vials (Sarstedt Microtube, Newton, N.C.) Use Sarstedt rack for easy dispensing.
2. Autoclave vials with caps screwed on loosely at 15 lb/in2 and 121 C for 10 minutes.
3. Do not autoclave vials in plastic storage boxes (boxes will not close when cooled). Use old bottom from broken boxes.

4. STGG should appear milky white to light tan in color with a precipitate at the bottom.

Storage and Shelf Life
Cool vials after autoclaving, screw caps tightly and store at -20 or 4C until use. Use vials within 6 months of preparation.

13.1.2 Preparation of Amies Transport Medium
Rectal swabs will be collected and transported in this medium. It is best prepared from ready to use dehydrated powder, available from most suppliers of culture media. It is usually used at a concentration of 2 grams in every 100 ml distilled water. Fisher Scientific has a dry powder that's available and can be used to prepare the media in house.

Procedure
1. Prepare as instructed by the manufacturer. Dispense the well-mixed medium into screw-cap tubes. Sterilize by autoclaving (with caps loosened) at 121°C for 15 minutes.
2. When the medium has cooled, tighten the caps. During cooling, invert the tubes to ensure an even distribution of the charcoal.

Storage and Shelf Life
Prepared Amies medium in tightly screw-capped tubes can be stored in a cool place at room temperature (away from direct light) until use. Refer to the manufacturer's instructions/guidelines for specifics on expiration dates.

13.1.3 Preparation of Nutrient Agar Medium/Slants
For isolate storage and shipping, blood agar base (BAB), tryptone soy agar (TSA), heart infusion agar (HIA) and nutrient agar are examples of good storage media for enteric organisms. Nutrient agar slants will be used to store/ship isolates originating from samples such as the rectal swabs. Nutrient agar medium is best prepared from ready to use dehydrated powder, available from most suppliers of culture media (i.e. Fisher Scientific). In general, nutrient agar is used at a concentration of 2.8 g in every 100 ml distilled water.

Procedure
1. Prepare as instructed by the manufacturer. Dispense the well-mixed, pre-melted agar medium into tubes (anywhere from 3-5 ml, depending on the size of the tube). Sterilize by autoclaving the tubes (with caps loosened) at 121°C for 15 minutes.
2. While the tubes are still hot after autoclaving, place them in a slanted position to provide a short slant and deep butt (2 to 3 cm). Once medium

is cooled and agar is set, caps can be tightened and slants should be ready for use.

*To inoculate, use an inoculating loop to streak the isolate onto the surface of the agar slant. Incubate overnight at 35° to 37°C.

Storage and Shelf Life
Prepared nutrient agar slants can be stored in a cool dark place and may have a shelf life of up to 2 years provided there is no change in the appearance of the medium to suggest contamination or deterioration. Refer to the manufacturer's instructions/guidelines for specifics on expiration dates.

13.2 Methods for Malaria Testing

13.2.1 Blood Smears
Thin and thick blood smears will be read using standard microbiological techniques at local laboratories at each study site.

13.2.2 Dried Blood Spots

Extracting Genomic DNA from Dried Blood Spots

1. Remove FTA card from plastic bag - there should be 5 dried blood spots. Using the Harris Micro-Punch and mat, punch a 3 mm disc out from one of the blood spots. Place the disc into a 1.5 ml microcentrifuge tube. **When processing multiple samples at a time, make sure to follow the cleaning protocol below in between punching samples.*
2. Add 500 ul of sterile water to the tube and vortex 5 times.
3. Use a clean pipette tip to transfer the washed disc to a clean 1.5 ml microcentrifuge tube.
4. Add 30 ul of sterile water to the tube with the disc and place it in a heating block set to 95°C for 30 min. After incubating the disc, pulse vortex the tube 60 times and then centrifuge. Remove the pelleted disc with a pipet tip and discard.
5. Store eluted DNA at -20°C until ready for use. It is best to run PCR as soon as possible after extracting DNA. Use 5 ul of eluted DNA in CytB PCR reactions (50 ul reaction) as outlined below.

Punch Cleaning Protocol

1. In between each sample, swirl the Harris Micro-Punch in a beaker containing a 1:10 bleach solution for a few seconds and then swirl the tip in a beaker containing a 70% ethanol solution. Dry/blot the tip on a clean paper towel and then allow the punch to air dry for a couple of seconds before using. Discard the used paper towel in the biohazard waste.
    a. Blank control - After cleaning the punch, you may want to take a blank punch from a clean FTA Elute card with no sample and use it as a negative control to confirm that there is no cross contamination.
2. Clean the cutting mat with a Mini Hypewipe (bleach wipe) and then wipe down with ethanol solution using a paper towel. Discard bleach wipe and paper towel in the biohazard waste. (You only have to clean the mat every couple of samples, as long as you use different areas of the mat to punch out each sample – for example, you can clean the mat after every 4 samples if the samples are punched out in four different quadrants of the mat).

Amplifying Gene Regions (PCR)

1. Set up first round of PCR with outer primers: Protocol was adapted from Hsiang, et al. & Steenkeste, et al. papers[40,45] and based on guidelines provided for Qiagen TopTaq DNA Polymerase (refer to Qiagen *TopTaq PCR Handbook*).[46]

Gene	Primer	Sequence
**CytB**	Outer Forward	CGGTCGCGTCCGGTAGCGTCTAATGCCTAGACGTATTCCTGATTATCCAG
	Outer Reverse	CGCATCACCTCTGGGCCGCGTGTTTGCTTGGGAGCTGTAATCATAATGTG
	Inner Forward	GAGAATTATGGAGTGGATGGTG
	Inner Reverse	TGGTAATTGACATCCAATCC

TopTaq PCR Buffer (10X)	5 ul
dNTPs (10 mM)	1 ul
Forward Primer (10 uM)	2 ul
Reverse Primer (10 uM)	2 ul
Template DNA	5 ul
10x CoralLoad Dye	5 ul
Taq Polymerase	0.25 ul
Water	29.75 ul
Total Volume:	50 ul

Note: When setting up several PCR reactions, make up a mastermix for each primer set by multiplying each volume by the number of reactions you have (making up 2-3 more reactions than needed). Aliquot out 45 ul of mastermix for each reaction and then add 5 ul of template DNA for each sample.

- Use the following cycling conditions for the Bio-Rad S1000 thermocycler:
  Initial step: 95°C for 5 min
  *Denature: 94°C for 30 sec
  Anneal: 60°C for 90 sec
  Extend: 72°C for 90 sec
  Repeat from * (Denaturing step to extension step) for 40 cycles
  Final step: 72°C for 10 min

2. Set up second round of PCR with inner primers:
   a. Use the same recipe as above, but use the specific set of inner primers instead of the outer primers for each reaction. Also, use 5 ul of amplicon DNA (DNA from first PCR reaction) as your target.
   b. Use the same cycling conditions as above.

Detecting PCR Products
Use gel electrophoresis to detect and verify the size of expected PCR products.

If running a single-comb 2% agarose e-gel (12 samples total, including MW marker):

1. Load each well that you plan to add a sample to with 15 ul of water. Then load 5 ul of each sample that contains your PCR product into the correct wells. Load 20 ul of water into any remaining empty wells.
2. Load 15 ul of water into the well designated for the MW marker, and then add 5 ul of the marker to that well.
3. The single-comb 2% e-gel needs to run for about 30 min.

If running a double-comb 2% agarose e-gel (16 samples total, plus 2 MW markers):

1. Load each well that you plan to add a sample to with 15 ul of water. Then load 5 ul of each sample that contains your PCR product into the correct wells. Load 20 ul of water into any remaining empty wells.

2. Load 5 ul of water into the well designated for the MW marker, and then add 5 ul of the marker to that well.
3. The double-comb 2% e-gel needs to run for about 15 min.

If running a 48 2% agarose e-gel (48 samples, plus 4 MW markers):

1. Load each well that you plan to add a sample to with 10 ul of water. Then load 5 ul of each sample that contains your PCR product into the correct wells. Load 15 ul of water into any remaining empty wells.
2. Load 10 ul of water into the well designated for the MW marker, and then add 5 ul of the marker to that well.
3. The 48 2% e-gel needs to run for about 20 min. on program "EG".

(For e-gel procedures: see technical manual for running the gel on the Mother E-Base – Do not do a pre-run, but remember to set up the program for the E-base and to place the gel in the E-Base before loading the samples in the gel)

Purifying PCR Products of Positive Samples

Use Qiagen QIAquick PCR Purification kit and corresponding protocol, eluting the DNA in 50 ul of water.[47,48]

Procedure

1. **Add 5 volumes of Buffer PB to 1 volume of the PCR sample and mix.** In our case transfer whatever is left of the PCR sample (about 45 ul) to a clean 1.5 ml microcentrifuge tube and add 225 ul of Buffer PB to it.

2. **If pH indicator I has been added to Buffer PB, check that the color of the mixture is yellow.**
   If the color of the mixture is orange or violet, add 10 µl of 3 M sodium acetate, pH 5.0, and mix. The color of the mixture will turn to yellow.

3. **Place a QIAquick spin column in a provided 2 ml collection tube.**

4. **To bind DNA, apply the sample to the QIAquick column and centrifuge for 30–60 s.**

5. **Discard flow-through. Place the QIAquick column back into the same tube.**

6. **To wash, add 0.75 ml Buffer PE to the QIAquick column and centrifuge for 30–60 s.**

7. **Discard flow-through and place the QIAquick column back in the same tube. Centrifuge the column for an additional 1 min.** IMPORTANT: Residual ethanol from Buffer PE will not be completely removed unless the flow-through is discarded before this additional centrifugation.

8. **Place QIAquick column in a clean 1.5 ml microcentrifuge tube.**

9. **To elute DNA, add 50 µl water (pH 7.0–8.5) to the center of the QIAquick membrane and centrifuge the column for 1 min. Alternatively, for increased DNA concentration, add 30 µl water to the center of the QIAquick membrane, let the column stand for 1 min, and then centrifuge.** IMPORTANT: Ensure that the water is dispensed directly onto the QIAquick membrane for complete elution of bound DNA. The average eluate volume is 48 µl from 50 µl elution buffer volume, and 28 µl from 30 µl elution buffer. Elution efficiency is dependent on pH. The maximum elution efficiency is achieved between pH 7.0 and 8.5. When using water, make sure that the pH value is within this range, and store DNA at –20°C as DNA may degrade in the absence of a buffering agent.

Speciation via Sequencing
Sequetech will automatically quantify the amount of DNA that we send them, and, based on their readings, they will adjust the DNA concentration and optimize the sequencing reaction. For a PCR product that is about 800 bp, they only use about 4 ng of DNA for each sequencing reaction. All the primers that they use in their sequencing reactions are at a concentration of 1.6 uM and they only need 2 ul per reaction.

Preparing the samples for Sequetech
Note: Send primers and template DNA (your samples) in separate tubes – if you premix them, then they cannot quantify your DNA accurately and cannot optimize the sequencing reaction. Also, make sure that all tubes are labeled clearly and match the names that you enter on the order form.

1. Aliquot 6 - 10 ul of each of the purified PCR product that you want to have sequenced into a 1.5 ml microcentrifuge tube and label it clearly (this should be enough DNA for them to sequence each sample both ways).
2. Make up each *nested* primer (keep the forward and reverse primer in separate tubes) that corresponds to your PCR product at a concentration of 3 uM. To do this take 3 ul of the 100 uM stock solution and add it to 97 ul of TE (this will give you a total volume of 100 ul). Based on the number of sequencing reactions you are ordering, calculate how much of the 3 uM primer solution you need to aliquot and send.
   *They will need approximately 1.07 ul of the 3 uM primer solution for each reaction, so to be on the safe side, **allocate 2 ul of the 3 uM primer solution for each sequencing reaction**.
   Calculation: amount of primer needed = (# of sequencing reactions using primer) x 2
3. Organize all the aliquoted and labeled tubes in a storage box and then place the storage box in a sample bag.
4. Place the sequencing order online through Sequetech's website http://www.sequetech.com/, making sure to list all your template DNAs' names, primers' names, the concentration that your primers are at (3 uM), and the approximate size of your PCR products. Print out the order form once you have finished and place a copy of it in the bag with your samples.
5. Schedule a pick-up directly from your location (make sure that everything is ready and scheduled before 3 pm).
6. You can check results online within 24 hours.

13.3 Methods for Resistance Testing

13.3.1 Isolation & Identification of Organisms Using Microbiological Techniques

Various organisms from nasal, nasopharyngeal, and rectal swabs will be isolated using the media and conditions in Table 2 and identified using the methods summarized in Table 3 and below. Standard microbiological handbooks should be referenced for more detailed information.

Procedure for Masking Samples

The microbiologist and all lab staff will be masked to the community of origin.

Laboratory Results Reporting

All lab results will be kept in computer files as well as in hard-copy form by the Microbiologist.

Table 2. Isolation of Organisms from Source - Primary Plating Media & Incubating Conditions

Source	Media	Incubation	Organisms Isolated
Nasal Swab	Blood and MSA	35°C<br>5% CO_2	*S. aureus* (core)
Nasopharyngeal Swab	Blood and Strep select plate, chocolate, chocolate with bacitracin	35°C<br>5% CO_2	*S. pneumoniae* (core)<br>*H. influenzae* (non-core)<br>*N. meningitidis* (non-core)
Rectal Swab	Blood and MacConkey	35°C	*E. coli* (core)

Table 3. Identification of Organisms

Organism	Stain	Identification Tests
S. aureus (core)	Gram stain	Catalase and Tube Coagulase or latex agglutination reagent
S. pneumoniae (core)	Gram stain	Catalase, Optochin disc and/or Desoxycholate reagent, alpha-hemolytic on blood agar
H. influenzae (non-core)	Gram stain	Satelliting growth on blood agar, ALA or other identification tests
N. meningitidis (non-core)	Gram stain	Sugars utilization or other identification kit
S. pyogenes (core)	Gram stain	Catalase, beta-hemolytic on blood agar, and Bacitracin disc or PYR test
E. coli (core)	Gram stain	Indole, oxidase, beta-hemolytic on blood agar, PYR and other identification tests as required

Description of Identification Tests:
We will follow the standard quality control methods already in place at the laboratory. For example, lab staff will perform positive and negative growth controls for all media, and positive controls for all stains and identification tests at a pre-determined schedule.

Catalase Test:
A few drops of 3% hydrogen peroxide are added to colonies removed from agar and smeared onto a clean glass slide, and immediately observed for the release of oxygen or "bubbling." Bubbling is interpreted as the presence of catalase. Lack of bubbling is a negative test.

Tube Coagulase:
Coagulase plasma is inoculated with an isolated staphylococcal colony and incubated for up to 24 hours at 37°C. Any degree of clot formation is a positive reaction and no clot is a negative reaction.

Latex Agglutination:
A few staphylococcal colonies are mixed with the latex agglutination reagent and observed for agglutination. A positive reaction is agglutination of the latex particles, while no agglutination is a negative reaction.

Optochin Test:
An optochin disc is placed onto a blood agar subcultured plate that has been inoculated with a pure culture of the test organism. Plates are incubated for 24

hours at 35°C in CO_2. If a zone of inhibition of 14 mm or more is formed, then it's *S. pneumoniae*.

Desoxycholate (10%) Test:
A drop of reagent is added to a well-isolated colony on a blood agar plate. Colonies of *S. pneumoniae* will disintegrate after 30 minutes.

Satelliting Growth on Blood Agar:
Colonies resembling Haemophilus are subcultured to a non-selective blood agar plate and cross streaked with Coagulase negative Staphylococcus. Plates are incubated in CO_2 overnight and examined for satellite growth around Staphylococcus.

ALA Test:

Sugars Utilization Tests:

Indole Test:

Oxidase Test:

PYR Test:

13.3.2 Microbiological Resistance Testing for Swabs
Resistance testing will be performed using the Kirby Bauer disc diffusion assay or E-test strips (to determine MIC's).

Quality Control
E. coli ATCC 25922, *S. pneumoniae* ATCC 49619, *H. influenzae* ATCC 49247, *S. aureus* ATCC 25923 and 29213 are used for quality control testing of antibiotic discs and E-test strips.

Kirby Bauer Assay
A suspension of the isolate is prepared and turbidity adjusted until it reaches the 0.5 McFarland standard. The suspension is used to inoculate the appropriate susceptibility agar plate for confluent growth. The plate is allowed to dry. Antimicrobial disks are placed on the agar plates with sterile forceps or tweezers; disks may not be moved once they have touched the plates. After the disks are placed on the plate, the plates are inverted and incubated at 35°C for 16 to 18 hours. After incubation, the diameter of the zones of complete inhibition (including the diameter of the disk) (Figure 2) are measured and recorded in millimeters. The measurements can be made with a ruler or sliding caliper on the

undersurface of the plate without opening the lid. The zones of growth inhibition will be compared with the zone-size interpretative table and recorded as susceptible, intermediate, or resistant to each drug tested according to CLSI guidelines (see Appendix).

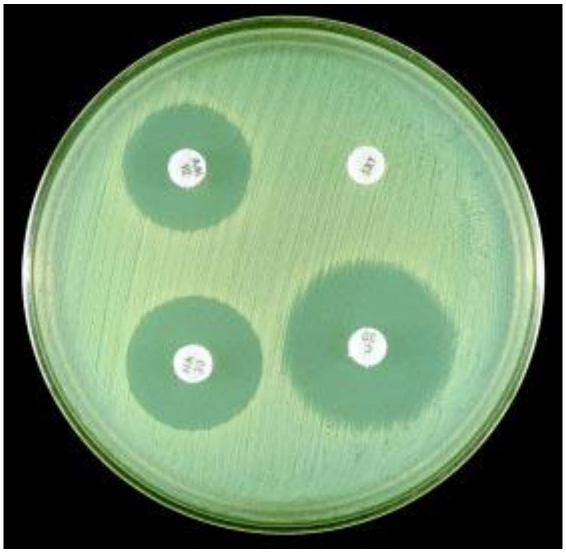

Figure 2. Results of the disk diffusion assay. This *Shigella* isolate is resistant to trimethoprim-sulfamethoxazole and is growing up to the disk (SXT), the zone of which is recorded as 6 mm.

E-test strips.
In addition, azithromycin Minimum Inhibitory Concentrations (MIC) are determined using E-test strips. A suspension of the isolate is prepared and turbidity adjusted until it reaches the 0.5 McFarland standard. The suspension is used to inoculate the appropriate susceptibility agar plate for confluent growth. The plate is allowed to dry. E-test strips are placed on the agar plates with sterile forceps or tweezers; strips may not be moved once they have touched the plates. After the strips are placed on the plate, the plates are inverted and incubated at 35°C for 20 to 24 hours. The MIC is read where the ellipse intersects the MIC scale on the strip. The MIC will be compared with the interpretative table according to CLSI guidelines (see Appendix).

Table 4. Antibiotics Testing Guidelines

Specimen	Organism	Antibiotics Tested
Nasal	*S. aureus*	Azithromycin, cefoxitin
Nasopharyngeal	*S. pneumoniae*<br>*H. influenzae*<br>*N. meningitidis*	Azithromycin, clindamycin, penicillin (Note: *S. pneumoniae* - 1 ug oxacillin used to predict penicillin resistance for Kirby Bauer)
Stool	*E. coli*	Azithromycin, ampicillin, co-trimoxazole

Materials Required for Susceptibility Testing:

- MH w/ 5% sheep blood plates
- MHA plates
- MH broth or sterile 0.85% saline
- Sterile cotton-tipped swabs
- 0.5 MacFarland Standard
- Blood and chocolate plates
- Caliper for reading zones
- Forceps
- Light source
- Vortex
- 35°C ambient air and 35°C CO_2 incubator

13.3.3 Nucleic Acid Based Resistance Testing for Stool Specimens

Stool specimens not cultured will undergo nucleic acid based resistance testing. DNA from the stool samples will be extracted using some type of DNA isolation kit (i.e. Norgen Stool DNA Isolation Kit) and the appropriate corresponding protocol.[49] PCR and gel electrophoresis will then be performed with specific primers to detect genes associated with *E. coli* and antibiotic resistance (specifically macrolide resistance).

Extracting DNA from Stool Procedure

1. **Lysate Preparation**
    a. Mix the sample well to make sure that the stool/preservative mixture is homogenous. Carefully remove the lid from the collection tube and aliquot 200 µl of the stool/preservative liquid and add it to a Bead Tube provided with the kit (it may be easier to use a pipet tip that has been cut to make the opening wider or to use a large orifice pipet tip).
    b. Add **Lysis Solution** to the Bead Tube, so that the final volume is 1 ml (for our purposes, add 800 µl of Lysis Solution). Vortex and centrifuge briefly.
    c. Add 100 µl of **Lysis Additive** and vortex briefly.
    d. Secure the Bead Tube horizontally on a flat-bed vortex pad with tape, or secure the tube in any commercially available bead beater equipment (e.g. Scientific Industries' Disruptor GenieTM). Vortex for 3 minute at maximum speed.
    e. Centrifuge the tube for 2 minutes at **14000 × g (~14,000 RPM)**.
    f. Transfer up to 600 µl of supernatant to a DNAase-free microcentrifuge tube (not provided).
    g. Add 200 µl of **Binding Solution**, mix by inverting the tube a few times, and incubate for 10 minutes on ice.
    h. Spin the lysate for 2 minutes to pellet any cell debris.
    i. Using a pipet, transfer up to 700 µl of supernatant (avoid contacting the pellet with the pipet tip) into a 2 ml DNAase-free microcentrifuge tube (not provided).
    j. Add an equal volume of 70% ethanol (provided by the user) to the lysate collected above (100 µl of ethanol is added to every 100 µl of lysate). Vortex to mix.

2. **Binding to Column**
    a. Assemble a spin column with one of the provided collection tubes.
    b. Apply 600 µL of the clarified lysate with ethanol onto the column and centrifuge for 1 minute at 14000 × g (~14,000 RPM). Discard the flow through and reassemble the spin column with the collection tube. **Note:** Ensure the entire lysate volume has passed through into the collection tube by inspecting the column. If the entire lysate volume has not passed, spin for an additional minute.
    c. Repeat step 2b with the remaining volume of lysate mixture.

3. **Column Wash**
    a. Apply 500 µL of **Wash Solution I** to the column and centrifuge for 1 minute.
    Note: Ensure the entire wash solution has passed through into the collection tube by inspecting the column. If the entire wash volume has not passed, spin for an additional minute.
    b. Discard the flow through and reassemble the spin column with its collection tube.
    c. Apply 500 µL of **Wash Solution II** to the column and centrifuge for 1 minute.
    d. Discard the flow through and reassemble the spin column with its collection tube.
    e. Repeat 3c and 3d.
    f. Spin the column for 2 minutes in order to thoroughly dry the resin. Discard the collection tube.

4. **DNA Elution**
    a. Place the column into a fresh 1.7 mL Elution tube provided with the kit.
    b. Add 50 µL of **Elution Buffer** to the column.
    c. Centrifuge for 2 minutes at 200 x g (~2,000 RPM), followed by a 1 minute spin at 14,000 x g (~14,000 RPM). Note the volume eluted from the column. If the entire volume has not been eluted, spin the column at 14,000 x g (~14,000 RPM) for 1 additional minute.
    d. (Optional): An additional elution may be performed if desired by repeating steps 4b and 4c using 50 µL of Elution Buffer. The total yield can be improved by an additional 20-30% when this second elution is performed.

5. **Storage of DNA**

The purified genomic DNA can be stored at 2-8°C for a few days. For longer term storage, -20°C is recommended.

Amplifying Gene Regions

We will use DNA extracted from stool specimens and published primers to perform PCR reactions for the genes of interest:[50-52]

Purpose	Genes
Detecting E. coli	*malK* (encodes for *malB* promoter) [53]<br><br>*This gene will detect for both *E. coli* and *Shigella* since the two are closely related and are difficult to discriminate from one another.
Detecting Antibiotic Resistance	*mph (A, B)*<br>*erm (A, B, C)*<br>*mef (A)*<br>*ere (A, B)*<br>*msr (A)*

13.4 Methods for *Chlamydia trachomatis* (non-core)

13.4.1 Description of Abbott RealTi*m*e Assay

Swabs will be processed with the Abbott RealTi*m*e assay for *C. trachomatis*, using the automated Abbott *m*2000 System. The RealTi*m*e assay targets the cryptic plasmid of *C. trachomatis*. The assay has been shown to be highly sensitive and specific for the diagnosis of sexually transmitted *C. trachomatis*, with sensitivities exceeding that of the nucleic acid amplification test used in most recent trachoma studies (Roche AMPLICOR).[54,55]

13.4.2 Processing of Samples for PCR

Samples are handled as per Abbott RealTi*m*e sample processing protocol, with the following modifications:

1. Samples are boiled for 10 minutes at 100°C. Boiling of samples is an accepted treatment method to remove substances that may be inhibitory to the PCR amplification process
2. Samples are pooled.

13.4.3 Procedure for Masking Samples

In order to mask the location origin, the control status, and the clinical exam grading of the conjunctival samples collected in the field, the Database Manager will assign an identification number different from the random number assigned to each child. The PCR results will be recorded according to this laboratory

identification number, thus masking the lab until all the samples have been processed. The Database Manager will then link the lab ID number to the random sample number to reveal the test results by village.

13.4.4 Procedure for Pooling Samples

We will increase the efficiency of chlamydial testing by combining swabs from the same age stratum and same community into pools of 5 random swabs for processing. An internal control will be run with each pool to rule out the possibility of PCR inhibitors. Any inhibitory pools will be re-tested, and if still inhibitory, the swabs will be individually re-tested. If PCR from any pool is equivocal, then all swabs from the pool will be tested individually. While samples will necessarily be diluted in this process, this is not thought to affect the sensitivity of the test.[56] We will unpool all positive pools in the 0-5 year age stratum and estimate the community prevalence of chlamydial infection as the proportion of positive swabs. We will estimate the community prevalence of infection in the 6-9 and ≥10 year age strata using maximum likelihood estimation, similar to our previous trials: the number of individual swabs with the maximum likelihood of having resulted in the observed pooled results will be chosen as the estimate for that village.[57,58]

In order to pool the conjunctival samples in the lab, the microbiology lab staff will assign a new pool ID number for every sample, and samples will be stored at -80°C freezer until PCR testing (if not processed that day).

13.4.5 Quality Control

1. A *C. trachomatis*(+) control and a *C. trachomatis*(-) control (targeting 136 base pairs of a pumpkin gene) is included in each test run of the Abbott RealTi*m*e assay.
2. To test the effect of sample processing, a known positive sample is processed and tested in each test run. (This control is helpful when testing large numbers of negative samples.)
3. An internal control intended to identify specimens that contain polymerase inhibitor is run routinely on each sample. The internal control helps identify false negative results.

13.4.6 Quantification

For every 0-5 year old who tests positive for chlamydia, we will also run PCR for the beta actin gene on the same sample, in order to normalize the quantity of chlamydial DNA to the amount of the specimen. Quantitative results from the Abbott system are given in terms of a decision cycle (DC) number. We will generate a ratio of the DC number of the chlamydial DNA to the DC number of the beta actin gene and use the resulting ratio as the chlamydial load.

Chapter 14: Study Medication

Children aged 1-60 months on the current census will be offered weight- or height-based, directly observed, oral suspension (azithromycin or placebo) every 6 months for 2 years (as performed in trachoma programs) at each study site. We will monitor adverse events following mass treatments as described in the adverse events section. The treatment and monitoring schedule for all study arms is shown in Table 1.

14.1 Study Medication Description (from Pfizer, Inc.)

Azithromycin

Zithromax® for oral suspension is supplied in bottles containing azithromycin dehydrate powder equivalent to 1200mg per bottle and the following inactive ingredients: sucrose; tribasic anhydrous sodium phosphate; hydroxypropyl cellulose; xanthan gum; FD&C Red #40; and flavoring including spray dried artificial cherry, crème de vanilla, and banana. After constitution, a 5mL suspension contains 200mg of azithromycin.

14.1 Dosage Information

Azithromycin and placebo will be administered as a single dose, in oral suspension form for children. Dosing will follow the WHO recommendations for treatment of active trachoma:

- Single dose of 20mg/kg in children (up to the maximum adult dose of 1g)
- Height-based dosing of children (this dosing method is supported by the WHO Prevention of Blindness and Deafness group)

Individuals who are allergic to macrolides/azalides will not be treated.

14.2 Medication Procurement/Donation

Azithromycin (Zithromax®) and the placebo have been donated by the Pfizer Corporation. There will be no costs to acquiring the study medication. Pfizer, Inc. will ship azithromycin and placebo directly to the study sites. Representatives of each study site will manage the customs process and transport the medication from the port to storage sites.

14.3 Medication Quality Control

Study medication will be stored at each country site prior to use. The study coordinator and other local staff will regularly check and record the study medication expiration dates. The expiration dates on the medication containers

will be strictly monitored and all expired study medicine will be discarded appropriately.

14.4 Antibiotic Distribution & Monitoring Coverage

After the MORDOR 0 census and monitoring/collection is complete, treatment (azithromycin and placebo) will be administered to all eligible community members per study protocol. Teams will participate in training exercises regarding drug/placebo distribution and recording techniques prior to each treatment cycle.

During mass drug administration, distribution team members will use tablet computers equipped with an electronic data capture system to seek out each eligible child on the census, administer antibiotic or placebo, and record whether or not each person has been treated. The distribution team will document individual reasons for not being treated (e.g. death, temporary absence, permanent migration, refusal of treatment, etc.). Consumption of medication will be directly observed and the dose distributed will be documented in the electronic data capture system.

We will estimate antibiotic coverage from the most recent biannual census records, aiming for treatment of 80% of children. At the end of each treatment round, the UCSF DCC will identify any children who have missed 2 or more consecutive treatments, and forward this information to the site study coordinator. Census teams will discern the reason for missing treatments (including unrecorded death) at the next scheduled census. This system will serve as a quality control mechanism to reduce the number of false negative deaths in the study.

14.5 Adverse Reactions/Side Effects

Azithromycin is generally well-tolerated. The most common side effects of azithromycin are diarrhea, nausea, abdominal pain, and vomiting, each of which may occur in fewer than one in twenty persons who receive azithromycin. Rarer side effects include abnormal liver function tests, allergic reactions, and nervousness. Diarrhea due to *Clostridium difficile* has been reported in rare cases.

During the consent process, the common adverse reactions that may occur will be explained to parents/guardians and they will be advised to communicate adverse events to local program representatives immediately. If, for any reason, the participant needs further care, they will be referred to the nearest health center for examination and treatment.

The trial sites will be masked to outcomes, so the responsibility for monitoring interim analysis will fall on the DSMC. Statistical monitoring is discussed in the Statistical Analysis Plan. The Data Safety and Monitoring Committee (DSMC) will be given authority to discontinue treatments at any time if there is evidence of unexpected harm.

14.6 Adverse Events Systems

Both active and passive monitoring systems for adverse events are in place for this study, and these monitoring activities will specifically include (but will not be limited to) treated 1-6 month olds. We will monitor adverse events following mass treatments actively at each follow-up census and during a house-to-house survey of all 1-6 month olds in a random selection of azithromycin and control communities.

14.6.1 Passive Adverse Events Monitoring

We will implement a passive monitoring system during the treatment phase, by instructing parents to report any adverse events in the two weeks following each mass azithromycin distribution to a local healthcare provider. Children will be referred for follow up care on a case-by-case basis.

14.6.2 Active Adverse Events Monitoring

During Census

We will employ two forms of active monitoring for adverse events. First, during each follow-up census we will ask the guardians of all 7-12 month-old children in all study communities (both mortality and morbidity), whether their children had an adverse event after the previous mass medication distribution. This will allow assessment of adverse events from medication distribution among the individuals aged 1-6 months at the time of the distribution. This information will be incorporated into the electronic data capture system so that this question is asked for any child aged 7-12 months. Note that this method will be subject to recall bias, so we will also perform a house-to-house survey (described below).

Infant Adverse Events Survey

To identify any adverse events associated with mass treatment, the UCSF DCC will randomly select 30 study communities (15 azithromycin and 15 control communities) to participate in an adverse events survey. This survey will be performed by the census workers masked to treatment arm, approximately 2 weeks after a mass medication distribution. During the survey, adverse events will be elicited only for study participants aged 1-6 months at the previous census. A structured questionnaire will be performed to elicit dangerous side effects, followed by an open-ended question. Specifically, we will ask the

primary caregiver about the following symptoms during the time since the previous antibiotic distribution: abdominal pain, vomiting, nausea, diarrhea, dyspepsia, constipation, hemorrhoids or rash. To elicit symptoms of serious infantile hypertrophic pyloric stenosis which has been reported in rare occasions with macrolide use, census workers will be trained to ask about projectile vomiting and feeding problems.

Training
The household survey team will be the same individuals who conducted the census. They will be trained in survey administration methods, including:

1. Obtaining informed consent
2. Accurately selecting the appropriate households to interview
3. Remaining neutral when asking questions (i.e. asking the question exactly as it is written on the paper in a neutral tone of voice, so as not to lead the respondent or introduce bias)

Serious Adverse Events
Any serious adverse events (SAE) will be reported to Pfizer. An **IIR SAE Form** (*Investigator-Initiated Research Serious Adverse Events Form*) will be completed for each event. (See Appendix for form and complete instructions.)

According to Pfizer, an SAE is any adverse event that:

- Results in death
- Is life-threatening (i.e., causes an immediate risk of death)
- Requires inpatient hospitalization or prolongation of existing hospitalization
- Results in persistent or significant disability or incapacity
- Results in a congenital anomaly or birth defect

Or that is considered to be:

- An important medical event

All community residents will be advised to alert a village health worker if they experience, within one week of mass treatment, a serious adverse event (by the preceding definition). The local health worker will report to the on-site study coordinator; who must, within 24 hours, submit a Pfizer **IIR SAE Form** to mordor.sae@gmail.com. TL will review, and forward to Pfizer and/or the Medical Monitor, as appropriate. SAEs must be submitted to Pfizer within 24 hours of receipt from the on-site coordinator.

14.6.3 Adverse Events Data

We will keep records and report all adverse events of azithromycin to the DSMC. We will report both efficacy and side effects of azithromycin separately for the 1-6 month old age group. For any "sudden deaths" believed to be associated with azithromycin treatment, key informants will immediately notify the verbal autopsy interviewer via SMS message or another appropriate form of rapid communication.

14.8 Supply issues

If a study site runs out of a treatment letter, a request should be sent to mordor.tx@gmail.com to request a replacement for the community in question. This is not blanket permission to substitute one letter for another – if there are several communities for which the assigned treatment has run out, a separate request must be made for each community.

The study site coordinator will make the request; TCP will determine the replacement letter, a member of the DCC (TCP, KR, or ZZ) will make the change(s) in the database.

The field team must log out of the MORDOR mobile app and log in again for the changes to take effect on the front end. The replacement treatment letter will then appear in the app.

Chapter 15: Protection of Human Subjects

Before the study begins, each site will obtain formal ethical approval from their respective national ethics committee. In addition, local staff will approach community leaders to describe the study and answer any questions. Study staff will proceed only if local leadership consents to participate. At the individual level, we will obtain verbal consent from a parent or guardian for all study activities with patient contact, including censuses, examinations, and treatments.

Verbal consent is the best way to obtain consent, due to the high illiteracy rates in each study area. Children will be included in the study only following the receipt of consent from a parent or guardian. If, at any time, a parent or guardian elects to withdraw a family member from the study, they will be free to do so. Individuals who withdraw will be offered the same medical treatment outside the study.

Children with wasting, stunting, malaria, or anemia will be referred for appropriate treatment by trained study personnel, at the nearest health center.

15.1 Institutional Review Board Approval

15.1.1. Niger

UCSF Committee on Human Research
UCSF's Committee on Human Research will annually review study protocol for ethical approval.

Emory University Institutional Review Board
The Emory University Institutional Review Board will annually review study protocol for ethical approval.

Comité d'Ethique du Niger
The study protocol will be reviewed and granted ethical approval by the Comité d'Ethique du Niger before any patient-related research activities begin.

15.1.2 Tanzania

Johns Hopkins University Institutional Review Board
The Johns Hopkins University Institutional Review Board will annually review study protocol for ethical approval.

Tanzania National Institute for Medical Research Ethical Review Board
The study protocol will be reviewed and granted ethical approval by the Tanzanian IRB before any patient-related research activities begin.

15.1.3 Malawi

London School of Hygiene & Tropical Medicine Institutional Review Board
The London School of Hygiene & Tropical Medicine Institutional Review Board will annually review study protocol for ethical approval.

Malawi IRB
The study protocol will be reviewed and granted ethical approval by the University of Malawi Blantyre IRB before any patient-related research activities begin.

15.2 Informed Consent

First, the chairman of each village will be asked for permission to include the village in the study. Additionally, the study will be discussed with all adults in the village by team members who speak the local language(s).

Informed consent scripts will be translated into local languages before the study can begin. Consent scripts will then be back-translated by a different party to ensure comprehension. Consent scripts will be submitted and approved by national IRB committees in Malawi, Niger, and Tanzania prior to study implementation. Then they will be read aloud to each study participant (and his/her parent/guardian) by a local team member who is a native speaker of the local language to ensure that they understand the risks and benefits of participating in all study activities. Young adults and children under 18 years of age, who cannot give consent by law, will be included in the study only following the receipt of verbal informed consent from a parent or guardian. If, at any time, a parent or guardian elects to withdraw themselves or a family member from the study, it will be made clear that they will still be eligible for treatment.

15.3 Risks and Benefits of Study Procedures

15.3.1 Verbal Autopsy

As verbal autopsy requires a family member to answer questions about a deceased loved one, he or she might experience emotional stress and grief related to the death of the child. Interviewers will be trained to address these situations appropriately with awareness of the cultural context before they are allowed to conduct these verbal autopsies. If the family member is in need of a mental health intervention, referrals will be made by the interviewer.

15.3.2 Swabbing Procedures

There are minimal risks to the participant who receives nasopharyngeal, and nares swabbing. Participants may experience some temporary discomfort, but the swabbing involves minimal risk. Any adverse effects, such as nose-bleeds, will be treated immediately by the examiners. Other health care will be provided at no cost to the study participant if necessary to address a study-related adverse health event.

15.3.3 Stool Collection

Stool samples have been collected in this setting before, with essentially no risk to participants.

15.3.4 Blood Testing

Blood testing will include a pin prick to the finger or heel. The major risk of this procedure is infection at the puncture site, though using aseptic technique will minimize this occurrence. Individuals in these communities are familiar with this procedure because all children who present at a health center with fever are offered the pinprick for a malaria thick smear.

15.3.5 Anthropometric Measurements

There are minimal risks associated with the measuring board, scale, or MUAC tapes aside from anxiety during the measurements. Examiners will do their best to ensure that the parent/guardian of the child understands the process of assessing anthropometric measurements. The examiners will attempt to minimize discomfort for all study participants before, during, and after the measurements are taken. Children with wasting, stunting, malaria, or anemia will be referred for appropriate treatment at the nearest health center.

Chapter 16: Study Monitoring

The UCSF team will conduct annual monitoring visits to project sites in Malawi and Tanzania. The LSHTM team will conduct annual visits to Niger. Annual site visits will ensure uniform data collection, as well as adequate training and certification.

Chapter 17: Data and Safety Monitoring Committee Charter

This Charter is for the Data Safety and Monitoring Committee (DSMC) for *Mortality Reduction after Oral Azithromycin* (MORDOR): OPP1032340.[59]

The Charter will define the primary responsibilities of the DSMC, its relationship with other trial components, its membership, and the purpose and timing of its meetings. The Charter will also provide the procedures for ensuring confidentiality and communication, statistical monitoring guidelines to be implemented by the DSMC, and an outline of the content of the Open and Closed Reports that will be provided to the DSMC.

17.1 Primary Responsibilities of the DSMC

The DSMC will be responsible for safeguarding the interests of trial participants, assessing the safety and efficacy of the interventions during the trial, and monitoring the overall conduct of the trial. The DSMC will provide recommendations about stopping or continuing the trial. To contribute to the integrity of the trial, the DSMC may also formulate recommendations relating to the selection/recruitment/retention of participants, to protocol-specified regimens, and the procedures for data management and quality control.

The DSMC will be advisory to the trial leadership group, hereafter referred to as the Steering Committee (SC). The SC will be responsible for promptly reviewing the DSMC recommendations and determining, whether to continue or terminate the trial, and to determine whether amendments to the protocol are required. If needed, the DSMC may seek the advice of a content expert outside of the committee.

17.2 DSMC Membership

The DSMC is an independent multidisciplinary group consisting of epidemiologists, biostatisticians, bioethicists, and clinicians that collectively has experience in the management of infectious diseases and in the conduct and monitoring of randomized clinical trials including subsaharan Africa.

Judd Walson, MD, MPH (Chair): pediatrics and global health
Alan Hightower, MS: biostatistics
Latha Rajan, MD, MPH&TM: global health
Emily Anderson, PhD, MPH: bioethics
Wondu Alemayehu, MD, MPH: global health

17.3 Conflicts of Interest

The DSMC membership has been restricted to individuals free of apparent conflicts of interest. The source of these conflicts may be financial, scientific, or regulatory. Thus, neither study investigators nor individuals employed by the sponsor, nor individuals who might have regulatory responsibilities for the trial products, are members of the DSMC.

The DSMC members will disclose to fellow members any consulting agreements or financial interests they have with the sponsor of the trial, with the contract research organizations (CRO) , or with other sponsors having products that are being evaluated or that are competitive with those in the trial. The DSMC will be responsible for deciding whether these consulting agreements or financial interests materially impact their objectivity.

The DSMC members will be responsible for advising fellow members of any changes in any of the membership requirements that occur during the course of the trial. It may be appropriate for DSMC members who develop significant conflicts of interest resign from the DSMC.

DSMC membership is to be for the full duration of the trial. If any members leave the DSMC, the SC, in consultation with the DSMC, will promptly appoint a replacement.

17.4 Timing and Purpose of the DSMC Meetings

Organizational Meeting

The initial meeting of the DSMC will be an Organizational Meeting. This is during the final stages of protocol development and the purpose is to provide advisory review of scientific and ethical issues relating to study design to discuss

the standard operating procedures and to discuss the format and content of the Open and Closed Reports that will be used to present trial results.

The Organizational Meeting will be attended by all DSMC members, lead trial investigators, and the trial biostatistician. The DSMC will be given the drafts of the trial protocol, the Statistical Analysis Plan, the DSMC Charter, and the current version of the case report forms. At subsequent meetings, committee members will receive Open and Closed Data Reports.

Formal Interim Analysis Meetings

One or more 'Formal Interim Analysis' meetings will be held to review data relating to treatment safety and efficacy, and quality of trial conduct. There will be at least two interim decisions to be made by the DSMC, at approximately 12 months and 24 months into the study.

17.5 Procedures to Ensure Confidentiality and Proper Communication

To enhance the integrity and credibility of the trial, procedures will be implemented to ensure the DSMC has access to all emerging information from the trial regarding comparative results of efficacy and safety, aggregated by treatment arm.

Closed Sessions

Sessions involving only DSMC members and, where appropriate, those unmasked trial investigators (on the Data Coordinating Committee) who generate the Closed Reports (called Closed Sessions) will be held to allow discussion of confidential data from the trial, including information about the relative efficacy and safety of interventions.

At a final Closed Session, the DSMC will develop a consensus on its list of recommendations, including that relating to whether the trial should continue.

Open Session

In order for the DSMC to have access to information provided, by study investigators, or members of regulatory authorities, a joint session between these individuals and DSMC members will be held between the Closed Sessions.

Open and Closed Reports

For each DSMC meeting, Open and Closed Reports will be provided. Open Reports, will include data on recruitment and baseline characteristics, pooled data on eligibility violations, and completeness of follow-up and compliance. The study statistician (TCP) will prepare these Open Reports.

Closed reports, available only to those attending the Closed Sessions of the meeting, will include analyses of primary and secondary efficacy endpoints, including subgroup and adjusted analyses, AEs and symptom severity, , and Open Report analyses that are displayed by intervention group. These Closed Reports will be prepared by the study biostatistician.

The Open and Closed Reports should provide information that is accurate, with follow-up that is complete to within two months of the date of the DSMC meeting. The Reports should be provided to DSMC members approximately three days prior to the date of the meeting.

Minutes of the DSMC Meeting
The research team will prepare minutes for the open portion of the meeting, including the DSMC's recommendations.

Recommendations to the Steering Committee (SC)
At each meeting of the DSMC during the trial, the committee will make a recommendation to the Steering Committee to continue or terminate. This recommendation will be based primarily on safety and efficacy considerations and will be guided by statistical monitoring guidelines defined in this Charter.

Recommendations to amend the protocol or conduct of the study made by the DSMC will be considered and accepted or rejected by the SC. The SC will be responsible for deciding whether to continue or to stop the trial based on the DSMC recommendations.

The DSMC will be notified of all changes to the protocol or to study conduct. The DSMC concurrence will be sought on all substantive recommendations or changes to the protocol or study conduct prior to implementation.

The SC may communicate information in the Open Report to the sponsor and may inform them of the DSMC recommended alterations to study conduct or early trial termination in instances in which the SC has reached a final decision agreeing with the recommendation. The SC will maintain confidentiality of all information it receives other than that contained in the Open Reports until after the trial is completed or until a decision for early termination has been made.

17.6 Statistical Monitoring Guidelines
The SC will propose statistical rules for a futility stopping rule (requested by the sponsor) and an efficacy stopping rule at the first DSMC meeting. A decision will be made whether the efficacy stopping rule is appropriate for the relatively short, 2-year study.

17.7 DSMC Contact Information

Table 5: DSMC Contact Information

Wondu Alemayehu<br>Consultant Ophthalmologist<br>Berhan Health<br>PO Box 6307<br>Addis Ababa<br>Ethiopia<br>+251 910 57 48 42<br>walemayehu@berhan-health.org
Emily Anderson<br>Assistant Professor<br>Loyola University Chicago<br>Health Sciences Division<br>2160 S. First Ave.<br>Maywood, IL 60153<br>708.327.9229<br>emanderson@lumc.edu
Allen Hightower<br>Statistician<br>2189 Oakawana Drive<br>Atlanta, GA 30345<br>706.341.0176<br>awh1953@gmail.com
Latha Rajan<br>Clinical Associate Professor<br>1440 Canal Street<br>Suite 2210<br>Department of Tropical Medicine<br>New Orleans, LA 70112<br>504.988.7970<br>lrajan@tulane.edu
Judd Walson, Chair<br>Associate Professor<br>University of Washington<br>Box 359909<br>325 Ninth Avenue<br>Seattle, WA 98104<br>206.744.3695 (office)<br>206.612.4571 (mobile)<br>walson@uw.edu

Chapter 18: Data Collection, Management, and Security

18.1. Scope of Data

Mortality and morbidity data from Malawi, Niger, and Tanzania will be collected in this trial. Mortality data includes: census, mortality, and treatment. Morbidity and resistance data includes the following: census, mortality, treatment, and morbidity assessments. Costing data will also be collected, including the costs of azithromycin, antibiotic distributors, supervisors, drivers, fuel, and time of community volunteers.

Mortality Study

Trained census workers will collect census data on all households in the study sites (name, birthdate, age, gender of all household members) and keep track of births, deaths, and migration of children eligible for treatment. In addition to biannual census updates, trained community health workers and study supervisors will conduct WHO verbal autopsy interviews through the duration of the study to provide information on the cause of death. Trained distribution teams will collect data on treatment status and dose given to all study participants, if treatment is provided apart from the time of census.

Morbidity Study

Census, mortality, and treatment data collection procedures will be identical to the mortality study. In addition, trained health workers will collect data on core morbidity assessments such as blood samples (thick smears and dried blood spots for malaria, microcuvettes for hemoglobin), stool samples, nasal swabs, and nasopharyngeal swabs. Note that for de-identification purposes a random number sticker will be affixed to each specimen collected. In addition, before sample collection, parents or guardians will be asked a standardized series of questions to determine whether the child has had recent fever, cough, or diarrhea. Clinic-based case finding will be conducted at local health clinics, which will involve transcription of health records.

Certain morbidity assessments will be entered into handheld mobile devices at the time of the examination (e.g. hemoglobin, responses to symptom questionnaire), while lab results for thick smears, dried blood spots, nasal, nasopharyngeal, and stool specimens will be entered after confirmation.

18.2 Data Storage, Management, and Security

Data will be recorded electronically using handheld mobile devices with custom-made software applications and uploaded daily onto a secure, password protected, central server. Rapid transfer of electronically captured data will allow

nearly real-time monitoring of activity at the study site. Each study site will have a local data coordinating center within the study area. All handheld devices and data entry coordinating centers will be password protected, and all changes in data will be noted, including the date of the change, and the person who made the change. To ensure the quality of the data, we will conduct training sessions before each biannual census where needed. The central database application will use hard disk encryption and physical protection of the server (which is to be maintained in a locked room accessible only to authorized personnel). The database will be based on mySQL (which supports standard SQL queries). Data will be backed up off site (providing integrity in case of the physical loss of the server). Data will never be deleted from mobile capture devices until at least one offsite backup has been completed. Data security during electronic transfer will be achieved through use of the Advanced Encryption Standard (AES).

References

1. Rajaratnam JK, Marcus JR, Flaxman AD, et al. Neonatal, postneonatal, childhood, and under-5 mortality for 187 countries, 1970-2010: a systematic analysis of progress towards Millennium Development Goal 4. Lancet 2010;375:1988-2008.
2. Porco TC, Gebre T, Ayele B, et al. Effect of mass distribution of azithromycin for trachoma control on overall mortality in Ethiopian children: a randomized trial. JAMA : the journal of the American Medical Association 2009;302:962-8.
3. Shaikh S, Schulze KJ, Kurpad A, et al. Development of bioelectrical impedance analysis-based equations for estimation of body composition in postpartum rural Bangladeshi women. The British journal of nutrition 2012:1-9.
4. Waage J, Banerji R, Campbell O, et al. The Millennium Development Goals: a cross-sectoral analysis and principles for goal setting after 2015 Lancet and London International Development Centre Commission. Lancet 2010;376:991-1023.
5. You D, Wardlaw T, Salama P, Jones G. Levels and trends in under-5 mortality, 1990-2008. Lancet 2010;375:100-3.
6. Bhutta ZA, Chopra M, Axelson H, et al. Countdown to 2015 decade report (2000-10): taking stock of maternal, newborn, and child survival. Lancet 2010;375:2032-44.
7. Black RE, Cousens S, Johnson HL, et al. Global, regional, and national causes of child mortality in 2008: a systematic analysis. Lancet 2010;375:1969-87.
8. Arifeen SE, Hoque DM, Akter T, et al. Effect of the Integrated Management of Childhood Illness strategy on childhood mortality and nutrition in a rural area in Bangladesh: a cluster randomised trial. Lancet 2009;374:393-403.
9. Sazawal S, Black RE. Effect of pneumonia case management on mortality in neonates, infants, and preschool children: a meta-analysis of community-based trials. Lancet Infect Dis 2003;3:547-56.
10. Moonen B, Cohen JM, Snow RW, et al. Operational strategies to achieve and maintain malaria elimination. Lancet 2010;376:1592-603.
11. Solomon A, Zondervan M, Kuper H, Buchan J, Mabey D, Foster A. Trachoma control: a guide for programme managers. Geneva: World Health Organization; 2006.
12. Melese M, Alemayehu W, Lakew T, et al. Comparison of annual and biannual mass antibiotic administration for elimination of infectious trachoma. JAMA 2008;299:778-84.
13. Lietman T, Porco T, Dawson C, Blower S. Global elimination of trachoma: how frequently should we administer mass chemotherapy? Nat Med 1999;5:572-6.
14. Schachter J, West SK, Mabey D, et al. Azithromycin in control of trachoma. Lancet 1999;354:630-5.
15. Bailey RL, Arullendran P, Whittle HC, Mabey DC. Randomised controlled trial of single-dose azithromycin in treatment of trachoma. Lancet 1993;342:453-6.
16. Coles CL, Seidman JC, Levens J, Mkocha H, Munoz B, West S. Association of mass treatment with azithromycin in trachoma-endemic communities with short-term reduced risk of diarrhea in young children. The American journal of tropical medicine and hygiene 2011;85:691-6.

17. Lode H, Borner K, Koeppe P, Schaberg T. Azithromycin--review of key chemical, pharmacokinetic and microbiological features. The Journal of antimicrobial chemotherapy 1996;37 Suppl C:1-8.
18. Miller GY, Algozin, K. A., McNamara, P. E. and Bush, E. J,. Productivity and economic impacts of feed-grade antibiotic use in pork production. J Agr Appl Econ 2003;12:469-82.
19. Fry AM, Jha HC, Lietman TM, et al. Adverse and beneficial secondary effects of mass treatment with azithromycin to eliminate blindness due to trachoma in Nepal. Clin Infect Dis 2002;35:395-402.
20. Sadiq ST, Glasgow KW, Drakeley CJ, et al. Effects of azithromycin on malariometric indices in The Gambia. Lancet 1995;346:881-2.
21. Lopez AD, Mathers CD, Ezzati M, Jamison DT, Murray CJ. Global and regional burden of disease and risk factors, 2001: systematic analysis of population health data. Lancet 2006;367:1747-57.
22. Chidambaram JD, Melese M, Alemayehu W, et al. Mass antibiotic treatment and community protection in trachoma control programs. Clin Infect Dis 2004;39:e95-7.
23. House JI, Ayele B, Porco TC, et al. Assessment of herd protection against trachoma due to repeated mass antibiotic distributions: a cluster-randomised trial. Lancet 2009;373:1111-8.
24. Goossens H, Ferech M, Vander Stichele R, Elseviers M. Outpatient antibiotic use in Europe and association with resistance: a cross-national database study. Lancet 2005;365:579-87.
25. Pihlajamaki M, Kotilainen P, Kaurila T, Klaukka T, Palva E, Huovinen P. Macrolide-resistant Streptococcus pneumoniae and use of antimicrobial agents. Clin Infect Dis 2001;33:483-8.
26. Bronzwaer SL, Cars O, Buchholz U, et al. A European study on the relationship between antimicrobial use and antimicrobial resistance. Emerg Infect Dis 2002;8:278-82.
27. Hotez PJ, Fenwick A, Savioli L, Molyneux DH. Rescuing the bottom billion through control of neglected tropical diseases. Lancet 2009;373:1570-5.
28. Sommer A, Tarwotjo I, Djunaedi E, et al. Impact of vitamin A supplementation on childhood mortality. A randomised controlled community trial. Lancet 1986;1:1169-73.
29. Rahmathullah L, Underwood BA, Thulasiraj RD, et al. Reduced mortality among children in southern India receiving a small weekly dose of vitamin A. N Engl J Med 1990;323:929-35.
30. Vijayaraghavan K, Radhaiah G, Prakasam BS, Sarma KV, Reddy V. Effect of massive dose vitamin A on morbidity and mortality in Indian children. Lancet 1990;336:1342-5.
31. West KP, Jr., Pokhrel RP, Katz J, et al. Efficacy of vitamin A in reducing preschool child mortality in Nepal. Lancet 1991;338:67-71.
32. Vijayaraghavan K, Radhaiah G, Reddy V. Vitamin A supplementation and childhood mortality. Lancet 1992;340:1358-9.
33. Vitamin A supplementation in northern Ghana: effects on clinic attendances, hospital admissions, and child mortality. Ghana VAST Study Team. Lancet 1993;342:7-12.

34. Daulaire NM, Starbuck ES, Houston RM, Church MS, Stukel TA, Pandey MR. Childhood mortality after a high dose of vitamin A in a high risk population. BMJ 1992;304:207-10.
35. Bryce J, Terreri N, Victora CG, et al. Countdown to 2015: tracking intervention coverage for child survival. Lancet 2006;368:1067-76.
36. Jones G, Steketee RW, Black RE, Bhutta ZA, Morris SS. How many child deaths can we prevent this year? Lancet 2003;362:65-71.
37. International Trachoma Initiative: Frequently Asked Questions. (Accessed November 3rd, 2011, at http://trachoma.org/iti-frequently-asked-questions.)
38. Fenwick A, Molyneux D, Nantulya V. Achieving the Millennium Development Goals. Lancet 2005;365:1029-30.
39. Verbal Autopsy Standards: Ascertaining and attributing causes of death. 2013. (Accessed March 6th, 2013, 2013, at http://www.who.int/healthinfo/statistics/verbalautopsystandards/en/.)
40. Hsiang MS, Lin M, Dokomajilar C, et al. PCR-based pooling of dried blood spots for detection of malaria parasites: optimization and application to a cohort of Ugandan children. Journal of clinical microbiology 2010;48:3539-43.
41. Sutcliffe J, Grebe T, Tait-Kamradt A, Wondrack L. Detection of erythromycin-resistant determinants by PCR. Antimicrobial agents and chemotherapy 1996;40:2562-6.
42. Pai R, Gertz RE, Beall B. Sequential multiplex PCR approach for determining capsular serotypes of Streptococcus pneumoniae isolates. Journal of clinical microbiology 2006;44:124-31.
43. Tobias J, Vutukuru SR. Simple and rapid multiplex PCR for identification of the main human diarrheagenic Escherichia coli. Microbiol Res 2011.
44. Norgen Biotek Corporation. Stool Nucleic Acid Collection and Transport Tubes Product Insert (PI45650-1). In; 2013.
45. Steenkeste N, Incardona S, Chy S, et al. Towards high-throughput molecular detection of Plasmodium: new approaches and molecular markers. Malar J 2009;8:86.
46. Qiagen. TopTaq PCR Handbook 2010.
47. Qiagen. QIAquick Spin Handbook 2008.
48. GE Healthcare. Nucleic Acid Sample Preparation for Downstream Analyses Principles and Methods Handbook.23-9.
49. Norgen Biotek Corporation. Stool DNA Isolation Kit Product Insert (PI27600-3) In; 2009.
50. Phuc Nguyen MC, Woerther PL, Bouvet M, Andremont A, Leclercq R, Canu A. Escherichia coli as reservoir for macrolide resistance genes. Emerg Infect Dis 2009;15:1648-50.
51. Nakhjavani FA, Emaneini M, Hosseini H, et al. Molecular analysis of typical and atypical enteropathogenic Escherichia coli (EPEC) isolated from children with diarrhoea. Journal of medical microbiology 2013;62:191-5.
52. Ramotar K, Waldhart B, Church D, Szumski R, Louie TJ. Direct detection of verotoxin-producing Escherichia coli in stool samples by PCR. Journal of clinical microbiology 1995;33:519-24.

53. Wang RF, Cao WW, Cerniglia CE. PCR detection and quantitation of predominant anaerobic bacteria in human and animal fecal samples. Applied and environmental microbiology 1996;62:1242-7.
54. Moller JK, Pedersen LN, Persson K. Comparison of the Abbott RealTime CT new formulation assay with two other commercial assays for detection of wild-type and new variant strains of Chlamydia trachomatis. Journal of clinical microbiology 2010;48:440-3.
55. Cheng A, Qian Q, Kirby JE. Evaluation of the Abbott RealTime CT/NG assay in comparison to the Roche Cobas Amplicor CT/NG assay. Journal of clinical microbiology 2011;49:1294-300.
56. Morre SA, van Dijk R, Meijer CJ, van den Brule AJ, Kjaer SK, Munk C. Pooling cervical swabs for detection of Chlamydia trachomatis by PCR: sensitivity, dilution, inhibition, and cost-saving aspects. Journal of clinical microbiology 2001;39:2375-6.
57. Diamant J, Benis R, Schachter J, et al. Pooling of Chlamydia laboratory tests to determine the prevalence of ocular Chlamydia trachomatis infection. Ophthalmic Epidemiol 2001;8:109-17.
58. Gebre T, Ayele B, Zerihun M, et al. Comparison of annual versus twice-yearly mass azithromycin treatment for hyperendemic trachoma in Ethiopia: a cluster-randomised trial. Lancet 2012;379:143-51.
59. Ellenberg SS FT, DeMets DL. Data monitoring committees in clinical trials: A practical perspective: John Wiley & Sons, LTD; 2003.

Appendix

Niger
Malawi
Tanzania
SAP
Abbreviated Verbal Autopsy Questionnaire
Infant Adverse Events survey
Mobile App Manual
Pfizer Investigator Initiated Research Serious Adverse Event Report Form and Completion Guide
Resistance Breakpoints Table (to be added)
Costing Forms (to be added)

Mortality Reduction After Oral Azithromycin

Manual of Operations and Procedures

The Francis I. Proctor Foundation, Global Health Sciences
University of California, San Francisco
The Carter Center
Niger Ministry of Health

The Dana Center, Bloomberg School of Public Health
Johns Hopkins University
Tanzania National Institute for Medical Research and Muhimbili Medical Center

The London School of Hygiene & Tropical Medicine
College of Medicine, University of Malawi, Blantyre
Malawi Ministry of Health

Table of Contents

ABBREVIATIONS **5**

CHAPTER 1: OVERVIEW **6**

1.1. EXECUTIVE SUMMARY 6
1.2. OBJECTIVES 6
1.3. STUDY SITES 7

CHAPTER 2: CONTEXT **7**

CHAPTER 3: STUDY DESIGN **10**

3.1. RANDOMIZATION 11

CHAPTER 4: STUDY ELIGIBILITY **11**

4.1. ELIGIBLE COMMUNITIES 11
4.2. ELIGIBLE INDIVIDUALS 12
4.3. STUDY SCHEDULE 13

CHAPTER 5: CORE AND NON-CORE STUDY ELEMENTS **14**

5.1. MORTALITY STUDY 14
5.1.1. Core Elements (Malawi, Niger, and Tanzania) *14*
5.2. MORBIDITY/RESISTANCE STUDY 15
5.2.1. Core Elements (Malawi, Niger, and Tanzania) *15*
5.2.2. Non-core Elements *15*

CHAPTER 6: CENSUS **16**

6.1. CENSUS 16
6.2. RANDOM CENSUS VERIFICATION 18
6.3. VERBAL AUTOPSY 19
6.4. VALIDATING THE MORTALITY OUTCOME 20
6.4.1 Niger *20*
6.4.2 Malawi *21*
6.4.3 Tanzania *22*

CHAPTER 7: REGISTERING PARTICIPANTS FOR SPECIMEN COLLECTION **22**

CHAPTER 8: BLOOD SAMPLES **22**

8.1. FINGERSTICK 23
8.2. DRIED BLOOD SPOT FOR MALARIA 24
8.3. HEMOGLOBIN TEST 26
8.4. THICK AND THIN SMEARS FOR MALARIA 27
8.5. MATERIALS FOR BLOOD COLLECTION 28

CHAPTER 9: SPECIMEN COLLECTION FOR RESISTANCE TESTING **29**

9.1. POPULATION 29
9.2 NASOPHARYNGEAL SWABS 30
9.2.1 Materials for Swab Collection for Resistance Testing *30*
9.2.2 Protocol for Tubing and Handling of Samples *31*
9.3 STOOL SAMPLES 32
9.3.1 Stool Specimen Collection *32*
9.3.2 Materials for Stool Specimen Collection *34*
9.4 QUALITY CONTROL MEASURES FOR SPECIMEN COLLECTION 34

CHAPTER 10: COST-EFFECTIVENESS 35

10.1. COSTS 35
10.2 EFFECTIVENESS 35

CHAPTER 11: TRAINING 36

11.1 STANDARDIZATION 36
11.2 PILOT STUDIES 36

CHAPTER 12: SAMPLE ORGANIZATION, TRANSPORT, AND STORAGE 37

12.1 DE-IDENTIFICATION 37
12.2 SPECIMEN TRANSPORT 37
12.3 SPECIMEN STORAGE 38
12.3.1 Short-term Sample Storage 38
12.3.2 Long-term Sample Storage 38
12.4 CATALOG SPECIMENS 38

CHAPTER 13: MICROBIOLOGY AND MOLECULAR LABORATORY PROCEDURES 39

13.1 STORAGE AND TRANSPORT MEDIA 39
13.1.1 Preparation of STGG Transport Medium 39
13.1.2 Preparation of Amies Transport Medium 40
13.1.3 Preparation of Nutrient Agar Medium/Slants 41
13.2 METHODS FOR MALARIA TESTING 42
13.2.1 Blood Smears 42
13.2.2 Dried Blood Spots 42
13.3 METHODS FOR RESISTANCE TESTING 48
13.3.1 Isolation & Identification of Organisms Using Microbiological Techniques 48
13.3.2 Microbiological Resistance Testing for Swabs 50
13.3.3 Nucleic Acid Based Resistance Testing for Stool Specimens 52
13.4 METHODS FOR *CHLAMYDIA TRACHOMATIS* (NON-CORE) 54
13.4.1 Description of Abbott RealTime Assay 54
13.4.2 Processing of Samples for PCR 54
13.4.3 Procedure for Masking Samples 54
13.4.4 Procedure for Pooling Samples 55
13.4.5 Quality Control 55
13.4.6 Quantification 55

CHAPTER 14: STUDY MEDICATION 56

14.1 STUDY MEDICATION DESCRIPTION (FROM PFIZER, INC.) 56
14.1 DOSAGE INFORMATION 56
14.2 MEDICATION PROCUREMENT/DONATION 56
14.3 MEDICATION QUALITY CONTROL 57
14.4 ANTIBIOTIC DISTRIBUTION & MONITORING COVERAGE 57
14.5 ADVERSE REACTIONS/SIDE EFFECTS 57
14.6 ADVERSE EVENTS SYSTEMS 58
14.6.1 Passive Adverse Events Monitoring 58
14.6.2 Active Adverse Events Monitoring 58
14.6.3 Adverse Events Data 60
14.8 SUPPLY ISSUES 60

CHAPTER 15: PROTECTION OF HUMAN SUBJECTS 61

15.1 INSTITUTIONAL REVIEW BOARD APPROVAL 61

15.1.1. Niger ... 61
15.1.2 Tanzania ... 62
15.1.3 Malawi ... 62
15.2 Informed Consent ... 62
15.3 Risks and Benefits of Study Procedures ... 63
15.3.1 Verbal Autopsy ... 63
15.3.2 Swabbing Procedures ... 63
15.3.3 Stool Collection ... 63
15.3.4 Blood Testing ... 63
15.3.5 Anthropometric Measurements ... 63

CHAPTER 16: STUDY MONITORING ... 64

CHAPTER 17: DATA AND SAFETY MONITORING COMMITTEE CHARTER ... 64

17.1 Primary Responsibilities of the DSMC ... 64
17.2 DSMC Membership ... 65
17.3 Conflicts of Interest ... 65
17.4 Timing and Purpose of the DSMC Meetings ... 65
17.5 Procedures to Ensure Confidentiality and Proper Communication ... 66
17.6 Statistical Monitoring Guidelines ... 67
17.7 DSMC Contact Information ... 68

CHAPTER 18: DATA COLLECTION, MANAGEMENT, AND SECURITY ... 69

18.1. Scope of Data ... 69
18.2 Data Storage, Management, and Security ... 69

REFERENCES ... 70

APPENDIX ... 75

Abbreviations

DCC: Data Coordinating Center
GPS: global positioning system
IRB: Institutional Review Board
JHU: The Johns Hopkins University
LSHTM: London School of Hygiene and Tropical Medicine
MUAC: mid-upper arm circumference
NP swabs: nasopharyngeal swabs
PCR: polymerase chain reaction
STGG: skim milk tryptone glucose glycerin media
UCSF: University of California San Francisco
WHO: World Health Organization

Chapter 1: Overview

1.1. Executive Summary

An estimated 7.7 million pre-school aged children die each year, the majority from infectious diseases.[1] Recently, we demonstrated that mass azithromycin distributions for trachoma may have the unintended benefit of reducing childhood mortality.[2,3] This surprising result was observed in an area of Ethiopia with highly prevalent trachoma. Another trial is necessary to determine whether a similar effect will be seen in areas not eligible for trachoma treatments. If mass azithromycin can be proven to reduce childhood mortality, then widespread implementation in developing countries could be feasible and perhaps integrated with other interventions.

Our long-term goal is to more precisely define the role of mass azithromycin treatments as an intervention for reducing childhood morbidity and mortality. We propose a multi-site, cluster-randomized trial comparing communities randomized to oral azithromycin with those randomized to placebo. To assess the generalizability of the intervention, we will monitor for antibiotic resistance, which could potentially limit adoption of mass antibiotic treatments. We will also begin to investigate possible explanatory factors for mortality reduction by assessing several measures of the burden of infectious diseases. We hypothesize that mass azithromycin treatments will reduce childhood mortality even in areas with low trachoma prevalence, and will be accompanied by an acceptable level of antibiotic resistance.

1.2. Objectives

1: To test the hypothesis that mass distributions of oral azithromycin targeted to 1-60 month old children reduces childhood mortality, in a cluster-randomized trial

2: To assess the cost-effectiveness of mass azithromycin in reducing childhood mortality

3: To investigate the most plausible factors that could explain an effect of mass azithromycin on childhood mortality

4: To assess for emergent macrolide resistance among children following mass azithromycin treatments

5: To collect and securely store samples from the nasopharynx, nares, conjunctiva, blood, and stool after azithromycin distributions to assess infectious burden and resistance

Ancillary Aim 1 (non-core): To assess the effect of mass azithromycin treatments on microbial diversity in the conjunctival, nasopharyngeal, nasal, and intestinal biome of children

1.3. Study Sites

A consortium of experienced researchers and program implementers will conduct the study in 3 sites (Malawi, Niger, and Tanzania), chosen for diversity of geography and comorbidities, existence of a functioning national azithromycin distribution program, and previous investigator experience:

- **Southern Africa:** Mangochi district, Malawi (Malawi Ministry of Health, University of Malawi, Blantyre, and London School of Hygiene & Tropical Medicine)
- **West Africa:** Loga and Boboye departments, Niger (Carter Center Niger, Niger Ministry of Health, University of California, San Francisco)
- **East Africa:** Kilosa and Gairo districts, Tanzania (National Institute for Medical Research, Kilosa, Johns Hopkins University)

Chapter 2: Context

Reduction of childhood mortality is an international development goal. The United Nations Millennium Development Goals call for a two-thirds reduction in childhood mortality by 2015.[4] Despite progress this past decade, under-5 mortality remains unacceptably high.[1,5,6] In Africa, where the burden of childhood mortality is greatest, three-quarters of all childhood deaths occur after the neonatal period, with malaria, pneumonia, and diarrhea accounting for the majority.[7] Management is often case detection and individualized therapy, but novel strategies such as mass treatment may play a role.[8-10]

The proposed research will assess whether childhood mortality can be reduced using a novel approach—mass antibiotic administrations similar to those used for trachoma. The World Health Organization (WHO) recommends repeated annual distribution of single-dose oral azithromycin to each resident of trachoma-endemic communities, though more frequent treatments may be necessary.[11-13] While azithromycin effectively clears the ocular strains of chlamydia that cause trachoma,[12,14,15] the drug also has activity against major causes of childhood mortality, including respiratory infections, diarrhea, and malaria.[16-18] Mass treatments have been shown to be effective for these non-chlamydial infections. For example, in a study we conducted in Nepal, the prevalence of diarrhea in children decreased from 32% before a mass

azithromycin treatment, to 11% ten days post-treatment (OR 0.26 [95% CI 0.14 – 0.43]).[19] Others in our consortium have found that malaria parasitemia decreased by 44% after mass azithromycin treatments.[20] It is feasible that periodic mass azithromycin distributions could reduce the burden of these infections sufficiently enough to reduce childhood mortality.[2]

Mass azithromycin treatments targeted to children may maximize the benefit of antibiotics, while limiting resistance. Pre-school children have the most to gain from mass azithromycin treatment because the burden of treatable, life-threatening infection is highest in this age group.[21] Treatment of children may also provide indirect protection to untreated members of the community due to a herd effect.[22] In a trial in Ethiopia, quarterly mass azithromycin treatments targeted to children aged 1-10 years resulted in a 47% reduction in ocular chlamydia in *untreated* community members.[23] Although we demonstrated this indirect protection for ocular chlamydial infection, other infectious diseases may be similar. Reducing the infectious burden of respiratory pathogens, malaria, and diarrhea might benefit children who missed a distribution or were too old for treatment. Given that community antibiotic resistance correlates with the volume of antibiotic consumption,[24-26] targeting children may result in less antibiotic resistance in a community. Targeting children would also be easier logistically and require fewer doses of donated antibiotic, both of which lower costs of administrations.

The finding of a reduction in childhood mortality after mass azithromycin treatment warrants replication in other settings to increase generalizability. The current proposal differs from our earlier trial in several important aspects. First, we will conduct the study in areas with little or no trachoma to enhance generalizability. Second, we will monitor a subset of communities for measures of infectious and nutritional morbidity, to identify explanatory factors for any mortality reduction. Third, we will randomize control communities to placebo, reducing the chance of differential co-intervention and differential outcome assessment. Fourth, we will treat children 1 month and older, as opposed to 12 months and older. Fifth, we will test whether restricting azithromycin to only children will limit selection of community antibiotic resistance in *Streptococcus pneumoniae* and other organisms. Sixth, we will monitor for a longer period of time than the previous study, to assess whether mass azithromycin results in a more sustained mortality reduction. Finally, we will enroll a larger number of children and randomize a larger number of clusters, providing considerably more statistical power to detect even a smaller reduction in mortality.

Mass drug administrations can be implemented relatively easily and potentially have a major impact on morbidity and mortality. Mass treatments

are simple interventions and considerable expertise in their deployment already exists in developing countries.[27] They can have a dramatic impact in some settings. For example, a study of mass distribution of vitamin A for xerophthalmia in the 1980s found that treated communities had lower childhood mortality compared to untreated communities.[28] Trials in the following decade largely confirmed these results.[29-34] Now, vitamin A distributions are provided in most countries that have high childhood mortality, with a majority of programs achieving the targeted 70% coverage.[35] A report estimated that vitamin A could prevent as many as a quarter million childhood deaths each year.[36] This same study estimated that treatment of diarrhea, sepsis, pneumonia, and malaria could prevent over 3 million deaths each year. If mass azithromycin treatments can prevent or treat even a fraction of these infections, this could have a tremendous effect on childhood mortality.

Millions of azithromycin treatments are currently distributed each year. Since 1999, over 225 million treatments of azithromycin have been donated through the International Trachoma Initiative for distribution by local partners in 19 countries.[37] Many Ministries of Health and non-governmental organizations have experience procuring and transporting the drug, organizing local health workers for antibiotic distribution, and monitoring antibiotic coverage. These activities have proven to be sustainable; excluding the cost of the antibiotic itself, mass distribution costs an estimated $0.25-0.40 per person treated.[38] Although the costs of azithromycin would add to this, generic azithromycin is available, and economies of scale would likely reduce drug costs if mass treatments became more widespread.

Chapter 3: Study Design

We will assess childhood mortality over two years, comparing communities where children aged 1-60 months receive biannual oral azithromycin ("Azithro" arm), to communities where the children receive biannual oral placebo ("Control" arm). We will also randomize a separate set of communities to these treatment strategies, but monitor instead for infectious disease morbidity and macrolide resistance ("Azithro-Plus" and "Control-Plus"). These communities will not be included in the mortality comparison, since identification and treatment of morbidities could interfere with any mortality effect of azithromycin. All biologic specimens collected as part of the morbidity and resistance outcomes will be stored and made available to other investigators for further laboratory testing at the conclusion of the study, per Gates Foundation guidelines.

Figure 1: Trial Profile

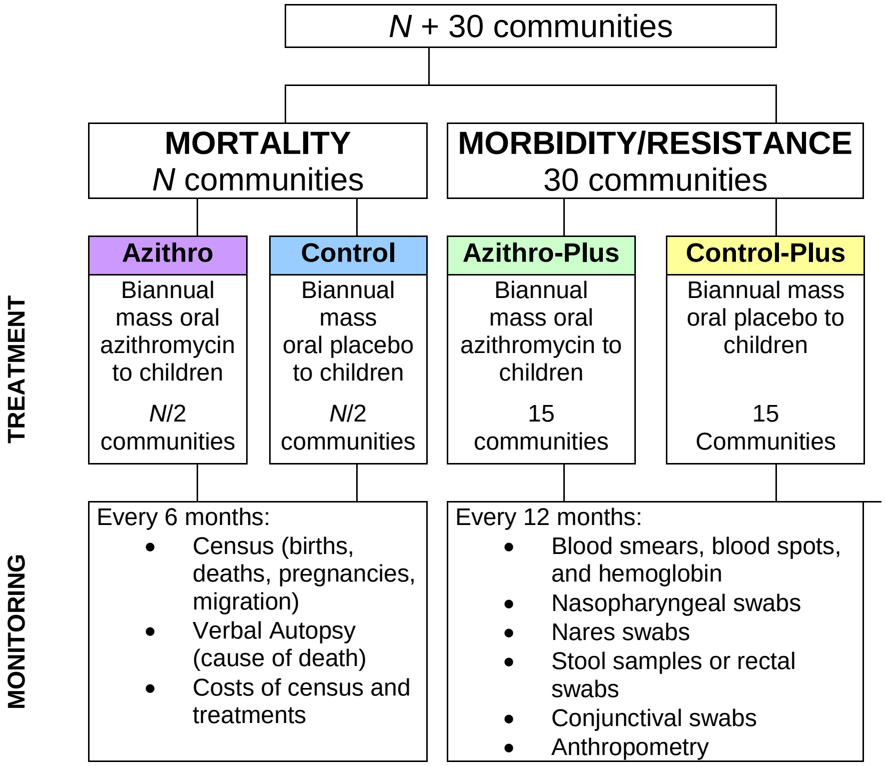

3.1. Randomization

Randomization of Treatment Allocation. In each site, communities within a contiguous area of 300,000 to 600,000 individuals will be randomized to azithromycin or placebo for the mortality study. An additional 30 communities will be randomized into one of two study arms for embedded, cost-efficient, morbidity studies. Since mortality and morbidity study communities will be drawn from the same pool, findings from one study will be relevant to the other. Each site has a different set of prevalent co-morbidities and different access to health interventions.

No stratification or block randomization is planned within each country. The allocation of community assignments will be conducted for each country separately using simple random sampling. For each country, all study arms (mortality and morbidity) will be assigned from the same list of eligible communities. A pseudorandom number scheme will be implemented using the statistics package R. Randomization will be conducted by T. Porco. Procedural and algorithmic details are provided in an appendix to the Statistical Analysis Plan.

Study Participants: At months 0, 12, and 24, a random sample of children will be selected using a computer-generated simple random sample for exams and sample collection to monitor for morbidity and resistance.

Chapter 4: Study Eligibility

4.1. Eligible Communities

To be eligible for the trial, a community must meet the following criteria:

1. The community location in target district.
2. The community leader consents to participation in the trial (this does not obviate the need for individual consent, but without overall leadership consent, the community as a whole cannot be part of the trial).
3. Eligible communities estimated population of between 200–2,000 people.
4. The community is not in an urban area.

4.2. Eligible Individuals

Mortality Study:

Census: The study can be thought of as consisting of four 6-month segments, each of which starts with a census and ends with a follow-up census. All children in the study communities aged 1-60 months (up to but not including the 5th birthday) at the initial census of each segment are eligible to participate in the subsequent 6-month segment of the study. Note that the information for children erroneously entered into the census (e.g., children younger than 1 month or ≥ 60months) can be corrected at the subsequent treatment, subsequent census, or at a verbal autopsy later in the study. However, these changes will not be applied retroactively; these misclassified children will still be included in the study population for that 6-month segment and any deaths will be counted toward the primary outcome. In addition, all children listed on the initial census for that 6-month segment will be included in the outcome, regardless of whether they received the study drug.

Treatment: Individuals allergic to macrolides or azalides will not be given the study antibiotic azithromycin, but will be included in the outcome.

Morbidity/Resistance Study:

Census: The criteria for being included in the census are the same as the Mortality Study, as described above.

Treatment: The inclusion and exclusion criteria for treatment are the same as for the Mortality Study, as described above.

Examination & Sample Collection: A random sample of children aged 1-60 months (up to but not including the 5th birthday) are eligible for examination and sample collection. As described above, the random sample will be a simple random sample based on the previous census. These individuals will likewise be selected from the previous census.

> **Note:** All individuals must be registered on the official census in order to be considered part of the study.

4.3. Study Schedule

The schedule for examination and treatment is shown below in Table 1:

MORDOR Study Schedule

	MORTALITY		MORBIDITY / RESISTANCE	
	**Azithro**	**Control**	**Azithro-Plus**	**Control-Plus**
	**Oral Azithromycin (*N/2* communities)**	**Oral Placebo (*N/2* communities)**	**Oral Azithromycin (N = 15 communities)**	**Oral Placebo (N = 15 communities)**
**MORDOR 0 Jan-15 to Jul-15***	Census<br>Azithro	Census<br>Placebo	Census<br>Swabs<br>Blood<br>Stool<br>Anthropometry<br>Azithro	Census<br>Swabs<br>Blood<br>Stool<br>Anthropometry<br>Placebo
**MORDOR 6 Aug-15 to Jan-16**	Census<br>Verbal Autopsy<br>Azithro	Census<br>Verbal Autopsy<br>Placebo	Census<br>Azithro<br>*NP Swabs, Blood, Stool* [a]<br>*Malaria Collection*[b]	Census<br>Placebo<br>*NP Swabs, Blood, Stool* [a]<br>*Malaria Collection*[b]
**MORDOR 12 Feb-16 to Jul-16**	Census<br>Verbal Autopsy<br>Azithro	Census<br>Verbal Autopsy<br>Placebo	Census<br>Swabs<br>Blood<br>Stool<br>Anthropometry<br>Azithro	Census<br>Swabs<br>Blood<br>Stool<br>Anthropometry<br>Placebo
**MORDOR 18 Aug-16 to Jan-17**	Census<br>Verbal Autopsy<br>Azithro	Census<br>Verbal Autopsy<br>Placebo	Census<br>Azithro<br>*NP Swabs, Blood, Stool* [a]	Census<br>Placebo<br>*NP Swabs, Blood, Stool* [a]
**MORDOR 24 Feb-17 to Jul-17**	Census<br>Verbal Autopsy<br>**Azithro**[c]	Census<br>Verbal Autopsy<br>**Azithro**[c]	Census<br>Swabs<br>Blood<br>Stool<br>Anthropometry<br>**Azithro**[c]	Census<br>Swabs<br>Blood<br>Stool<br>Anthropometry<br>**Azithro**[c]

Only children aged 1 month to 60 months per community will be treated
Arms Azithro+ & Control+: swabs, blood testing, and stool samples or rectal swabs from 40 randomly selected children aged 60 months per community; anthropometry from a cohort of children aged 1 to 60 months per community; NP swabs from 40 individuals aged 7 – 12 years (Niger only)

a: Tanzania only
b: Niger only
c: Decision to treat ALL communities with azithromycin at 24-months if there is a significant mortality difference (in preparation for possible contingent study, Niger only)
****: +/- 1 month***

Chapter 5: Core and Non-Core Study Elements

An overview of core and non-core elements for the mortality and morbidity study is provided here, but will be described in more detail in the following chapters.

5.1. Mortality Study

5.1.1. Core Elements (Malawi, Niger, and Tanzania)

In all three study sites, we will conduct the following study activities for the mortality study:

Training

Before the baseline visit, the University of California San Francisco (UCSF) team will certify local team members at all three study sites. Standardization activities before each biannual census will consist of didactic classroom instruction and mock census activities, followed by in-field training to harmonize protocols.

Census

An enumerated population census for 0 – 60 month olds (focusing on this age group) will be conducted every 6 months by trained field workers masked to study arm, recording births, deaths, and migration of children eligible for treatment. Pregnant women will be noted at each census, to maximize inclusion of newborns on the subsequent census.

Random Census Verification

A repeat census will be conducted in a random selection of households at each study visit. Personnel will be different from the original census. This will allow us to assess whether the census team identified all births, deaths, and migratory episodes relative to the previous census.

Verbal Autopsy

Each of the study sites will perform a verbal autopsy for all children (aged 1-60 months) identified as having died during the study.[39]

Treatment

Children aged 1-60 months on the current census will be offered weight- or height-based (<1 year weight and children who cannot stand; ≥1 year height), directly observed, oral azithromycin suspension (or oral placebo) every 6 months for 2 years as performed in trachoma programs in each of the 3 countries. Specifically, individuals are eligible on or after their one month birthday, and prior to the day of their fifth birthday.

Antibiotic Coverage Surveillance
We will estimate antibiotic coverage from the most recent biannual census records. At the end of each treatment round at months 6, 12, and 18, we will identify any children who have missed 2 or more consecutive treatments, and forward this information to the census team.

5.2. Morbidity/Resistance Study

5.2.1. Core Elements (Malawi, Niger, and Tanzania)

All core elements described in 5.1.1 will also be conducted in the morbidity / resistance study. The enumerated population census for the morbidity/resistance communities will include 0-12 year olds.

The designation "core elements" means that all study sites will be performing the study activity.

In all study sites, we will perform the following tests on a random sample of 40 children aged 1-60 months from each Azithro-Plus and Control-Plus community at baseline, 12, and 24 months:
- Blood samples (dried blood spots optional) for malaria and anemia
- Nasopharyngeal swabs for pneumococcal macrolide resistance
- Stool samples or rectal swabs to assess for macrolide resistance
- Assessment of costs of a census and mass drug administration (at 6 or 12 and 24 month visits only)

Samples will be processed in-country when possible; otherwise they will be shipped to UCSF.

5.2.2. Non-core Elements
The designation "non-core" element means that study sites may choose if they wish to perform the study activity. If the study sites perform the study activity, the methodology will be performed according to this protocol so that the 3 sites may be more easily compared. Sites may choose to perform several non-core morbidity assessments at the 0, 12, and 24 month visits:
- Nasal swabs for macrolide resistance in *Staphylococcus aureus*
- Conjunctival swabs for *Chlamydia trachomatis*
- Conjunctival examination and photography to assess for trachoma
- Anthropometric assessments
- Nasopharyngeal swab collection at local health posts to compare macrolide resistance in the community with that in the health post

In addition, there are several non-core activities that occur outside the annual morbidity/resistance assessment:

- Additional study visit during the first malaria season to collect blood samples for malaria and anemia testing
- Clinic-based case finding for childhood illnesses at all health clinics in the study area

Costs

We anticipate recording the cost of distributions in a random subset of 12 villages at the 6 or 12 and 24 month visits.

Details of the non-core elements for each study site can be found in the Appendices.

Chapter 6: Census

6.1. Census

Census Team

Census workers will be selected by the site study coordinator. These individuals may have different qualifications and educational backgrounds in the 3 sites, but, at a minimum, each census team member should be computer-literate, such that they are able to operate a tablet computer and type on its keyboard. Census workers will ideally be local community dwellers, and familiar with the communities being enumerated. In addition, several supervisors will be present for the duration of the census to monitor census workers.

Census Training

Census workers will be trained at the beginning of the study and refresher trainings will be offered as needed for the duration of the study. Training will start with reviewing the census data collection software on the tablet computer, care of the tablets, charging of the tablets, etc. The training will then proceed to a demonstration of the use of the software at a mock household, including common problems that staff may encounter (e.g., no one at home, GPS function not working, software crashing). In the final part of the training, the study coordinators and investigators will accompany team members to several communities and observe the census activities.

Census Software

The census will be directly entered into a tablet computer. The software will capture information about each child aged 0-5 (or 0-12 in morbidity communities) in each household: name, age, sex, father's name, and mother's name, and will also register any pregnant women. The GPS coordinates will be

documented for each household at the entrance to the household. At follow-up censuses, team members will identify each household on the existing census, and will update the status for each child:

- STATUS: Alive, slept in household last night
- STATUS: Alive, but not in household
    - ABSENCE: <1 month
        - Is he/she coming back within 1 week?
            - Yes (mop-up)
            - No
            - I don't know
        - INFORMANT: household member, neighbor, village chief, other
    - ABSENCE: ≥ 1 month
        - MOVE: Moved within community
            - INFORMANT: household member, neighbor, village chief, other
        - MOVE: Moved outside of community
            - INFORMANT: household member, neighbor, village chief, other
- STATUS: Died
    - PLACE: Child living in community when died
        - INFORMANT: household member, neighbor, village chief, other
    - PLACE: Child had moved out of community when died
        - INFORMANT: household member, neighbor, village chief, other
- STATUS: Unknown
    - INFORMANT: household member, neighbor, village chief, other

Whenever a new individual is added to the census, the software will automatically assign each individual to a universal unique identification number as well as a study identification number.

Census Data Uploading

The census will be collected on tablet computers with 3G mobile and Wi-Fi capabilities. There will be 3 options for uploading data to the database. First, and most desirable, a local SIM card with data plan can be purchased for each tablet, and the data uploaded via cell towers twice per day (midday and at the end of the day). This option is most desirable because it minimizes data loss in the case of a lost, stolen, or damaged device. In addition, this option will not require each tablet computer to be in contact with a Wi-Fi hub. As a second option for uploading, each census supervisor will have access to a Wi-Fi hub, and the

supervisor can visit the census teams to upload data regularly. This option is less desirable, because the data will be uploaded less frequently. As a third option, the data can be uploaded at a central study site, either via Wi-Fi or micro USB cable directly into a computer. This option is least desirable because is not feasible to take the tablet computers to the central site regularly given the large geographical areas of the study.

Census Supervision

The site study coordinator will supervise all census activities. Formal checks of census quality will be conducted through the random census verification. In addition, the study coordinator will visualize all censused households using imagery from GoogleMaps. The goal of this activity will be to minimize the chances of missing large neighborhoods or specific regions within study communities. The study coordinator will also check the data entry progress for each community, paying special attention at the follow-up censuses, as to whether there are any missing data for the "vital status" variable (i.e., present, dead, absent). Once the study coordinator is confident that the entire community has been reached, and that the amount of missing data are acceptable (defined as <10% of children in a community), the study coordinator (or other research team member) will certify the census data collection for that community complete via Salesforce. Changes can be made to the record at different time points, but these changes will not be reflected until the subsequent census. All changes are time and date stamped in the database.

Census Timing

The census will be performed prior to each mass azithromycin/placebo distribution. Study sites may choose to perform the census activities over a discrete time period (e.g., all communities completed over a 1-month period, requiring census activities to take place simultaneously in many communities at once) or alternatively in a "rolling" fashion (e.g., all communities completed over a 6-month period, requiring fewer census teams to be active at once). In either case, each community must be censused every 6 months, so it may not take more than 6 months to complete all communities. The census must be completed (i.e., "locked") at the household level before treatment can be given.

6.2. Random Census Verification

A random re-census of households will be conducted at each study visit by supervisors, additional census team members, or local community monitors, as appropriate for the study site. Verification will be performed at the household level, with a minimum of 200 households being resurveyed during the 5 study visits per country (approximately 40 per visit). Each team must have at least one household census verified at each study visit.

Households will be selected using a different mechanism than the mechanism used by the original census. The primary method for selecting random households will be from aerial visualization (Google maps, AfriPop, etc.) If aerial visualization is not possible in an area, then another method for obtaining households can be used, such as a random walk.

Both census teams will use the same electronic template (i.e., no prior records at MORDOR 0, and the census records from the prior census at each follow-up visit). We will arbitrarily select a sample of communities stratified by census team. Once the census has been completed, the trial biostatistician will analyze the communities for verification.

The trial biostatistician will compare the results of the original census and the re-census to identify any discrepancies. The steering committee will determine any corrective actions once this comparison is made. At the very minimum, the site study coordinator will inform the original census team of the discrepancies and will conduct a refresher training session to minimize data collection errors.

6.3. Verbal Autopsy

Verbal autopsy questionnaires will be completed for all deceased children (aged 1-60 months).

Staff

Verbal autopsy interviews will be conducted by trained staff. Training will focus on conducting sensitive interviews with persons who may still be in mourning; discussion of verbal autopsy questions; reviewing the format of the paper and/or electronic questionnaires (including skip logic); and demonstration of the verbal autopsy technique on 5 mock deaths. The 5 first verbal autopsies will be observed by the study coordinator to ensure that proper procedures are followed. Each verbal autopsy interviewer will be responsible for a distinct geographic area, and will be responsible for regular contact with the key informant from each community.

Identification of Deaths

Deaths will be identified in 2 ways: from the biannual census, and from the key informant system. The trial biostatistician will provide a list of all deaths to the site study coordinator after each census. This list will include information on the deceased child’s name, age, gender, and unique identification number; community name; and father’s and mother’s names. The study coordinator will in turn deliver this list to the appropriate verbal autopsy interviewer. The verbal autopsy interviewer will also keep a record of all deaths identified by the key

informants. In each case, the key informant will report the community name, child's name, and parents' names, and the deceased child will be located in the census database.

Questionnaire Administration
Each site will choose between the full-length WHO questionnaire (2007 version) or an abbreviated form (which utilizes questions from both WHO 2007 and 2012), translated into the local language as appropriate. Questionnaires will be administered at the home of the deceased child. The informant will be the deceased child's parent or guardian. If this person is not available, the verbal autopsy interviewer will try to arrange a time to return to interview this person. If the parent or guardian is not present on the third visit, they will complete the questionnaire by interviewing another family member, or as a last resort, a neighbor. All interviews will be completed in the local language. Our goal is to perform each verbal autopsy within 2 months of identification of death. The child's name and unique identification number will be recorded on the verbal autopsy record for identification purposes.

Assigning the Cause of Death
As recommended by the WHO, we will use automated methods to assign causes of death based on the verbal autopsy questionnaire, rather than physician review. We will treat all individuals under 4 weeks as one subpopulation, and individuals 1-60 months as a separate subpopulation for determination of cause-specific mortality fractions.

Translation
The verbal autopsy will be translated into the local language by a professional translator and then back-translated into English by a second translator. Discrepancies that are noted in the back-translation will be provided to the original translator, and adjustments made as needed.

At each country site, the questionnaire will be validated and reviewed to ensure that it is culturally appropriate.

6.4. Validating the mortality outcome

6.4.1 Niger
Deaths from the biannual census will be validated from local death registries. In an effort to improve the quality of existing death registries in villages and health facilities, the Study Coordinator will conduct sensitization activities in study communities and CSIs before the study begins. During sensitization, the Study Coordinator will work closely with local village chiefs, mayors, and health

centers to develop a strategy for obtaining more accurate death registers during the study.

CSIs and villages maintain local death registries. Health professionals record deaths that occur in the CSI. The CSI death registries include: name, age, village, cause of death. The village chief also maintains a death registry in the village. The village death registry includes: name, age, village, cause of death.

The Study Coordinator will visit each CSI and take photos of local death registries with all deaths from children aged 0-9 years. We collect a larger age group than that collected in the census since age misclassification may occur either on the census or on the death registry. The Study Coordinator will keep all information present in the registry for all children aged 0-9 years, including name and village.

The Data Manager will be responsible for linking the death registry file with the census file, using the name, age, and village of deceased children. We expect that both the census and the registry will miss deaths.

Verbal autopsies will be performed on all deaths picked up by the census and also all deaths from 0-5 year-olds picked up by the death registry. Note that the deaths from the death registry will not be used in the primary outcome.

6.4.2 Malawi

Deaths will be identified in 2 ways: from the biannual census, and from the key informant system. The trial biostatistician will provide a list of all deaths to the site study coordinator after each census. This list will include information on the deceased child's name, age, gender, and unique identification number; the community name; and the father's and mother's names. The study coordinator will in turn deliver this list to the appropriate verbal autopsy interviewer. The verbal autopsy interviewer will also keep a record of all deaths identified by the key informants. In each case, the key informant will report the community name, child's name, and parents' names, and the child will be located in the census database. We will provide phone credit as appropriate for the key informants.

Trained verbal autopsy interviewers will be responsible for a distinct geographic area, and will be responsible for being in regular contact with the key informant from each community. We will aim to perform all verbal autopsies within 2 months of death. The interviewee will be the deceased child's parent or guardian. If this person is not available, the verbal autopsy interviewer will try to arrange a time to return to the home to interview this person. If the parent or guardian is not present on the third visit, they will complete the questionnaire by

interviewing another family member, or as a last resort, a neighbor. All interviews will be completed in Chichewa, Yao or English, as appropriate, on a tablet computer.

6.4.3 Tanzania

Community Monitors will visit a random sample of households identified on the census as having children. They will visit their assigned households each month to monitor for deaths. These records will be kept apart from the data collected by the census teams (at six month intervals). The two lists will be compared and any discrepancies followed up.

Chapter 7: Registering Participants for Specimen Collection

Samples will be collected with reference to age, gender, household, and community, but participant names will not be included in laboratory records to ensure privacy. Samples will thus not be associated with an individual's name, but with a random identification number and/or QR code, masking laboratory personnel and preventing identification of individuals.

At each time point, each child selected for examination/specimen collection will be assigned an identification number for database anonymity.

For each community, the randomized registration list for examinations will be generated by the database and downloaded to the tablet using the mobile application.

After registration, the child and his/her guardian will be directed to the appropriate examination stations.

Chapter 8: Blood samples

Protection of Examiner and Study Participant

Prior to examinations at the blood station and the swab station, the examiner and tuber must be gloved. The examiner will put latex gloves on both of his/her hands prior to touching the participant and a new pair of gloves will be used for each participant in order to avoid transmitting infection between participants. Purell® Instant Hand Sanitizer will be available for hand sanitization when needed.

We will collect:

1) Thick and thin smears, assessed for malaria parasitemia and gametocytemia by trained local microbiologists,

2) Microcuvettes, analyzed for hemoglobin in the field using a HemoCue analyzer (HemoCue AB, Ängelholm, Sweden), and
3) Dried blood spots, collected on FTA Elute cards (Whatman, Kent, UK; or appropriate substitution) and sent for laboratory testing for malaria using a nested PCR assay[40] and/or TropBio cards (Tropbio Pty Ltd, QLD, Australia) and sent to the CDC for serological testing.

The order of events at the blood collection station is: 1) finger prick; 2) blood spots on filter paper; 3) hemoglobin test; 4) thin and thick smears for malaria.

It is important to handle all blood specimens with care to minimize risk of infection

Wear gloves. New gloves must be worn for each child.
Clean spills. In the event of a blood spill or splash, clean immediately with approved disinfectant (10% bleach or chlorhexidine solution) and wipe with absorbent material.
Disposal of sharps. All lancets must be disposed of properly in sharps containers.
No food. Food and drink are not allowed at the blood collection station.

8.1. Fingerstick

Inform the mother that her child's finger will be pricked to obtain blood to test for malaria and anemia. Describe the finger prick procedure, reassure her, and answer all questions. The blood specimen should be collected as described below to minimize the discomfort of the child and to ensure sufficient blood volume collection.

A finger stick of capillary blood will be collected for thin and thick blood smears to assess for malaria, hemoglobin testing, and dried blood spots to be stored for later testing. Blood will be collected by a gloved health worker using aseptic technique. Gloves will be changed between each participant. The fingerprick or heelstick site will be disinfected using a 70% isopropyl alcohol swab.

Fingerstick procedure:

1. Prepare the disposable lancet. Use a NEW disposable lancet for each child. **Do not** re-use lancets!
2. The recorder will scan the child's QR code, and place a random number sticker on the TropBio filter paper and the (right edge of the) slide.

3. Position the child for the finger stick. Make sure that the child's right hand is warm and relaxed. Hold the child's thumb, middle, or ring finger on his/her right hand (from the top of the knuckle to the tip of the finger) between your left thumb and finger and disinfect in small outward circles with an individually packaged alcohol wipe.
4. After the alcohol dries, use the thumb to lightly press the child's thumb or finger from the top of the knuckle towards the fingertip to stimulate blood flow towards the sampling point (puncture site). For the best blood flow and least pain, prick the side of the thumb/fingertip, not the center. While applying light pressure towards the thumb/fingertip, hold the lancing device in your hand and prick the thumb/finger. If the finger prick is performed properly, a single prick should be sufficient to collect the required amount of blood.
5. Allow the blood to ooze out. Wipe away the first 2 or 3 drops of blood with gauze. If necessary, re-apply light to moderate pressure towards the thumb/fingertip (approximately 1 cm behind the site of the finger prick) until another drop of blood appears.
   Note: **Do not** squeeze forcefully. Avoid "milking" as it may dilute the blood with tissue plasma.

8.2. Dried Blood Spots for Malaria

Collecting the FTA Elute filter paper sample:

1. Label the filter paper with a random number sticker.
2. Place 2-4 large drops of blood directly from the thumb or finger onto the large circle on the filter paper (if it is difficult to obtain 4 drops of blood, it is sufficient to collect 2 drops of blood).
3. Leave the filter paper to air dry for a few minutes, then place the sample into a small plastic bag along with a desiccant packet.
4. Leave the bag open for a few minutes more, and when the blood is **completely** dry, roll down the top of the bag and close with a piece of masking tape.
5. Store the filter paper samples (in small plastic bags) in a larger Ziploc bag. Keep all filter paper samples in a safe, dry place at room temperature.
6. Blood spots will be stored at room temperature in a locked cabinet in the study coordinators' office.

Collecting the TropBio filter paper sample:

1. Label the filter paper with a random number sticker.
2. Grip the filter paper on the side without small circles. Place a droplet of blood directly from the thumb or finger onto five of the six circles, leaving the right one blank. Be sure to fill each circle completely.

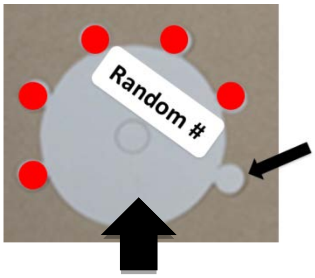

Leave last circle blank

Area to hold the filter paper.
Do not touch the small circles.

3. The recorder will scan the QR code.
4. Carefully slide the filter paper onto a pencil to air dry for at least an hour. There should be about 1 cm in between each sample. Secure the pencil into a Styrofoam surface in a box or container to protect from dust.
5. When the filter paper is dry, place each sample into a small zip plastic bag (individually). Place the small bags into a larger Ziploc bag with five desiccant packets.
6. Ensure the large Ziploc bag is sealed tightly, as moisture will damage the samples. Transport these filter paper samples to a freezer.

Set-up drying area for TropBio bloodspots
Supplies: pencils, Styrofoam, cardboard box, paper
- Place Styrofoam in cardboard box
- Put pencils inbox/container – space apart
- *Note:* When placing the blood spot samples on the pencil, space apart by ~2.5cm with pieces of paper in between each sample.

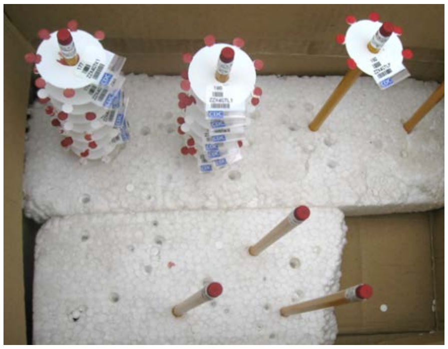

8.3. Hemoglobin Test

A portable spectrophotometer (HemoCue, Anglom, Sweden) will be used for hemoglobin testing.

Set up HemoCue Analyzer

1) Remove the HemoCue analyzer from the case. If a small battery symbol appears on the top right side of the display, the batteries are low. The HemoCue will still give accurate results, but it is strongly recommended to replace the batteries as soon as possible.
2) Pull the cuvette holder out to the loading position. Press and hold the left button until the display is activated (ALL symbols appear on display). The display will show the version number of the program, an hour-glass symbol and "Hb." At this time, it will perform an automatic SELFTEST to verify the performance of the device. After 10 seconds, the display will show three flashing dashes and the HemoCue symbol. This means that the HemoCue has passed the SELFTEST and is ready for use. If the SELFTEST fails, an error code will be displayed.

Collecting a blood sample for hemoglobin (HemoCue):

1. Remove a cuvette from the container. Reseal the container immediately. (The recorder can help the examiner with this.)
2. When the blood drop is large enough, fill the microcuvette in one continuous process. **Do not refill!** If there is not enough blood to fill the microcuvette, you must start again with a new microcuvette. Wipe any excess blood from the sides of the microcuvette with clean gauze or a paper towel, but be careful to avoid touching the open end of the microcuvette so blood is not removed.
3. Look for any air bubbles in the filled microcuvette. If air bubbles are present, discard the microcuvette and obtain a new drop of blood using a

new microcuvette. (Small bubbles around the edge of the microcuvette can be ignored.)

4. Place the filled microcuvette in the cuvette holder. Gently slide the cuvette holder to the measuring position to be analyzed immediately. (This **must** be performed within 10 minutes after filling the microcuvette.)
5. After 15 – 60 seconds, the hemoglobin value will be displayed. The examiner should read the hemoglobin value aloud so the recorder can enter it into the tablet computer. The value will remain on display as long as the cuvette holder is in the measuring position. The analyzer will turn off automatically after 5 minutes.
   Note: For children 6 months to 5 years, if the hemoglobin is <11.0 g/dL, the child is anemic. For children 5 – 11 years, if the hemoglobin is <11.5 g/dL, the child is anemic (WHO/UNICEF/UNU, 1997). If a child is found to be severely anemic, the examiner must refer him/her to the nearest health center for treatment.
6. Carefully dispose of the used microcuvette in the sharps container.
7. **At the end of the day:** Turn off the HemoCue analyzer. Press and hold the left button until the display reads OFF. The display should be blank.

Cleaning the HemoCue Analyzer

1) To clean, pull the cuvette holder to the loading position.
2) Carefully press the small catch (upper right corner of the cuvette holder). Continue to press the catch and carefully rotate the cuvette holder as far left as possible, and then carefully pull the cuvette holder out of the analyzer.
3) Clean the cuvette holder with alcohol or a mild detergent. Push a clean cotton tipped swab moistened with alcohol (without additive) into the opening of the cuvette holder and move from side to side 5 – 10 times. If the swab is dirty, repeat with a new clean swab until the cuvette holder is clean. A dirty cuvette holder may cause the HemoCue analyzer to display an error code.
4) After 15 minutes (or less time, depending on the climate), you may replace the cuvette holder and use the analyzer. The cuvette holder must be completely dry before you replace it.
5) Put the Hemocue analyzer back into its case.

8.4. Thick and Thin Smears for Malaria

1. Label the slide with a random number sticker.
2. For the thick blood smear:
   a. Place a drop of blood in the center (1 cm from the edge of the slide) of a clean, dust-free, and grease-free slide.

   b. Spread the drop of blood evenly with a disposable wooden applicator or with another clean slide into a circle with a diameter of 1 cm.
   c. The blood smear should be about 1cm away from the edge of the slide. The correct thickness of a thick blood smear is one through which newsprint is barely visible when the blood is still wet.
3. For the thin blood smear:
   a. Place a smaller drop of blood on the slide.
   b. Using another slide angled at 45°, create a feathered edge before reaching the other end of the slide.
4. Allow the blood smears to air dry flat. Do not heat the slides, as this will damage the parasites. Be sure to protect the slide from dust and insects. Do not refrigerate slides, as this may cause the smears to detach from the slide during the staining procedure.
5. When dry, place the thick and thin blood smears into the slide box.
6. Smears will be transported at room temperature each day to a diagnostic facility near the study area.
7. Within 24 hours of thick and thin blood smear collection, the smears will be stained with 2% Giemsa stain for 30 minutes. (The thin smear will be fixed by submerging it in 100% methanol for 30 seconds and then let it air dry for 1-2 minutes prior to the Giemsa stain.)
8. Parasite density will be measured by a masked reader using a microscope at the diagnostic facility.

Smears will be stored at room temperature.

A rapid diagnostic test (RDT) could be substituted for thick smears if approved by the Steering Committee.

8.5. Materials for Blood Collection

<u>Fingerprick</u>
Gloves
Disposable lancets
Alcohol wipes
Cotton balls
Gauze
10% household bleach or 4% chlorhexidine solution to clean spills
Absorbent material for spills
Sharps container

Dried Blood Spots
FTA Elute cards
Small zip plastic bags
Desiccant packs
Masking tape
Large Ziploc bags (handful)
TropBio circular cards
Small zip plastic bags
Desiccant packs
Large Ziploc bags (handful)
Materials for drying apparatus: 12 sharpened pencils, Styrofoam, empty cardboard box

Hemoglobin Test
HemoCue machine
Extra set of AA batteries
Cuvettes
Q-tips (handful)

Thick and Thin Blood Smears
Glass slides
Slide box

Rolls of random number stickers

Google Nexus 7
External battery pack

Chapter 9: Specimen Collection for Resistance Testing

9.1. Population

We will collect nasopharyngeal and stool samples on a random set of 40 children aged 1-60 months from each of the communities in the Morbidity study. Children will be selected from the current study census. The swabbing visits will occur after the census but **before** treatment. The randomized registration list of children will be provided to the site study coordinator before the swabbing visit. The Study Coordinator will give this list to the community for mobilization prior to examinations.

9.2 Nasopharyngeal Swabs

Nasopharyngeal swabs will be stored in DNA/RNA shield media by Zymo or STGG media, and standard microbiologic techniques will be used to isolate *S. pneumoniae* and test for resistance to azithromycin, penicillin, and clindamycin. Resistant isolates will be assessed for the most common genetic resistant determinants (*ermB* and *mefA*) using a PCR-based assay.[41] Serotype will be assessed using a nested PCR reaction for the most common serotypes, followed by the Quellung reaction for any untyped isolates.[42]

The examiner will:

1. Place a pediatric flocked swab with a nylon tip through the right nostril and down the nasopharynx of each participant. Note that if the swab is not perpendicular to the frontal plane of the face, it is likely not in the inferior turbinate.
2. Once you reach the nasopharynx, rotate the swab 180° as you remove the swab from the nose.
3. Place the swab in a tube containing 1.0 mL DNA/RNA shield media by Zymo or STGG (skim milk, tryptone, glucose, and glycerin) media, cut the handle off using sterile scissors, and close the cap of the tube with the swab immersed.
4. The nasopharyngeal swab samples in STGG will initially be stored in the field at 4°C using an insulated storage bag with Fisher brand ice gel packs, and then transferred to -20°C. The nasopharyngeal swab samples in DNA/RNA shield media will be stored in ambient temperature in the field. Then transferred to a refrigerator or freezer.
5. The scissors used to cut calcium alginate swabs will be sterilized with alcohol pads or cleaned with bleach wipes between participants. When collecting specimens in DNA/RNA shield, scissors will be cleaned between participants - first with bleach wipes, and then with alcohol pads.

Do not attempt to collect the NP swab if you are not successful after **three** attempts.

9.2.1 Materials for Swab Collection for Resistance Testing

Swabs

NP specimens will be collected using sterile, individually-wrapped pediatric flocked swabs with a plastic swab shaft (manufactured by Copan). Nasal sites will be swabbed with a sterile, Dacron polyester-tipped swab with a plastic shaft (manufactured by Fisherbrand).

Sample Tubes

All field samples for DNA testing will be collected into sterile 2.0ml microcentrifuge tubes, manufactured by Sarstedt®. (DNA-free tubes will be used for collection in DNA/RNA shield.)

Cooler Bags with Frozen Ice Packs
Insulated cooler bags will be used to carry samples to and from the field. In addition, frozen gel ice packs designed to thaw slowly will be used to maintain the temperature in the cooler bags during transport.

-20°C Freezer
A standard -20°C freezer located at the local health center will be used strictly for the storage and freezing of ice packs and samples. This freezer is kept in a locked room on the grounds of the Health Center, which is under 24-hour security guard supervision.

9.2.2 Protocol for Tubing and Handling of Samples
The tubing and handling protocol must be carefully followed in order to prevent contamination and ensure the safe transport of the samples back to the local microbiology laboratory and/or to the US for processing. The person in charge of labeling, tubing, arranging, and handling the samples needs to perform this task in the most orderly and attentive manner.

1. Both hands of the tuber should be gloved at all times. The tuber's gloves only need to be changed when any potential contamination of the gloves occurs. The tuber opens the capped, hinged lid of a microcentrifuge tube, which has been labeled with the participant's random identification number.
2. The swab is inserted by the examiner into the microcentrifuge tube held by the tuber. The swab shaft should only be inserted until the swab head is fully in the tube. The tuber will cut the swab shaft with sterile scissors.
3. The tuber should screw the cap of the microcentrifuge tube tightly, flick the tube to mix the sample with the media (for tubes with DNA/RNA shield media), and place it in the sample collection box, located in the cooler bag filled with frozen ice packs. The flap of the cooler bag should be closed between each patient. The cooler bag should be in as cool a place as possible in the field, in a shaded area out of the sun.

Upon returning from the field each day, the samples in STGG will be immediately taken to a local health center and stored in a commercial -20°C freezer, reserved solely for storage of specimens and ice packs. All samples will be in sample boxes, labeled with the village name for easy future identification.

9.3 Stool Samples

All study sites will collect either stool samples or rectal swabs for resistance testing, but methodology will depend upon the type of samples collected and consist of either culturing/microbiological testing or nucleic acid based testing.

Rectal Swabs for Culturing/Microbiological Testing

Rectal swabs will be collected and placed into Amies transport media or Norgen Stool Preservative. We will use standard microbiologic techniques to isolate *Escherichia coli*. Resistance to azithromycin, ampicillin, and co-trimoxazole will be determined. Isolates can be further classified into commensal and diarrheagenic subtypes using a multiplex PCR assay.[43]

Stool Samples for Nucleic Acid Based Testing

Stool specimens will be used to look for the presence of E. coli and macrolide resistant determinants typically associated with resistant strains of E. coli, using a resistome approach. This will be carried out by isolating DNA from stool specimens and detecting the presence of E. coli as well as genes associated with antibiotic resistance (i.e. *erm, mef, and mph* genes) via PCR assays.[45] Other possible experiments may eventually include using PCR to detect the presence of toxins or virulence factors (i.e. *eae, stx, bfp-A,VT-1, VT-2*), which are linked to diarrheagenic E. coli strains,[46-47] or exploring the complete microbiome of the stool specimens.

9.3.1 Stool Specimen Collection

Rectal Swab Collection for Culturing/Microbiological Testing

The test will require that the child's parent and examiners work together to obtain a good sample. Is it important to describe the test to the parent so that they can best assist with keeping the child still during the procedure, if necessary.

In place of stool specimens, rectal swabs can be collected in the following way:

1. Put on a clean pair of gloves.
2. Partially open the fecal swab package and remove the top section of the collection vial (this can be discarded).
3. Position the child:
    - Lie the child on his/her back, hold legs in the air (it is useful to have assistance).
    - Or have the child lay on his/her stomach across the mother/guardian's lap
4. Remove the swab from the package. Take care that the cotton tip is not touched. If it is touched, throw the swab away and begin with a new one.

5. Insert the tip of the swab into the child's anus only as far as needed to contact fecal material (1-3cm) and rotate 180 degrees. The tip should be a brownish color when removed.
6. Place swab into the preservative in the collection tube. Make sure the swab is fully submerged in the liquid preservative and then break the swab off using the pre-scored breaking point.
7. Screw the cap back on the tube and make sure that it's tightened. Wrap the area where the cap meets the tube with Parafilm to ensure that the sample will not leak, and then place the tube into the appropriate sample box.
    - If the swab cannot be broken off while the tip is fully submerged in the liquid, try twirling the swab in the liquid first (to release the contents of the sample into the preservative) before breaking it off. Avoid rubbing the sample on the tip of the swab off on the side of the tube where there is no liquid.
8. Place a random number label on the collection tube.
9. Place the tube the rectal swab container.
10. **Swab storage for Genetic analysis:** Store samples at room temperature. According to the manufacturer, the preservative in the tube will preserve DNA for 5 months at room temperature (7 days for RNA), and thereafter can be frozen (-20°C or -80°C) for long-term storage.

Stool Specimen Collection for Nucleic Acid Based Testing

For the study participant/parent of the child:
1. Collect the initial stool specimen on a piece of plastic.
2. Transfer a few heaping spoonfuls of the fresh stool into the smaller, 4 oz disposable plastic container that has a locking lid, using the spoon provided. Return this to the trained field worker.

For the trained field worker:
1. Wearing fresh gloves, carefully place a portion of the stool sample from the disposable 4 oz plastic container into a labeled Norgen Stool Nucleic Acid Collection and Transport Tube (15 ml collection tube that contains preservative), using the small spatula that is attached to the tube's cap. Fill up to the line as indicated by the tube.[44] Make sure to spoon the stool into the tube without touching the rim or outside of the tube to avoid any contamination.
2. Once the stool sample has been added, place the cap tightly back onto the tube.
3. Mix gently until the stool is well submerged under the preservative. Do not shake the tube up and down, just gently swirl.
4. Wrap the lid of the tube with a piece of Parafilm to seal it.

5. Place the tube into the storage box and store at room temperature.
6. Once the final stool sample has been collected in the Norgen Stool Nucleic Acid Collection and Transport Tube, wrap up the initial stool sample in the large receptacle container using the plastic lining and properly dispose of it. Also, dispose of the stool sample in the 4 oz plastic container and the spoon that was used.

9.3.2 Materials for Stool Specimen Collection

Rectal Swab Collection for Culturing/Microbiological Testing
Swab
An individually-wrapped Copan flocked swab with a plastic shaft will be used to collect the rectal swab and then placed into a Stool Nucleic Acid Collection and Transport Tube containing Norgen Stool Preservative or Amies Transport Medium.

Sample Tube with Media
The specimen will be in a sterile Stool Nucleic Acid Collection and Transport Tube containing Norgen Stool Preservative or Amies Transport Medium with a cap that will be tightened firmly.

Stool Specimen Collection for Nucleic Acid Based Testing
Plastic Lining
Each participant will be given a piece of plastic that will be placed on the ground to collect the initial stool sample.

Spoon
Wooden medical spoon used to transfer a portion of the initial stool specimen to the small plastic container, which will be brought to the trained field worker by the parent.

Small Plastic Container
4 oz disposable plastic container with locking lid used to transport a portion of the initial stool specimen to the trained field worker.

9.4 Quality Control Measures for Specimen Collection

Negative Field Controls
Negative field control swabs for NP and stool will be taken in each community to assess for contamination: one control swab each (NP and stool/rectal) are taken

before specimen collection begins in a community; and another (NP and stool/rectal) upon completion of specimen collection.

1. For each negative field control, the examiner will open a new swab as described above.
2. Wave the swab in the air, without making contact with anyone/anything.
3. Tube the swab in media, as described above.

Duplicate swabs
Duplicate NP swabs and rectal swabs/stool specimen will be collected from two children per community.

Chapter 10: Cost-Effectiveness

10.1. Costs
We will record the costs of distributions in a random subset of 12 villages at MORDOR 6 ***or*** 12 and MORDOR 24. We will assume a societal perspective. Programmatic costs will be included, such as: azithromycin, antibiotic distributors, supervisors, drivers, fuel, and the time of community volunteers, and the costs of azithromycin will be estimated using the market price for generic formulations. See appendix for the study forms to be used. Collection of certain costs will be incorporated into the electronic data capture system, including the duration of time that the antibiotic distributors worked, the number of antibiotic distributors per team, the number of doses of azithromycin/placebo given, and the number of doses of azithromycin/placebo wasted. Other costs will be collected on paper forms from the appropriate parties: the drivers will complete paper forms describing costs of fuel and automobile maintenance, and the study coordinator will complete paper forms describing the costs of consumables purchased for the study, as well as the overhead costs of maintaining the mass drug administration office.

10.2 Effectiveness
To estimate effectiveness, we will estimate the childhood mortality rate differently from the primary outcome of the study, in that we will account for all person-time in the community for children aged 1-60 months. Children who are born or move into the study area after the initial census will have their person-time counted in the mortality outcome, regardless of whether they are enumerated at the previous census. Similarly, we will count all childhood deaths in the study area, even if the child never appears on the census. The effectiveness outcome will therefore include children who are born and die within an inter-census interval. Estimating the effectiveness will require several aspects from the census data collection:

- **Pregnancies.** At each census, we will ask whether any household members are pregnant, and at the follow-up census, inquire about the status of the fetus, recording the date of births and deaths.
- **Dates of Births, Deaths, and Migrations.** These dates will be obtained in 2 ways. First, all households will be asked whether any children were born since the previous census, including any infants who already have died, and dates will be recorded. Census workers will also document the date that new households moved into the community, and will ask whether any children were born or have died since making the move. If a child is discovered to be deceased but did not appear on any previous census, the census worker will ask the next of kin about the birthdate, and for how long the child lived in the community being censused. Second, the key informant system will record dates of all births, deaths, and migrations among children aged 1-60 months, and will report these data to the verbal autopsy interviewer. The verbal autopsy interviewer will enter these data into a separate section of the electronic data capture system.

Chapter 11: Training

11.1 Standardization

The three collaborating institutions – UCSF, JHU, and LSHTM – will work together prior to MORDOR 0 to standardize all study procedures. We will review the format, general logistics, and procedures for the house-to-house census. The importance of capturing the vital status of every individual in the study area, including individuals not on the previous census (i.e., new births, deaths and migrations) will be stressed. The importance of capturing those individuals who were born and died in the time period between two censuses will also be highlighted. Census workers who have successfully completed the training will be certified, although certification can be revoked on subsequent quality control checks. Ongoing training activities (before each biannual census) should consist of didactic classroom instruction and mock census activities, followed by in-field training, reviewing the use of the electronic data capture, including charging devices and troubleshooting technical problems.

11.2 Pilot Studies

Prior to MORDOR 0, non-study communities at each study site will be randomly selected as “pilot” villages from the same pool as the mortality and morbidity communities. The research team will conduct all of the study procedures as described in the mortality study and morbidity study (census, sample collection, and treatment) in these pilot villages for training purposes. Similar to the morbidity and resistance communities, samples will be collected from 40 children, aged 1 – 60 months and 40 children, aged 7 – 12 years. For morbidity, blood samples (thick smears, microcuvettes, dried blood spots), conjunctival

swabs, and anthropometric measurements (height, weight, MUAC) will be collected. For resistance, nasopharyngeal, nares swabs, and stool specimens will be collected. Consent will be obtained from the village chiefs and the parents/ guardians of each child prior to sample collection. Everyone in pilot communities will receive one dose of oral azithromycin after sample collection. If the results of the trial are positive, everyone in these villages will also be treated with mass oral azithromycin at the end of the study.

Chapter 12: Sample Organization, Transport, and Storage

12.1 De-identification

All specimens will be labeled in the field with a random identification number linked to the census in the electronic data capture system, but to facilitate masking, only the DCC will have access to the key linking the ID with census information. Age, gender, and community of residence will be available for each specimen, but names will be kept confidential. Therefore, all specimens will be de-identified.

12.2 Specimen Transport

After sample collection, samples from the field will be transported to a local health facility or research center at each study site.

During any international specimen transport, the temperature of the shipper boxes will be documented by a temperature recording device.

Blood Samples

Blood smears (thin and thick) will be transported at room temperature to a local laboratory (depending on capacity at each study site). FTA Elute cards will be transported at room temperature and stored at the health clinic before being transported for processing. TropBio cards will be transferred on ice to the health center and stored at -20°C prior to shipment.

Swabs

Swabs in STGG media will be initially stored in the field at -4°C using a closed, insulated container until arrival at a securely locked freezer at -20°C. Swabs in DNA/RNA Shield media will be stored in ambient temperature in the field and then transferred to a refrigerator or freezer.

Clinic-based Swabs (non-core)

NP swabs in STGG media will be stored in the -20°C freezer at the health clinic, and then transported for processing. NP swabs in DNA/RNA

Shield media will be stored in ambient temperature at the health clinic, then transported for processing.

Stool Samples

Rectal Swabs for Culturing/Microbiological Testing

Rectal swabs preserved in Amies transport medium should be refrigerated until processed. If specimens will be kept more than 2 to 3 days before being cultured, it is preferable to freeze them immediately at -70°C. It may be possible to recover pathogens from refrigerated specimens up to 7 days after collection; however, the yield decreases after the first 1 or 2 days. Frozen specimens should be transported on dry ice.

Stool Samples for Nucleic Acid Based Testing

Specimens preserved in Norgen Stool Nucleic Acid Collection and Transport Tubes can be left at room temperature for 7 days (if preserving RNA) or up to 5 months (if preserving DNA). Specimens can also be transported in the preservative at room temperature. If the samples will be kept for long term storage, they can be placed in a -20°C or -80°C freezer.

12.3 Specimen Storage

Sample storage will occur in two stages: short-term and long-term

12.3.1 Short-term Sample Storage

Samples will be labeled with study ID only and are unidentifiable without access to the study database. Lab capacity and sample export regulations vary between study sites. If there is capacity to perform sample processing in a quality manner (as dictated by IRBs in Tanzania and Malawi), samples will be processed in-country. Otherwise, samples will be shipped to UCSF, JHU, or LSHTM to process core samples and any secondary processing.

12.3.2 Long-term Sample Storage

Depending on each study site's sample export regulations, allowable de-identified samples from Malawi, Niger, and Tanzania will be shipped to UCSF for longer-term storage at the UCSF Oyster Point Facility, which is designed particularly for secure long-term (5 years) storage of biological specimens, at -80°C for future analyses by investigators and other interested parties.

12.4 Catalog Specimens

We will create a list of study data and specimens, including the age, gender, village identification number, treatment assignment, whether treatment was received, vaccination record, and symptom questionnaire. We will also list the date of collection and transport, and the storage conditions while in the field and

while banked at UCSF. This will facilitate identification of specimens for future analyses.

Chapter 13: Microbiology and Molecular Laboratory Procedures

Participant names will not be included in laboratory records and samples will be de-identified. Therefore, laboratory personnel will be masked, preventing identification of the individuals infected. Lab results will not be available for weeks, if not months. Thus, all individuals will be treated according to their study arm, whether or not their lab tests reveal evidence of infection.

13.1 Storage and Transport Media

13.1.1 Preparation of STGG Transport Medium

Intended Use

STGG was originally used for long term storage of bacteria at -70C. This media has also been used to successfully transport and recover bacteria from specimens collected on swabs.

Formula

	Ingredients per number of 1 ml vials							
Ingredients	1	50	100	200	300	400	500	1000
Skim milk powder (Difco, Detroit, Mich.) Cat.232100 500g	.02g	1g	2.0g	4g	6g	8g	10g	20
Tryptone soy broth (Oxoid-CM129 500g) Fisher cmo129	.03g	1.5g	3.0g	6g	9g	12g	15g	30g
Glucose (Difco-Dextrose cat 215530, 500g)	.005g	.25g	.5g	1g	1.5g	2g	2.5g	5g
Glycerol (Difco- cat 228220 500g)	.1ml	5	10ml	20ml	30ml	40ml	50ml	100ml
Distilled water	1ml	50ml	100ml	200ml	300ml	400ml	500ml	1000ml

Procedure

1. Combine four ingredients in flask and swirl to dissolve (about ½ hour). Use glass cylinder to measure Glycerol. Dispense solution in 1.0ml amounts into screw-cap 1.5ml vials (Sarstedt Microtube, Newton, N.C.) Use Sarstedt rack for easy dispensing.

2. Autoclave vials with caps screwed on loosely at 15 lb/in2 and 121 C for 10 minutes.
3. Do not autoclave vials in plastic storage boxes (boxes will not close when cooled). Use old bottom from broken boxes.
4. STGG should appear milky white to light tan in color with a precipitate at the bottom.

Storage and Shelf Life
Cool vials after autoclaving, screw caps tightly and store at -20 or 4C until use. Use vials within 6 months of preparation.

13.1.2 Preparation of Zymo DNA/RNA Shield™

Manufacturer's Product Description
DNA/RNA Shield™ ensures nucleic acid stability during sample storage/transport at ambient temperatures, preserving the genetic integrity and expression profiles of samples. There is no need for refrigeration or specialized equipment. DNA/RNA Shield™ effectively lyses cells and inactivates nucleases and infectious agents (virus), and it is compatible with various collection and storage devices (vacuum tubes, swabs, nasal, buccal, fecal, etc.).

Materials Required

Product	Supplier	Catalog No.
DNA/RNA Shield™<br>1X solution	Zymo Research Corp.	R1100-50 (50 ml)<br>R1100-250 (250 ml)
2ml Loop Screw Cap Micro Tube, PCR Performance Tested<br>(DNA-, DNase-, RNase-, & PCR inhibitor-free)	Sarstedt Inc.	72.694.416
Insert for Loop Screw Cap, Violet	Sarstedt Inc.	65.713.007

Procedure
1. Prepare tubes by adding violet cap inserts to the top of each tube (these are for color coding purposes to help distinguish the DNA/RNA Shield solution from other media being used in the field).
2. Before aliquoting the DNA/RNA Shield, gently mix the solution by either swirling it or inverting the bottle a few times (trying not to create too many bubbles) to ensure that the solution is homogenous. In a clean biosafety cabinet, use sterile technique to aliquot 1 ml of the solution into each 2 ml tube.
3. When you have finished aliquoting, make sure that the cap on each tube has been tightened to prevent tubes from leaking during transport.

Storage and Shelf Life

Store prepared tubes at room temperature (20°C-25°C) until use; do not freeze solution prior to collecting samples. Zymo guarantees the shelf-life of DNA/RNA Shield ™ up to 1 year from the date of receipt. Therefore, if the solution is aliquoted right away, then prepared tubes should be good for 1 year. The shelf-life may be longer, depending on storage conditions.

13.1.3 Preparation of Amies Transport Medium

Rectal swabs will be collected and transported in this medium. It is best prepared from ready to use dehydrated powder, available from most suppliers of culture media. It is usually used at a concentration of 2 grams in every 100 ml distilled water. Fisher Scientific has a dry powder that's available and can be used to prepare the media in house.

Procedure

1. Prepare as instructed by the manufacturer. Dispense the well-mixed medium into screw-cap tubes. Sterilize by autoclaving (with caps loosened) at 121°C for 15 minutes.
2. When the medium has cooled, tighten the caps. During cooling, invert the tubes to ensure an even distribution of the charcoal.

Storage and Shelf Life

Prepared Amies medium in tightly screw-capped tubes can be stored in a cool place at room temperature (away from direct light) until use. Refer to the manufacturer's instructions/guidelines for specifics on expiration dates.

13.1.4 Preparation of Nutrient Agar Medium/Slants

For isolate storage and shipping, blood agar base (BAB), tryptone soy agar (TSA), heart infusion agar (HIA) and nutrient agar are examples of good storage media for enteric organisms. Nutrient agar slants will be used to store/ship isolates originating from samples such as the rectal swabs. Nutrient agar medium is best prepared from ready to use dehydrated powder, available from most suppliers of culture media (i.e. Fisher Scientific). In general, nutrient agar is used at a concentration of 2.8 g in every 100 ml distilled water.

Procedure

1. Prepare as instructed by the manufacturer. Dispense the well-mixed, pre-melted agar medium into tubes (anywhere from 3-5 ml, depending on the size of the tube). Sterilize by autoclaving the tubes (with caps loosened) at 121°C for 15 minutes.

2. While the tubes are still hot after autoclaving, place them in a slanted position to provide a short slant and deep butt (2 to 3 cm). Once medium is cooled and agar is set, caps can be tightened and slants should be ready for use.

*To inoculate, use an inoculating loop to streak the isolate onto the surface of the agar slant. Incubate overnight at 35° to 37°C.

Storage and Shelf Life
Prepared nutrient agar slants can be stored in a cool dark place and may have a shelf life of up to 2 years provided there is no change in the appearance of the medium to suggest contamination or deterioration. Refer to the manufacturer's instructions/guidelines for specifics on expiration dates.

13.2 Methods for Malaria Testing

13.2.1 Blood Smears
Thin and thick blood smears will be read using standard microbiological techniques at local laboratories at each study site.

13.2.2 Dried Blood Spots

Extracting Genomic DNA from Dried Blood Spots

1. Remove FTA card from plastic bag - there should be 5 dried blood spots. Using the Harris Micro-Punch and mat, punch a 3 mm disc out from one of the blood spots. Place the disc into a 1.5 ml microcentrifuge tube. **When processing multiple samples at a time, make sure to follow the cleaning protocol below in between punching samples.*
2. Add 500 ul of sterile water to the tube and vortex 5 times.
3. Use a clean pipette tip to transfer the washed disc to a clean 1.5 ml microcentrifuge tube.
4. Add 30 ul of sterile water to the tube with the disc and place it in a heating block set to 95°C for 30 min. After incubating the disc, pulse vortex the tube 60 times and then centrifuge. Remove the pelleted disc with a pipet tip and discard.
5. Store eluted DNA at -20°C until ready for use. It is best to run PCR as soon as possible after extracting DNA. Use 5 ul of eluted DNA in CytB PCR reactions (50 ul reaction) as outlined below.

Punch Cleaning Protocol

1. In between each sample, swirl the Harris Micro-Punch in a beaker containing a 1:10 bleach solution for a few seconds and then swirl the tip in a beaker containing a 70% ethanol solution. Dry/blot the tip on a clean paper towel and then allow the punch to air dry for a couple of seconds before using. Discard the used paper towel in the biohazard waste.
    a. Blank control - After cleaning the punch, you may want to take a blank punch from a clean FTA Elute card with no sample and use it as a negative control to confirm that there is no cross contamination.
2. Clean the cutting mat with a Mini Hypewipe (bleach wipe) and then wipe down with ethanol solution using a paper towel. Discard bleach wipe and paper towel in the biohazard waste. (You only have to clean the mat every couple of samples, as long as you use different areas of the mat to punch out each sample – for example, you can clean the mat after every 4 samples if the samples are punched out in four different quadrants of the mat).

Amplifying Gene Regions (PCR)

1. Set up first round of PCR with outer primers: Protocol was adapted from Hsiang, et al. & Steenkeste, et al. papers[40,45] and based on guidelines provided for Qiagen TopTaq DNA Polymerase (refer to Qiagen *TopTaq PCR Handbook*).[46]

Gene	Primer	Sequence
**CytB**	Outer Forward	CGGTCGCGTCCGGTAGCGTCTAATGCCTAGACGTATTCCTGATTATCCAG
	Outer Reverse	CGCATCACCTCTGGGCCGCGTGTTTGCTTGGGAGCTGTAATCATAATGTG
	Inner Forward	GAGAATTATGGAGTGGATGGTG
	Inner Reverse	TGGTAATTGACATCCAATCC

TopTaq PCR Buffer (10X)	5 ul
dNTPs (10 mM)	1 ul
Forward Primer (10 uM)	2 ul
Reverse Primer (10 uM)	2 ul
Template DNA	5 ul
10x CoralLoad Dye	5 ul
Taq Polymerase	0.25 ul
Water	29.75 ul
Total Volume:	50 ul

Note: When setting up several PCR reactions, make up a mastermix for each primer set by multiplying each volume by the number of reactions you have (making up 2-3 more reactions than needed). Aliquot out 45 ul of mastermix for each reaction and then add 5 ul of template DNA for each sample.

- Use the following cycling conditions for the Bio-Rad S1000 thermocycler:
  Initial step: 95°C for 5 min
  *Denature: 94°C for 30 sec
  Anneal: 60°C for 90 sec
  Extend: 72°C for 90 sec
  Repeat from * (Denaturing step to extension step) for 40 cycles
  Final step: 72°C for 10 min

2. Set up second round of PCR with inner primers:
   a. Use the same recipe as above, but use the specific set of inner primers instead of the outer primers for each reaction. Also, use 5 ul of amplicon DNA (DNA from first PCR reaction) as your target.
   b. Use the same cycling conditions as above.

Detecting PCR Products
Use gel electrophoresis to detect and verify the size of expected PCR products.

If running a single-comb 2% agarose e-gel (12 samples total, including MW marker):

1. Load each well that you plan to add a sample to with 15 ul of water. Then load 5 ul of each sample that contains your PCR product into the correct wells. Load 20 ul of water into any remaining empty wells.
2. Load 15 ul of water into the well designated for the MW marker, and then add 5 ul of the marker to that well.
3. The single-comb 2% e-gel needs to run for about 30 min.

If running a double-comb 2% agarose e-gel (16 samples total, plus 2 MW markers):

1. Load each well that you plan to add a sample to with 15 ul of water. Then load 5 ul of each sample that contains your PCR product into the correct wells. Load 20 ul of water into any remaining empty wells.

2. Load 5 ul of water into the well designated for the MW marker, and then add 5 ul of the marker to that well.
3. The double-comb 2% e-gel needs to run for about 15 min.

If running a 48 2% agarose e-gel (48 samples, plus 4 MW markers):

1. Load each well that you plan to add a sample to with 10 ul of water. Then load 5 ul of each sample that contains your PCR product into the correct wells. Load 15 ul of water into any remaining empty wells.
2. Load 10 ul of water into the well designated for the MW marker, and then add 5 ul of the marker to that well.
3. The 48 2% e-gel needs to run for about 20 min. on program "EG".

(For e-gel procedures: see technical manual for running the gel on the Mother E-Base – Do not do a pre-run, but remember to set up the program for the E-base and to place the gel in the E-Base before loading the samples in the gel)

Purifying PCR Products of Positive Samples

Use Qiagen QIAquick PCR Purification kit and corresponding protocol, eluting the DNA in 50 ul of water.[47,48]

Procedure

1. **Add 5 volumes of Buffer PB to 1 volume of the PCR sample and mix.** In our case transfer whatever is left of the PCR sample (about 45 ul) to a clean 1.5 ml microcentrifuge tube and add 225 ul of Buffer PB to it.

2. **If pH indicator I has been added to Buffer PB, check that the color of the mixture is yellow.**
If the color of the mixture is orange or violet, add 10 µl of 3 M sodium acetate, pH 5.0, and mix. The color of the mixture will turn to yellow.

3. **Place a QIAquick spin column in a provided 2 ml collection tube.**

4. **To bind DNA, apply the sample to the QIAquick column and centrifuge for 30–60 s.**

5. **Discard flow-through. Place the QIAquick column back into the same tube.**

6. **To wash, add 0.75 ml Buffer PE to the QIAquick column and centrifuge for 30–60 s.**

7. **Discard flow-through and place the QIAquick column back in the same tube. Centrifuge the column for an additional 1 min.** IMPORTANT: Residual ethanol from Buffer PE will not be completely removed unless the flow-through is discarded before this additional centrifugation.

8. **Place QIAquick column in a clean 1.5 ml microcentrifuge tube.**

9. **To elute DNA, add 50 µl water (pH 7.0–8.5) to the center of the QIAquick membrane and centrifuge the column for 1 min. Alternatively, for increased DNA concentration, add 30 µl water to the center of the QIAquick membrane, let the column stand for 1 min, and then centrifuge.** IMPORTANT: Ensure that the water is dispensed directly onto the QIAquick membrane for complete elution of bound DNA. The average eluate volume is 48 µl from 50 µl elution buffer volume, and 28 µl from 30 µl elution buffer. Elution efficiency is dependent on pH. The maximum elution efficiency is achieved between pH 7.0 and 8.5. When using water, make sure that the pH value is within this range, and store DNA at –20°C as DNA may degrade in the absence of a buffering agent.

Speciation via Sequencing
Sequetech will automatically quantify the amount of DNA that we send them, and, based on their readings, they will adjust the DNA concentration and optimize the sequencing reaction. For a PCR product that is about 800 bp, they only use about 4 ng of DNA for each sequencing reaction. All the primers that they use in their sequencing reactions are at a concentration of 1.6 uM and they only need 2 ul per reaction.

Preparing the samples for Sequetech
Note: Send primers and template DNA (your samples) in separate tubes – if you premix them, then they cannot quantify your DNA accurately and cannot optimize the sequencing reaction. Also, make sure that all tubes are labeled clearly and match the names that you enter on the order form.

1. Aliquot 6 - 10 ul of each of the purified PCR product that you want to have sequenced into a 1.5 ml microcentrifuge tube and label it clearly (this should be enough DNA for them to sequence each sample both ways).
2. Make up each *nested* primer (keep the forward and reverse primer in separate tubes) that corresponds to your PCR product at a concentration of 3 uM. To do this take 3 ul of the 100 uM stock solution and add it to 97 ul of TE (this will give you a total volume of 100 ul). Based on the number of sequencing reactions you are ordering, calculate how much of the 3 uM primer solution you need to aliquot and send.
   *They will need approximately 1.07 ul of the 3 uM primer solution for each reaction, so to be on the safe side, **allocate 2 ul of the 3 uM primer solution for each sequencing reaction**.
   Calculation: amount of primer needed = (# of sequencing reactions using primer) x 2
3. Organize all the aliquoted and labeled tubes in a storage box and then place the storage box in a sample bag.
4. Place the sequencing order online through Sequetech's website http://www.sequetech.com/, making sure to list all your template DNAs' names, primers' names, the concentration that your primers are at (3 uM), and the approximate size of your PCR products. Print out the order form once you have finished and place a copy of it in the bag with your samples.
5. Schedule a pick-up directly from your location (make sure that everything is ready and scheduled before 3 pm).
6. You can check results online within 24 hours.

13.3 Methods for Resistance Testing

13.3.1 Isolation & Identification of Organisms Using Microbiological Techniques

Various organisms from nasal, nasopharyngeal, and rectal swabs will be isolated using the media and conditions in Table 2 and identified using the methods summarized in Table 3 and below. Standard microbiological handbooks should be referenced for more detailed information.

Procedure for Masking Samples

The microbiologist and all lab staff will be masked to the community of origin.

Laboratory Results Reporting

All lab results will be kept in computer files as well as in hard-copy form by the Microbiologist.

Table 2. Isolation of Organisms from Source - Primary Plating Media & Incubating Conditions

Source	Media	Incubation	Organisms Isolated
Nasal Swab	Blood and MSA	35°C<br>5% CO_2	*S. aureus* (core)
Nasopharyngeal Swab	Blood and Strep select plate, chocolate, chocolate with bacitracin	35°C<br>5% CO_2	*S. pneumoniae* (core)<br>*H. influenzae* (non-core)<br>*N. meningitidis* (non-core)
Rectal Swab	Blood and MacConkey	35°C	*E. coli* (core)

Table 3. Identification of Organisms

Organism	Stain	Identification Tests
S. aureus (core)	Gram stain	Catalase and Tube Coagulase or latex agglutination reagent
S. pneumoniae (core)	Gram stain	Catalase, Optochin disc and/or Desoxycholate reagent, alpha-hemolytic on blood agar
H. influenzae (non-core)	Gram stain	Satelliting growth on blood agar, ALA or other identification tests
N. meningitidis (non-core)	Gram stain	Sugars utilization or other identification kit
S. pyogenes (core)	Gram stain	Catalase, beta-hemolytic on blood agar, and Bacitracin disc or PYR test
E. coli (core)	Gram stain	Indole, oxidase, beta-hemolytic on blood agar, PYR and other identification tests as required

Description of Identification Tests:
We will follow the standard quality control methods already in place at the laboratory. For example, lab staff will perform positive and negative growth controls for all media, and positive controls for all stains and identification tests at a pre-determined schedule.

Catalase Test:
A few drops of 3% hydrogen peroxide are added to colonies removed from agar and smeared onto a clean glass slide, and immediately observed for the release of oxygen or "bubbling." Bubbling is interpreted as the presence of catalase. Lack of bubbling is a negative test.

Tube Coagulase:
Coagulase plasma is inoculated with an isolated staphylococcal colony and incubated for up to 24 hours at 37°C. Any degree of clot formation is a positive reaction and no clot is a negative reaction.

Latex Agglutination:
A few staphylococcal colonies are mixed with the latex agglutination reagent and observed for agglutination. A positive reaction is agglutination of the latex particles, while no agglutination is a negative reaction.

Optochin Test:
An optochin disc is placed onto a blood agar subcultured plate that has been inoculated with a pure culture of the test organism. Plates are incubated for 24

hours at 35°C in CO_2. If a zone of inhibition of 14 mm or more is formed, then it's *S. pneumoniae*.

Desoxycholate (10%) Test:
A drop of reagent is added to a well-isolated colony on a blood agar plate. Colonies of *S. pneumoniae* will disintegrate after 30 minutes.

Satelliting Growth on Blood Agar:
Colonies resembling Haemophilus are subcultured to a non-selective blood agar plate and cross streaked with Coagulase negative Staphylococcus. Plates are incubated in CO_2 overnight and examined for satellite growth around Staphylococcus.

ALA Test:

Sugars Utilization Tests:

Indole Test:

Oxidase Test:

PYR Test:

13.3.2 Microbiological Resistance Testing for Swabs
Resistance testing will be performed using the Kirby Bauer disc diffusion assay or E-test strips (to determine MIC's).

Quality Control
E. coli ATCC 25922, *S. pneumoniae* ATCC 49619, *H. influenzae* ATCC 49247, *S. aureus* ATCC 25923 and 29213 are used for quality control testing of antibiotic discs and E-test strips.

Kirby Bauer Assay
A suspension of the isolate is prepared and turbidity adjusted until it reaches the 0.5 McFarland standard. The suspension is used to inoculate the appropriate susceptibility agar plate for confluent growth. The plate is allowed to dry. Antimicrobial disks are placed on the agar plates with sterile forceps or tweezers; disks may not be moved once they have touched the plates. After the disks are placed on the plate, the plates are inverted and incubated at 35°C for 16 to 18 hours. After incubation, the diameter of the zones of complete inhibition (including the diameter of the disk) (Figure 2) are measured and recorded in millimeters. The measurements can be made with a ruler or sliding caliper on the

undersurface of the plate without opening the lid. The zones of growth inhibition will be compared with the zone-size interpretative table and recorded as susceptible, intermediate, or resistant to each drug tested according to CLSI guidelines (see Appendix).

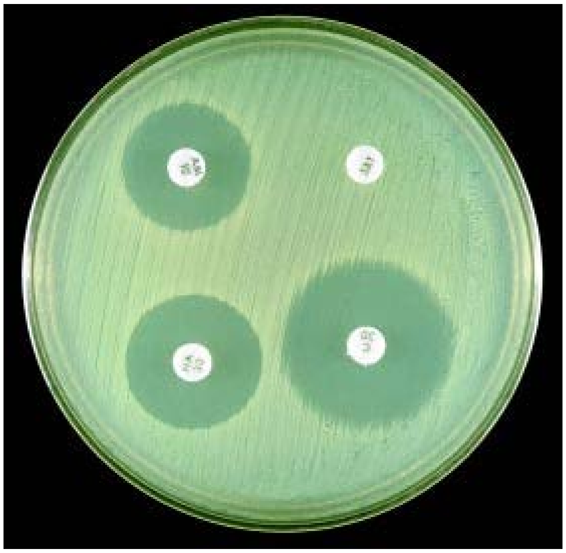

Figure 2. Results of the disk diffusion assay. This *Shigella* isolate is resistant to trimethoprim-sulfamethoxazole and is growing up to the disk (SXT), the zone of which is recorded as 6 mm.

E-test strips.
In addition, azithromycin Minimum Inhibitory Concentrations (MIC) are determined using E-test strips. A suspension of the isolate is prepared and turbidity adjusted until it reaches the 0.5 McFarland standard. The suspension is used to inoculate the appropriate susceptibility agar plate for confluent growth. The plate is allowed to dry. E-test strips are placed on the agar plates with sterile forceps or tweezers; strips may not be moved once they have touched the plates. After the strips are placed on the plate, the plates are inverted and incubated at 35°C for 20 to 24 hours. The MIC is read where the ellipse intersects the MIC scale on the strip. The MIC will be compared with the interpretative table according to CLSI guidelines (see Appendix).

Table 4. Antibiotics Testing Guidelines

Specimen	Organism	Antibiotics Tested
Nasal	*S. aureus*	Azithromycin, cefoxitin
Nasopharyngeal	*S. pneumoniae*<br>*H. influenzae*<br>*N. meningitidis*	Azithromycin, clindamycin, penicillin (Note: *S. pneumoniae* - 1 ug oxacillin used to predict penicillin resistance for Kirby Bauer)
Stool	*E. coli*	Azithromycin, ampicillin, co-trimoxazole

Materials Required for Susceptibility Testing:

- MH w/ 5% sheep blood plates
- MHA plates
- MH broth or sterile 0.85% saline
- Sterile cotton-tipped swabs
- 0.5 MacFarland Standard
- Blood and chocolate plates
- Caliper for reading zones
- Forceps
- Light source
- Vortex
- 35°C ambient air and 35°C CO_2 incubator

13.3.3 Nucleic Acid Based Resistance Testing for Stool Specimens

Stool specimens not cultured will undergo nucleic acid based resistance testing. DNA from the stool samples will be extracted using some type of DNA isolation kit (i.e. Norgen Stool DNA Isolation Kit) and the appropriate corresponding protocol.[49] PCR and gel electrophoresis will then be performed with specific primers to detect genes associated with *E. coli* and antibiotic resistance (specifically macrolide resistance).

Extracting DNA from Stool Procedure

1. **Lysate Preparation**
   a. Mix the sample well to make sure that the stool/preservative mixture is homogenous. Carefully remove the lid from the collection tube and aliquot 200 µl of the stool/preservative liquid and add it to a Bead Tube provided with the kit (it may be easier to use a pipet tip that has been cut to make the opening wider or to use a large orifice pipet tip).
   b. Add **Lysis Solution** to the Bead Tube, so that the final volume is 1 ml (for our purposes, add 800 µl of Lysis Solution). Vortex and centrifuge briefly.
   c. Add 100 µl of **Lysis Additive** and vortex briefly.
   d. Secure the Bead Tube horizontally on a flat-bed vortex pad with tape, or secure the tube in any commercially available bead beater equipment (e.g. Scientific Industries' Disruptor GenieTM). Vortex for 3 minute at maximum speed.
   e. Centrifuge the tube for 2 minutes at **14000 × g (~14,000 RPM)**.
   f. Transfer up to 600 µl of supernatant to a DNAase-free microcentrifuge tube (not provided).
   g. Add 200 µl of **Binding Solution**, mix by inverting the tube a few times, and incubate for 10 minutes on ice.
   h. Spin the lysate for 2 minutes to pellet any cell debris.
   i. Using a pipet, transfer up to 700 µl of supernatant (avoid contacting the pellet with the pipet tip) into a 2 ml DNAase-free microcentrifuge tube (not provided).
   j. Add an equal volume of 70% ethanol (provided by the user) to the lysate collected above (100 µl of ethanol is added to every 100 µl of lysate). Vortex to mix.

2. **Binding to Column**
   a. Assemble a spin column with one of the provided collection tubes.
   b. Apply 600 µL of the clarified lysate with ethanol onto the column and centrifuge for 1 minute at 14000 × g (~14,000 RPM). Discard the flow through and reassemble the spin column with the collection tube. **Note:** Ensure the entire lysate volume has passed through into the collection tube by inspecting the column. If the entire lysate volume has not passed, spin for an additional minute.
   c. Repeat step 2b with the remaining volume of lysate mixture.

3. **Column Wash**
   a. Apply 500 µL of **Wash Solution I** to the column and centrifuge for 1 minute.
   Note: Ensure the entire wash solution has passed through into the collection tube by inspecting the column. If the entire wash volume has not passed, spin for an additional minute.
   b. Discard the flow through and reassemble the spin column with its collection tube.
   c. Apply 500 µL of **Wash Solution II** to the column and centrifuge for 1 minute.
   d. Discard the flow through and reassemble the spin column with its collection tube.
   e. Repeat 3c and 3d.
   f. Spin the column for 2 minutes in order to thoroughly dry the resin. Discard the collection tube.

4. **DNA Elution**
   a. Place the column into a fresh 1.7 mL Elution tube provided with the kit.
   b. Add 50 µL of **Elution Buffer** to the column.
   c. Centrifuge for 2 minutes at 200 x g (~2,000 RPM), followed by a 1 minute spin at 14,000 x g (~14,000 RPM). Note the volume eluted from the column. If the entire volume has not been eluted, spin the column at 14,000 x g (~14,000 RPM) for 1 additional minute.
   d. (Optional): An additional elution may be performed if desired by repeating steps 4b and 4c using 50 µL of Elution Buffer. The total yield can be improved by an additional 20-30% when this second elution is performed.

5. **Storage of DNA**
   The purified genomic DNA can be stored at 2-8°C for a few days. For longer term storage, -20°C is recommended.

Amplifying Gene Regions
We will use DNA extracted from stool specimens and published primers to perform PCR reactions for the genes of interest:[50-52]

Purpose	Genes
Detecting E. coli	*malK* (encodes for *malB* promoter) [53]<br><br>*This gene will detect for both *E. coli* and *Shigella* since the two are closely related and are difficult to discriminate from one another.
Detecting Antibiotic Resistance	*mph (A, B)*<br>*erm (A, B, C)*<br>*mef (A)*<br>*ere (A, B)*<br>*msr (A)*

13.4 Methods for *Chlamydia trachomatis*

13.4.1 Description of Abbott RealTi*m*e Assay
Swabs will be processed with the Abbott RealTi*m*e assay for *C. trachomatis*, using the automated Abbott *m*2000 System. The RealTi*m*e assay targets the cryptic plasmid of *C. trachomatis*. The assay has been shown to be highly sensitive and specific for the diagnosis of sexually transmitted *C. trachomatis*, with sensitivities exceeding that of the nucleic acid amplification test used in most recent trachoma studies (Roche AMPLICOR).[54,55]

13.4.2 Processing of Samples for PCR
Samples are handled as per Abbott RealTi*m*e sample processing protocol, with the following modifications:
1. Samples are boiled for 10 minutes at 100°C. Boiling of samples is an accepted treatment method to remove substances that may be inhibitory to the PCR amplification process
2. Samples are pooled.

13.4.3 Procedure for Masking Samples
In order to mask the location origin, the control status, and the clinical exam grading of the conjunctival samples collected in the field, the Database Manager will assign an identification number different from the random number assigned to each child. The PCR results will be recorded according to this laboratory

identification number, thus masking the lab until all the samples have been processed. The Database Manager will then link the lab ID number to the random sample number to reveal the test results by village.

13.4.4 Procedure for Pooling Samples

We will increase the efficiency of chlamydial testing by combining swabs from the same age stratum and same community into pools of 5 random swabs for processing. An internal control will be run with each pool to rule out the possibility of PCR inhibitors. Any inhibitory pools will be re-tested, and if still inhibitory, the swabs will be individually re-tested. If PCR from any pool is equivocal, then all swabs from the pool will be tested individually. While samples will necessarily be diluted in this process, this is not thought to affect the sensitivity of the test.[56] We will unpool all positive pools in the 0-5 year age stratum and estimate the community prevalence of chlamydial infection as the proportion of positive swabs. We will estimate the community prevalence of infection in the 6-9 and ≥10 year age strata using maximum likelihood estimation, similar to our previous trials: the number of individual swabs with the maximum likelihood of having resulted in the observed pooled results will be chosen as the estimate for that village.[57,58]

In order to pool the conjunctival samples in the lab, the microbiology lab staff will assign a new pool ID number for every sample, and samples will be stored at -80°C freezer until PCR testing (if not processed that day).

13.4.5 Quality Control

1. A *C. trachomatis*(+) control and a *C. trachomatis*(-) control (targeting 136 base pairs of a pumpkin gene) is included in each test run of the Abbott RealTi*m*e assay.
2. To test the effect of sample processing, a known positive sample is processed and tested in each test run. (This control is helpful when testing large numbers of negative samples.)
3. An internal control intended to identify specimens that contain polymerase inhibitor is run routinely on each sample. The internal control helps identify false negative results.

13.4.6 Quantification

For every 0-5 year old who tests positive for chlamydia, we will also run PCR for the beta actin gene on the same sample, in order to normalize the quantity of chlamydial DNA to the amount of the specimen. Quantitative results from the Abbott system are given in terms of a decision cycle (DC) number. We will generate a ratio of the DC number of the chlamydial DNA to the DC number of the beta actin gene and use the resulting ratio as the chlamydial load.

Chapter 14: Study Medication

Children aged 1-60 months on the current census will be offered weight- or height-based, directly observed, oral suspension (azithromycin or placebo) every 6 months for 2 years (as performed in trachoma programs) at each study site. In addition to being at least 1 month of age, children should weigh at least 3.8 kg to be eligible for treatment. This ensures that mistakenly aged or premature infants won't be treated. These infants will be eligible for treatment at the subsequent distribution, approximately 6 months later. The mortality application will not provide a dose for children weighing <3.8 kg.

We will monitor adverse events following mass treatments as described in the adverse events section. The treatment and monitoring schedule for all study arms is shown in Table 1.

14.1 Study Medication Description (from Pfizer, Inc.)

Azithromycin

Zithromax® for oral suspension is supplied in bottles containing azithromycin dehydrate powder equivalent to 1200mg per bottle and the following inactive ingredients: sucrose; tribasic anhydrous sodium phosphate; hydroxypropyl cellulose; xanthan gum; FD&C Red #40; and flavoring including spray dried artificial cherry, crème de vanilla, and banana. After constitution, a 5mL suspension contains 200mg of azithromycin.

14.1 Dosage Information

Azithromycin and placebo will be administered as a single dose, in oral suspension form for children. Dosing will follow the WHO recommendations for treatment of active trachoma:

- Single dose of 20mg/kg in children (up to the maximum adult dose of 1g)
- Height-based dosing of children (this dosing method is supported by the WHO)

Individuals who are allergic to macrolides/azalides will not be treated.

14.2 Medication Procurement/Donation

Azithromycin (Zithromax®) and the placebo have been donated by the Pfizer Corporation. There will be no costs to acquiring the study medication. Pfizer, Inc. will ship azithromycin and placebo directly to the study sites.

Representatives of each study site will manage the customs process and transport the medication from the port to storage sites.

14.3 Medication Quality Control

Study medication will be stored at each country site prior to use. The study coordinator and other local staff will regularly check and record the study medication expiration dates. The expiration dates on the medication containers will be strictly monitored and all expired study medicine will be discarded appropriately.

14.4 Antibiotic Distribution & Monitoring Coverage

After the MORDOR 0 census and monitoring/collection is complete, treatment (azithromycin and placebo) will be administered to all eligible community members per study protocol. Teams will participate in training exercises regarding drug/placebo distribution and recording techniques prior to each treatment cycle. Training will be in accordance with the Zithromax Program Manager's Guide from the International Trachoma Initiative.

During mass drug administration, distribution team members will use tablet computers equipped with an electronic data capture system to seek out each eligible child on the census, administer antibiotic or placebo, and record whether or not each person has been treated. The distribution team will document individual reasons for not being treated (e.g. death, temporary absence, permanent migration, refusal of treatment, etc.). Consumption of medication will be directly observed and the dose distributed will be documented in the electronic data capture system.

We will estimate antibiotic coverage from the most recent biannual census records, aiming for treatment of 80% of children. At the end of each treatment round, the UCSF DCC will identify any children who have missed 2 or more consecutive treatments, and forward this information to the site study coordinator. Census teams will discern the reason for missing treatments (including unrecorded death) at the next scheduled census. This system will serve as a quality control mechanism to reduce the number of false negative deaths in the study.

14.5 Adverse Reactions/Side Effects

Azithromycin is generally well-tolerated. The most common side effects of azithromycin are diarrhea, nausea, abdominal pain, and vomiting, each of which may occur in fewer than one in twenty persons who receive azithromycin. Rarer side effects include abnormal liver function tests, allergic reactions, and nervousness. Diarrhea due to *Clostridium difficile* has been reported in rare cases.

During the consent process, the common adverse reactions that may occur will be explained to parents/guardians and they will be advised to communicate adverse events to local program representatives immediately. If, for any reason, the participant needs further care, they will be referred to the nearest health center for examination and treatment.

The trial sites will be masked to outcomes, so the responsibility for monitoring interim analysis will fall on the DSMC. Statistical monitoring is discussed in the Statistical Analysis Plan. The Data Safety and Monitoring Committee (DSMC) will be given authority to discontinue treatments at any time if there is evidence of unexpected harm.

14.6 Adverse Events Systems

Both active and passive monitoring systems for adverse events are in place for this study, and these monitoring activities will specifically include (but will not be limited to) treated 1-6 month olds. We will monitor adverse events following mass treatments actively at each follow-up census and during a house-to-house survey of all 1-6 month olds in a random selection of azithromycin and control communities.

14.6.1 Passive Adverse Events Monitoring

We will implement a passive monitoring system during the treatment phase, by instructing parents to report any adverse events in the two weeks following each mass azithromycin distribution to a local healthcare provider. Children will be referred for follow up care on a case-by-case basis.

14.6.2 Active Adverse Events Monitoring

During Census

We will employ two forms of active monitoring for adverse events. First, during each follow-up census we will ask the guardians of all 7-12 month-old children in all study communities (both mortality and morbidity), whether their children had an adverse event after the previous mass medication distribution. This will allow assessment of adverse events from medication distribution among the individuals aged 1-6 months at the time of the distribution. This information will be incorporated into the electronic data capture system so that this question is asked for any child aged 7-12 months. Note that this method will be subject to recall bias, so we will also perform a house-to-house survey (described below).

Infant Adverse Events Survey
To identify any adverse events associated with mass treatment, the UCSF DCC will randomly select 30 study communities (15 azithromycin and 15 control communities) to participate in an adverse events survey. This survey will be performed by the census workers masked to treatment arm, approximately 2 weeks after a mass medication distribution. During the survey, adverse events will be elicited only for study participants aged 1-6 months at the previous census. A structured questionnaire will be performed to elicit dangerous side effects, followed by an open-ended question. Specifically, we will ask the primary caregiver about the following symptoms during the time since the previous antibiotic distribution: abdominal pain, vomiting, nausea, diarrhea, dyspepsia, constipation, hemorrhoids or rash. To elicit symptoms of serious infantile hypertrophic pyloric stenosis which has been reported in rare occasions with macrolide use, census workers will be trained to ask about projectile vomiting and feeding problems.

Training
The household survey team will be the same individuals who conducted the census. They will be trained in survey administration methods, including:

1. Obtaining informed consent
2. Accurately selecting the appropriate households to interview
3. Remaining neutral when asking questions (i.e. asking the question exactly as it is written on the paper in a neutral tone of voice, so as not to lead the respondent or introduce bias)

Serious Adverse Events
Any serious adverse events (SAE) will be reported to Pfizer. An **IIR SAE Form** (*Investigator-Initiated Research Serious Adverse Events Form*) will be completed for each event. (See Appendix for form and complete instructions.)

According to Pfizer, an SAE is any adverse event that:

- Results in death
- Is life-threatening (i.e., causes an immediate risk of death)
- Requires inpatient hospitalization or prolongation of existing hospitalization
- Results in persistent or significant disability or incapacity
- Results in a congenital anomaly or birth defect

Or that is considered to be:

- An important medical event

All community residents will be advised to alert a village health worker if they experience, within one week of mass treatment, a serious adverse event (by the preceding definition). An SAE report must be submitted for all deaths in the study – regardless of the time of treatment. The local health worker will report to the on-site study coordinator; who must, within 24 hours, submit a Pfizer **IIR SAE Form** to mordor.sae@gmail.com. TL will review, and forward to Pfizer and/or the Medical Monitor, as appropriate. SAEs must be submitted to Pfizer within 24 hours of receipt from the on-site coordinator. TL will also forward SAE to DSMC if meets criteria of being possibly related to study drug.

14.6.3 Adverse Events Data

We will keep records and report all adverse events of azithromycin to the DSMC. We will report both efficacy and side effects of azithromycin separately for the 1-6 month old age group. For any "sudden deaths" believed to be associated with azithromycin treatment, key informants will immediately notify the verbal autopsy interviewer via SMS message or another appropriate form of rapid communication.

14.8 Supply issues

If a study site runs out of a treatment letter, a request should be sent to mordor.tx@gmail.com to request a replacement for the community in question. This is not blanket permission to substitute one letter for another – if there are several communities for which the assigned treatment has run out, a separate request must be made for each community.

The study site coordinator will make the request; TCP will determine the replacement letter, a member of the DCC (TCP, KR, or ZZ) will make the change(s) in the database.

The field team must log out of the MORDOR mobile app and log in again for the changes to take effect on the front end. The replacement treatment letter will then appear in the app.

Chapter 15: Protection of Human Subjects

Before the study begins, each site will obtain formal ethical approval from their respective national ethics committee. In addition, local staff will approach community leaders to describe the study and answer any questions. Study staff will proceed only if local leadership consents to participate. At the individual level, we will obtain verbal consent from a parent or guardian for all study activities with patient contact, including censuses, examinations, and treatments.

Verbal consent is the best way to obtain consent, due to the high illiteracy rates in each study area. Children will be included in the study only following the receipt of consent from a parent or guardian. If, at any time, a parent or guardian elects to withdraw a family member from the study, they will be free to do so. Individuals who withdraw will be offered the same medical treatment outside the study.

Children with wasting, stunting, malaria, or anemia will be referred for appropriate treatment by trained study personnel, at the nearest health center.

15.1 Institutional Review Board Approval

15.1.1. Niger

UCSF Committee on Human Research
UCSF's Committee on Human Research will annually review study protocol for ethical approval.

Emory University Institutional Review Board
The Emory University Institutional Review Board will annually review study protocol for ethical approval.

Comité d'Ethique du Niger
The study protocol will be reviewed and granted ethical approval by the Comité d'Ethique du Niger before any patient-related research activities begin.

15.1.2 Tanzania

Johns Hopkins University Institutional Review Board
The Johns Hopkins University Institutional Review Board will annually review study protocol for ethical approval.

Tanzania National Institute for Medical Research Ethical Review Board
The study protocol will be reviewed and granted ethical approval by the Tanzanian IRB before any patient-related research activities begin.

15.1.3 Malawi

London School of Hygiene & Tropical Medicine Institutional Review Board
The London School of Hygiene & Tropical Medicine Institutional Review Board will annually review study protocol for ethical approval.

Malawi IRB
The study protocol will be reviewed and granted ethical approval by the University of Malawi Blantyre IRB before any patient-related research activities begin.

15.2 Informed Consent

First, the chairman of each village will be asked for permission to include the village in the study. Additionally, the study will be discussed with all adults in the village by team members who speak the local language(s).

Informed consent scripts will be translated into local languages before the study can begin. Consent scripts will then be back-translated by a different party to ensure comprehension. Consent scripts will be submitted and approved by national IRB committees in Malawi, Niger, and Tanzania prior to study implementation. Then they will be read aloud to each study participant (and his/her parent/guardian) by a local team member who is a native speaker of the local language to ensure that they understand the risks and benefits of participating in all study activities. Young adults and children under 18 years of age, who cannot give consent by law, will be included in the study only following the receipt of verbal informed consent from a parent or guardian. If, at any time, a parent or guardian elects to withdraw themselves or a family member from the study, it will be made clear that they will still be eligible for treatment.

15.3 Risks and Benefits of Study Procedures

15.3.1 Verbal Autopsy

As verbal autopsy requires a family member to answer questions about a deceased loved one, he or she might experience emotional stress and grief related to the death of the child. Interviewers will be trained to address these situations appropriately with awareness of the cultural context before they are allowed to conduct these verbal autopsies. If the family member is in need of a mental health intervention, referrals will be made by the interviewer.

15.3.2 Swabbing Procedures

There are minimal risks to the participant who receives nasopharyngeal, and nares swabbing. Participants may experience some temporary discomfort, but the swabbing involves minimal risk. Any adverse effects, such as nose-bleeds, will be treated immediately by the examiners. Other health care will be provided at no cost to the study participant if necessary to address a study-related adverse health event.

15.3.3 Stool Collection

Stool samples have been collected in this setting before, with essentially no risk to participants.

15.3.4 Blood Testing

Blood testing will include a pin prick to the finger or heel. The major risk of this procedure is infection at the puncture site, though using aseptic technique will minimize this occurrence. Individuals in these communities are familiar with this procedure because all children who present at a health center with fever are offered the pinprick for a malaria thick smear.

15.3.5 Anthropometric Measurements

There are minimal risks associated with the measuring board, scale, or MUAC tapes aside from anxiety during the measurements. Examiners will do their best to ensure that the parent/guardian of the child understands the process of assessing anthropometric measurements. The examiners will attempt to minimize discomfort for all study participants before, during, and after the measurements are taken. Children with wasting, stunting, malaria, or anemia will be referred for appropriate treatment at the nearest health center.

Chapter 16: Study Monitoring

The UCSF team will conduct annual monitoring visits to project sites in Malawi and Tanzania. The LSHTM team will conduct annual visits to Niger. Annual site visits will ensure uniform data collection, as well as adequate training and certification.

Chapter 17: Data and Safety Monitoring Committee Charter

This Charter is for the Data Safety and Monitoring Committee (DSMC) for *Mortality Reduction after Oral Azithromycin* (MORDOR): OPP1032340.[59]

The Charter will define the primary responsibilities of the DSMC, its relationship with other trial components, its membership, and the purpose and timing of its meetings. The Charter will also provide the procedures for ensuring confidentiality and communication, statistical monitoring guidelines to be implemented by the DSMC, and an outline of the content of the Open and Closed Reports that will be provided to the DSMC.

17.1 Primary Responsibilities of the DSMC

The DSMC will be responsible for safeguarding the interests of trial participants, assessing the safety and efficacy of the interventions during the trial, and monitoring the overall conduct of the trial. The DSMC will provide recommendations about stopping or continuing the trial. To contribute to the integrity of the trial, the DSMC may also formulate recommendations relating to the selection/recruitment/retention of participants, to protocol-specified regimens, and the procedures for data management and quality control.

The DSMC will be advisory to the trial leadership group, hereafter referred to as the Steering Committee (SC). The SC will be responsible for promptly reviewing the DSMC recommendations and determining, whether to continue or terminate the trial, and to determine whether amendments to the protocol are required. If needed, the DSMC may seek the advice of a content expert outside of the committee.

17.2 DSMC Membership

The DSMC is an independent multidisciplinary group consisting of epidemiologists, biostatisticians, bioethicists, and clinicians that collectively has experience in the management of infectious diseases and in the conduct and monitoring of randomized clinical trials including subsaharan Africa.

Judd Walson, MD, MPH (Chair): pediatrics and global health
Alan Hightower, MS: biostatistics
Latha Rajan, MD, MPH&TM: global health
Emily Anderson, PhD, MPH: bioethics
Wondu Alemayehu, MD, MPH: global health

17.3 Conflicts of Interest

The DSMC membership has been restricted to individuals free of apparent conflicts of interest. The source of these conflicts may be financial, scientific, or regulatory. Thus, neither study investigators nor individuals employed by the sponsor, nor individuals who might have regulatory responsibilities for the trial products, are members of the DSMC.

The DSMC members will disclose to fellow members any consulting agreements or financial interests they have with the sponsor of the trial, with the contract research organizations (CRO) , or with other sponsors having products that are being evaluated or that are competitive with those in the trial. The DSMC will be responsible for deciding whether these consulting agreements or financial interests materially impact their objectivity.

The DSMC members will be responsible for advising fellow members of any changes in any of the membership requirements that occur during the course of the trial. It may be appropriate for DSMC members who develop significant conflicts of interest resign from the DSMC.

DSMC membership is to be for the full duration of the trial. If any members leave the DSMC, the SC, in consultation with the DSMC, will promptly appoint a replacement.

17.4 Timing and Purpose of the DSMC Meetings

Organizational Meeting

The initial meeting of the DSMC will be an Organizational Meeting. This is during the final stages of protocol development and the purpose is to provide advisory review of scientific and ethical issues relating to study design to discuss

the standard operating procedures and to discuss the format and content of the Open and Closed Reports that will be used to present trial results.

The Organizational Meeting will be attended by all DSMC members, lead trial investigators, and the trial biostatistician. The DSMC will be given the drafts of the trial protocol, the Statistical Analysis Plan, the DSMC Charter, and the current version of the case report forms. At subsequent meetings, committee members will receive Open and Closed Data Reports.

Formal Interim Analysis Meetings
One or more 'Formal Interim Analysis' meetings will be held to review data relating to treatment safety and efficacy, and quality of trial conduct. There will be at least two interim decisions to be made by the DSMC, at approximately 12 months and 24 months into the study.

17.5 Procedures to Ensure Confidentiality and Proper Communication

To enhance the integrity and credibility of the trial, procedures will be implemented to ensure the DSMC has access to all emerging information from the trial regarding comparative results of efficacy and safety, aggregated by treatment arm.

Closed Sessions
Sessions involving only DSMC members and, where appropriate, those unmasked trial investigators (on the Data Coordinating Committee) who generate the Closed Reports (called Closed Sessions) will be held to allow discussion of confidential data from the trial, including information about the relative efficacy and safety of interventions.

At a final Closed Session, the DSMC will develop a consensus on its list of recommendations, including that relating to whether the trial should continue.

Open Session
In order for the DSMC to have access to information provided, by study investigators, or members of regulatory authorities, a joint session between these individuals and DSMC members will be held between the Closed Sessions.

Open and Closed Reports
For each DSMC meeting, Open and Closed Reports will be provided. Open Reports, will include data on recruitment and baseline characteristics, pooled data on eligibility violations, and completeness of follow-up and compliance. The study statistician (TCP) will prepare these Open Reports.

Closed reports, available only to those attending the Closed Sessions of the meeting, will include analyses of primary and secondary efficacy endpoints, including subgroup and adjusted analyses, AEs and symptom severity, , and Open Report analyses that are displayed by intervention group. These Closed Reports will be prepared by the study biostatistician.

The Open and Closed Reports should provide information that is accurate, with follow-up that is complete to within two months of the date of the DSMC meeting. The Reports should be provided to DSMC members approximately three days prior to the date of the meeting.

Minutes of the DSMC Meeting
The research team will prepare minutes for the open portion of the meeting, including the DSMC's recommendations.

Recommendations to the Steering Committee (SC)
At each meeting of the DSMC during the trial, the committee will make a recommendation to the Steering Committee to continue or terminate. This recommendation will be based primarily on safety and efficacy considerations and will be guided by statistical monitoring guidelines defined in this Charter.

Recommendations to amend the protocol or conduct of the study made by the DSMC will be considered and accepted or rejected by the SC. The SC will be responsible for deciding whether to continue or to stop the trial based on the DSMC recommendations.

The DSMC will be notified of all changes to the protocol or to study conduct. The DSMC concurrence will be sought on all substantive recommendations or changes to the protocol or study conduct prior to implementation.

The SC may communicate information in the Open Report to the sponsor and may inform them of the DSMC recommended alterations to study conduct or early trial termination in instances in which the SC has reached a final decision agreeing with the recommendation. The SC will maintain confidentiality of all information it receives other than that contained in the Open Reports until after the trial is completed or until a decision for early termination has been made.

17.6 Statistical Monitoring Guidelines
The SC will propose statistical rules for a futility stopping rule (requested by the sponsor) and an efficacy stopping rule at the first DSMC meeting. A decision will be made whether the efficacy stopping rule is appropriate for the relatively short, 2-year study.

17.7 DSMC Contact Information

Table 5: DSMC Contact Information

Wondu Alemayehu<br>Consultant Ophthalmologist<br>Berhan Health<br>PO Box 6307<br>Addis Ababa<br>Ethiopia<br>+251 910 57 48 42<br>walemayehu@berhan-health.org
Emily Anderson<br>Assistant Professor<br>Loyola University Chicago<br>Health Sciences Division<br>2160 S. First Ave.<br>Maywood, IL 60153<br>708.327.9229<br>emanderson@lumc.edu
Allen Hightower<br>Statistician<br>2189 Oakawana Drive<br>Atlanta, GA 30345<br>706.341.0176<br>awh1953@gmail.com
Latha Rajan<br>Clinical Associate Professor<br>1440 Canal Street<br>Suite 2210<br>Department of Tropical Medicine<br>New Orleans, LA 70112<br>504.988.7970<br>lrajan@tulane.edu
Judd Walson, Chair<br>Associate Professor<br>University of Washington<br>Box 359909<br>325 Ninth Avenue<br>Seattle, WA 98104<br>206.744.3695 (office)<br>206.612.4571 (mobile)<br>walson@uw.edu

Chapter 18: Data Collection, Management, and Security

18.1. Scope of Data

Mortality and morbidity data from Malawi, Niger, and Tanzania will be collected in this trial. Mortality data includes: census, mortality, and treatment. Morbidity and resistance data includes the following: census, mortality, treatment, and morbidity assessments. Costing data will also be collected, including the costs of azithromycin, antibiotic distributors, supervisors, drivers, fuel, and time of community volunteers.

Mortality Study

Trained census workers will collect census data on all households in the study sites (name, birthdate, age, gender of all household members) and keep track of births, deaths, and migration of children eligible for treatment. In addition to biannual census updates, trained community health workers and study supervisors will conduct WHO verbal autopsy interviews through the duration of the study to provide information on the cause of death. Trained distribution teams will collect data on treatment status and dose given to all study participants, if treatment is provided apart from the time of census.

Morbidity Study

Census, mortality, and treatment data collection procedures will be identical to the mortality study. In addition, trained health workers will collect data on core morbidity assessments such as blood samples (thick smears and dried blood spots for malaria, microcuvettes for hemoglobin), stool samples, nasal swabs, and nasopharyngeal swabs. Note that for de-identification purposes a random number sticker will be affixed to each specimen collected. In addition, before sample collection, parents or guardians will be asked a standardized series of questions to determine whether the child has had recent fever, cough, or diarrhea. Clinic-based case finding will be conducted at local health clinics, which will involve transcription of health records.

Certain morbidity assessments will be entered into handheld mobile devices at the time of the examination (e.g. hemoglobin, responses to symptom questionnaire), while lab results for thick smears, dried blood spots, nasal, nasopharyngeal, and stool specimens will be entered after confirmation.

18.2 Data Storage, Management, and Security

Data will be recorded electronically using handheld mobile devices with custom-made software applications and uploaded daily onto a secure, password protected, central server. Rapid transfer of electronically captured data will allow

nearly real-time monitoring of activity at the study site. Each study site will have a local data coordinating center within the study area. All handheld devices and data entry coordinating centers will be password protected, and all changes in data will be noted, including the date of the change, and the person who made the change. To ensure the quality of the data, we will conduct training sessions before each biannual census where needed. The central database application will use hard disk encryption and physical protection of the server (which is to be maintained in a locked room accessible only to authorized personnel). The database will be based on mySQL (which supports standard SQL queries). Data will be backed up off site (providing integrity in case of the physical loss of the server). Data will never be deleted from mobile capture devices until at least one offsite backup has been completed. Data security during electronic transfer will be achieved through use of the Advanced Encryption Standard (AES).

18.3 Data Monitoring and Cleaning

Data monitoring and cleaning will be overseen by the data coordinating center (DCC) at the coordinating site. Data collection will be monitored on a weekly basis by the site study coordinator using the dashboard function on Salesforce. The Salesforce dashboard will consist of the following reports by study site: Date Household Census Completed, Number of Households Census Completed by Village, Percent Household Census Completed by village, Treatment Status by Worker, Age Distribution by Worker, Sex Distribution by Worker, GPS Missing by Worker, GPS Missing by Village, Number of Records Synced by Date, Assigned Treatment by Given Treatment, Treatment Status by Age, Treatment Status by Village, Age Distribution by Village, and Sex Distribution by Village.

The DCC will ensure that the site study coordinators log on to Salesforce weekly to confirm the status of the dashboard. In addition, upon each village census completion, the DCC will create and maintain a STATA program to identify data quality concerns. Any such concerns which must be addressed at the site specific level will be queried by the DCC. At every phase, as each village is completed and the data is considered cleaned, the data will be locked and a list of deaths will be generated and provided to each site for verbal autopsy.

References

1. Rajaratnam JK, Marcus JR, Flaxman AD, et al. Neonatal, postneonatal, childhood, and under-5 mortality for 187 countries, 1970-2010: a systematic analysis of progress towards Millennium Development Goal 4. Lancet 2010;375:1988-2008.

2. Porco TC, Gebre T, Ayele B, et al. Effect of mass distribution of azithromycin for trachoma control on overall mortality in Ethiopian children: a randomized trial. JAMA : the journal of the American Medical Association 2009;302:962-8.
3. Shaikh S, Schulze KJ, Kurpad A, et al. Development of bioelectrical impedance analysis-based equations for estimation of body composition in postpartum rural Bangladeshi women. The British journal of nutrition 2012:1-9.
4. Waage J, Banerji R, Campbell O, et al. The Millennium Development Goals: a cross-sectoral analysis and principles for goal setting after 2015 Lancet and London International Development Centre Commission. Lancet 2010;376:991-1023.
5. You D, Wardlaw T, Salama P, Jones G. Levels and trends in under-5 mortality, 1990-2008. Lancet 2010;375:100-3.
6. Bhutta ZA, Chopra M, Axelson H, et al. Countdown to 2015 decade report (2000-10): taking stock of maternal, newborn, and child survival. Lancet 2010;375:2032-44.
7. Black RE, Cousens S, Johnson HL, et al. Global, regional, and national causes of child mortality in 2008: a systematic analysis. Lancet 2010;375:1969-87.
8. Arifeen SE, Hoque DM, Akter T, et al. Effect of the Integrated Management of Childhood Illness strategy on childhood mortality and nutrition in a rural area in Bangladesh: a cluster randomised trial. Lancet 2009;374:393-403.
9. Sazawal S, Black RE. Effect of pneumonia case management on mortality in neonates, infants, and preschool children: a meta-analysis of community-based trials. Lancet Infect Dis 2003;3:547-56.
10. Moonen B, Cohen JM, Snow RW, et al. Operational strategies to achieve and maintain malaria elimination. Lancet 2010;376:1592-603.
11. Solomon A, Zondervan M, Kuper H, Buchan J, Mabey D, Foster A. Trachoma control: a guide for programme managers. Geneva: World Health Organization; 2006.
12. Melese M, Alemayehu W, Lakew T, et al. Comparison of annual and biannual mass antibiotic administration for elimination of infectious trachoma. JAMA 2008;299:778-84.
13. Lietman T, Porco T, Dawson C, Blower S. Global elimination of trachoma: how frequently should we administer mass chemotherapy? Nat Med 1999;5:572-6.
14. Schachter J, West SK, Mabey D, et al. Azithromycin in control of trachoma. Lancet 1999;354:630-5.
15. Bailey RL, Arullendran P, Whittle HC, Mabey DC. Randomised controlled trial of single-dose azithromycin in treatment of trachoma. Lancet 1993;342:453-6.
16. Coles CL, Seidman JC, Levens J, Mkocha H, Munoz B, West S. Association of mass treatment with azithromycin in trachoma-endemic communities with short-term reduced risk of diarrhea in young children. The American journal of tropical medicine and hygiene 2011;85:691-6.
17. Lode H, Borner K, Koeppe P, Schaberg T. Azithromycin--review of key chemical, pharmacokinetic and microbiological features. The Journal of antimicrobial chemotherapy 1996;37 Suppl C:1-8.
18. Miller GY, Algozin, K. A., McNamara, P. E. and Bush, E. J,. Productivity and economic impacts of feed-grade antibiotic use in pork production. J Agr Appl Econ 2003;12:469-82.

19. Fry AM, Jha HC, Lietman TM, et al. Adverse and beneficial secondary effects of mass treatment with azithromycin to eliminate blindness due to trachoma in Nepal. Clin Infect Dis 2002;35:395-402.
20. Sadiq ST, Glasgow KW, Drakeley CJ, et al. Effects of azithromycin on malariometric indices in The Gambia. Lancet 1995;346:881-2.
21. Lopez AD, Mathers CD, Ezzati M, Jamison DT, Murray CJ. Global and regional burden of disease and risk factors, 2001: systematic analysis of population health data. Lancet 2006;367:1747-57.
22. Chidambaram JD, Melese M, Alemayehu W, et al. Mass antibiotic treatment and community protection in trachoma control programs. Clin Infect Dis 2004;39:e95-7.
23. House JI, Ayele B, Porco TC, et al. Assessment of herd protection against trachoma due to repeated mass antibiotic distributions: a cluster-randomised trial. Lancet 2009;373:1111-8.
24. Goossens H, Ferech M, Vander Stichele R, Elseviers M. Outpatient antibiotic use in Europe and association with resistance: a cross-national database study. Lancet 2005;365:579-87.
25. Pihlajamaki M, Kotilainen P, Kaurila T, Klaukka T, Palva E, Huovinen P. Macrolide-resistant Streptococcus pneumoniae and use of antimicrobial agents. Clin Infect Dis 2001;33:483-8.
26. Bronzwaer SL, Cars O, Buchholz U, et al. A European study on the relationship between antimicrobial use and antimicrobial resistance. Emerg Infect Dis 2002;8:278-82.
27. Hotez PJ, Fenwick A, Savioli L, Molyneux DH. Rescuing the bottom billion through control of neglected tropical diseases. Lancet 2009;373:1570-5.
28. Sommer A, Tarwotjo I, Djunaedi E, et al. Impact of vitamin A supplementation on childhood mortality. A randomised controlled community trial. Lancet 1986;1:1169-73.
29. Rahmathullah L, Underwood BA, Thulasiraj RD, et al. Reduced mortality among children in southern India receiving a small weekly dose of vitamin A. N Engl J Med 1990;323:929-35.
30. Vijayaraghavan K, Radhaiah G, Prakasam BS, Sarma KV, Reddy V. Effect of massive dose vitamin A on morbidity and mortality in Indian children. Lancet 1990;336:1342-5.
31. West KP, Jr., Pokhrel RP, Katz J, et al. Efficacy of vitamin A in reducing preschool child mortality in Nepal. Lancet 1991;338:67-71.
32. Vijayaraghavan K, Radhaiah G, Reddy V. Vitamin A supplementation and childhood mortality. Lancet 1992;340:1358-9.
33. Vitamin A supplementation in northern Ghana: effects on clinic attendances, hospital admissions, and child mortality. Ghana VAST Study Team. Lancet 1993;342:7-12.
34. Daulaire NM, Starbuck ES, Houston RM, Church MS, Stukel TA, Pandey MR. Childhood mortality after a high dose of vitamin A in a high risk population. BMJ 1992;304:207-10.
35. Bryce J, Terreri N, Victora CG, et al. Countdown to 2015: tracking intervention coverage for child survival. Lancet 2006;368:1067-76.

36. Jones G, Steketee RW, Black RE, Bhutta ZA, Morris SS. How many child deaths can we prevent this year? Lancet 2003;362:65-71.
37. International Trachoma Initiative: Frequently Asked Questions. (Accessed November 3rd, 2011, at http://trachoma.org/iti-frequently-asked-questions.)
38. Fenwick A, Molyneux D, Nantulya V. Achieving the Millennium Development Goals. Lancet 2005;365:1029-30.
39. Verbal Autopsy Standards: Ascertaining and attributing causes of death. 2013. (Accessed March 6th, 2013, 2013, at http://www.who.int/healthinfo/statistics/verbalautopsystandards/en/.)
40. Hsiang MS, Lin M, Dokomajilar C, et al. PCR-based pooling of dried blood spots for detection of malaria parasites: optimization and application to a cohort of Ugandan children. Journal of clinical microbiology 2010;48:3539-43.
41. Sutcliffe J, Grebe T, Tait-Kamradt A, Wondrack L. Detection of erythromycin-resistant determinants by PCR. Antimicrobial agents and chemotherapy 1996;40:2562-6.
42. Pai R, Gertz RE, Beall B. Sequential multiplex PCR approach for determining capsular serotypes of Streptococcus pneumoniae isolates. Journal of clinical microbiology 2006;44:124-31.
43. Tobias J, Vutukuru SR. Simple and rapid multiplex PCR for identification of the main human diarrheagenic Escherichia coli. Microbiol Res 2011.
44. Norgen Biotek Corporation. Stool Nucleic Acid Collection and Transport Tubes Product Insert (PI45650-1). In; 2013.
45. Steenkeste N, Incardona S, Chy S, et al. Towards high-throughput molecular detection of Plasmodium: new approaches and molecular markers. Malar J 2009;8:86.
46. Qiagen. TopTaq PCR Handbook 2010.
47. Qiagen. QIAquick Spin Handbook 2008.
48. GE Healthcare. Nucleic Acid Sample Preparation for Downstream Analyses Principles and Methods Handbook.23-9.
49. Norgen Biotek Corporation. Stool DNA Isolation Kit Product Insert (PI27600-3) In; 2009.
50. Phuc Nguyen MC, Woerther PL, Bouvet M, Andremont A, Leclercq R, Canu A. Escherichia coli as reservoir for macrolide resistance genes. Emerg Infect Dis 2009;15:1648-50.
51. Nakhjavani FA, Emaneini M, Hosseini H, et al. Molecular analysis of typical and atypical enteropathogenic Escherichia coli (EPEC) isolated from children with diarrhoea. Journal of medical microbiology 2013;62:191-5.
52. Ramotar K, Waldhart B, Church D, Szumski R, Louie TJ. Direct detection of verotoxin-producing Escherichia coli in stool samples by PCR. Journal of clinical microbiology 1995;33:519-24.
53. Wang RF, Cao WW, Cerniglia CE. PCR detection and quantitation of predominant anaerobic bacteria in human and animal fecal samples. Applied and environmental microbiology 1996;62:1242-7.
54. Moller JK, Pedersen LN, Persson K. Comparison of the Abbott RealTime CT new formulation assay with two other commercial assays for detection of wild-type and new variant strains of Chlamydia trachomatis. Journal of clinical microbiology 2010;48:440-3.

55. Cheng A, Qian Q, Kirby JE. Evaluation of the Abbott RealTime CT/NG assay in comparison to the Roche Cobas Amplicor CT/NG assay. Journal of clinical microbiology 2011;49:1294-300.
56. Morre SA, van Dijk R, Meijer CJ, van den Brule AJ, Kjaer SK, Munk C. Pooling cervical swabs for detection of Chlamydia trachomatis by PCR: sensitivity, dilution, inhibition, and cost-saving aspects. Journal of clinical microbiology 2001;39:2375-6.
57. Diamant J, Benis R, Schachter J, et al. Pooling of Chlamydia laboratory tests to determine the prevalence of ocular Chlamydia trachomatis infection. Ophthalmic Epidemiol 2001;8:109-17.
58. Gebre T, Ayele B, Zerihun M, et al. Comparison of annual versus twice-yearly mass azithromycin treatment for hyperendemic trachoma in Ethiopia: a cluster-randomised trial. Lancet 2012;379:143-51.
59. Ellenberg SS FT, DeMets DL. Data monitoring committees in clinical trials: A practical perspective: John Wiley & Sons, LTD; 2003.

Appendix

Niger
Malawi
Tanzania
SAP
Abbreviated Verbal Autopsy Questionnaire
Infant Adverse Events survey
Mobile App Manual
Pfizer Investigator Initiated Research Serious Adverse Event Report Form and Completion Guide
Resistance Breakpoints Table (to be added)
Costing Forms (to be added)
Data Management Plan (to be added)

MORDOR MOP Change Log

Date	Version	Edit
1 Apr 2015	1.7	Removed ear swabs from morbidity non-core activities
1 Apr 2015	1.7	Removed symptom questionnaire from morbidity non-core activities
1 Apr 2015	1.7	Modification of section 6.1: Census Supervision.<br>**From:** *Once the study coordinator is confident that the entire community has been reached, and that the amount of missing data are acceptable (defined as <10% of children in a community), the study coordinator will "lock" the census data collection for that community, and no changes can made be made to the census until the next census.*<br>**To:** *Once the study coordinator is confident that the entire community has been reached, and that the amount of missing data are acceptable (defined as <10% of children in a community), the study coordinator (or other research team member) will certify the census data collection for that community complete via Salesforce.*
1 Apr 2015	1.7	Added option to use DNA/RNA shield media by Zymo for NP swab collection (Section 9)
1 Apr 2015	1.7	Added option to use Norgen Stool Preservative for rectal swab collection (Section 9)
1 Apr 2015	1.7	Added details for preparing DNA/RNA shield media (section 13.1.2)
1 Apr 2015	1.7	Added 3.8kg minimum weight requirement for treatment (section 14)
1 Apr 2015	1.7	Added: "Training will be in accordance with the Zithromax Program Manager's Guide from the International Trachoma Initiative." (Section 14.4)
1 Apr 2015	1.7	Added: "An SAE report must be submitted for all deaths in the study – regardless of the time of treatment." (Section 14.6.2)
8 Sept 2015	1.7	Costing optional; clarification that dried blood spots are optional
2 June 2016	1.8	Modification of Section 6.3: Verbal Autopsy, Questionnaire Administration.<br>**From:** *Our goal is to perform each verbal autopsy within 2 months of death.*<br>**To:** *Our goal is to perform each verbal autopsy within 2 months of identification of death.*
2 June 2016	1.8	Modification of Section 4.3: Study Schedule table.<br>**From:** Fall, Winter, Spring, and Summer.

		**To:** Month specific dates i.e. Jan 15-July 15 for each phase.
2 June 2016	1.8	Modification of Section 4.3: Study Schedule table.<br>**From:** Malaria Collection occurring between MORDOR 18 and 24<br>**To:** Malaria Collection as part of MORDOR 6
2 June 2016	1.8	Added in Section 4.3: Study Schedule table:<br>Addition of *: +/- 1 month
2 June 2016	1.8	Modification of Section 4.3: Study Schedule table legend narrative.<br>**From:** *…anthropometry from all children aged…*<br>**To:** *…anthropometry from a cohort of children aged…*
2 June 2016	1.8	Added: "TL will also forward SAE to DSMC if meets criteria of being possibly related to study drug." (Section 14.6.2)
2 June 2016	1.8	Added: "paper" to *Supplies*<br>Added: "with pieces of paper in between each sample." to *Note*<br>(Section 8.2: Set-up drying area for TropBio bloodspots)
2 June 2016	1.8	Added Section **18.3 Data Monitoring and Cleaning**

Mortality Reduction after Oral Azithromycin

Statistical Analysis Plan

UCSF Francis I. Proctor Foundation
The Carter Center
Niger Ministry of Health
Johns Hopkins University, Dana Center, and Bloomberg School of Public Health
Tanzania National Institute for Medical Research and Muhumbili Medical Center
London School of Hygiene and Tropical Medicine
University of Malawi, Blantyre College of Medicine
Malawi Ministry of Health

Introduction

This document (Statistical Analysis Plan, SAP) describes the planned analysis and reporting for the clinical trial, **Mortality Reduction after Oral Azithromycin**. It includes specifications for the statistical analyses and tables to be prepared for the interim and final Clinical Study Report. This study is a Phase IV clinical trial to compare methods to reduce childhood mortality using mass administration of azithromycin (Pfizer, CAS 83905-01-5) compared to placebo. The content of this Statistical Analysis Plan meets the requirements stated by the US Food and Drug Administration and conforms to the American Statistical Association's Ethical Guidelines.

The following documents were reviewed in preparation of this Statistical Analysis Plan:

- Mortality Reduction after Oral Azithromycin, Protocol
- Mortality Reduction after Oral Azithromycin, Manual of Operations
- ICH Guidance on Statistical Principles for Clinical Trials

The planned analyses described in this SAP will be included in future manuscripts. Exploratory analyses not necessarily identified in this Statistical Analysis Plan may be performed to support the analysis. Unplanned analyses not delineated in this Statistical Analysis Plan will be documented as such in the final Clinical Study Report and manuscripts.

This document will be reviewed prior to the enrollment of patients. All subsequent changes will be indicated by detailed change log in the Appendix.

Contents

1 Summary **4**

1.1 Mortality Trial 4

1.2 Morbidity Trial 4

2 Statistical Analysis **4**

2.1 Planned Analyses 4

2.1.1 Mortality Trial 4

2.1.2 Morbidity Trials 6

2.2 Interim Monitoring 9

2.2.1 Stopping Rules 9

2.3 General considerations 10

3 Sample Size Planning **11**

3.1 Mortality trial 11

3.1.1 Malawi 11

3.1.2 Niger 12

3.1.3 Tanzania 13

3.2 Morbidity Trial 13

4 Randomization **14**

5 Abbreviations **15**

1 Summary

1.1 Mortality Trial

The trial profile is given in the Manual of Operations, Ch. 3. In the mortality trial, communities are randomized to two arms: **Azithro** and **Control**. The trial is a placebo controlled, double masked, community randomized, multicenter clinical trial.

1.2 Morbidity Trial

The trial profile is given in the Manual of Operations. In the morbidity trials, communities are randomized to two arms: **AzithroPlus**, or **ControlPlus**. At each study site, 15 communities each are randomized to **AzithroPlus**and to **ControlPlus**. The trial is a placebo controlled, double masked, community randomized, multicenter clinical trial. The profile is given in the Manual of Operations, Ch. 3.

2 Statistical Analysis

2.1 Planned Analyses

2.1.1 Mortality Trial

The primary prespecified analysis will use the number of deaths in each community as the outcome variable. For analysis, a death is counted when an individual in the target age range 1–59 months (inclusive)is enumerated on census i ($i = 0, 1, 2, 3$) and is not on census $i + 1$ due to death. Census i takes place at the beginning of the study, and each subsequent census takes place approximately six months later. The outcome variable is derived by summing the deaths in each period.

Primary Analysis.

The primary analysis will be conducted as negative binomial regression. The number of person-years (for persons in the target age range) is derived by summing the person-times

for each period. The community-level predictor consists of treatment arm for the community. Specifically, the treatment variable is a community level variable, not an individual level variable. An additional categorical regressor will be included for the country. The likelihood-ratio will be computed, and the statistical significance assessed by permutation testing. Specifically, we will assess the significance of including treatment arm at a two-sided level of 0.05 (a mortality effect in either direction will be statistically significant). The number of permutations is chosen as 10,000, large enough to minimize Monte Carlo error; consecutive runs using a prespecified seed will be reported (and if the Monte Carlo confidence interval for the P-value includes the value 0.05, a new set of 100,000 runs will be conducted and the value reported). An estimated aggregation parameter over 30 will result in the choice of Poisson regression. Evidence of poor fit (based on deviance and/or score residuals) will result in reporting of additional models, beginning with estimation of country-specific aggregation parameters. (Note that the primary analysis remains the single multi-country negative binomial regression, with permutation-based significance testing.)

Individuals who are in the target age range at the beginning of any study period, but who are no longer in the target age range by the end, are considered to be fully enrolled and contribute the full amount of person-time for that period.

All three sites will be aggregated together for the single primary prespecified analysis.

Model adequacy will always be checked (e.g. overdispersion for count models), and leave-one-out influence plots will be conducted to indentify highly influential single observations.

In the event of an unanticipated rare event (such as a natural disaster or bus accident) in part of an area, sensitivity analysis will be conducted. Analysis omitting this region will be reported, as well as a stratified permutation test in which we compare treatment and control within strata defined by the affected region. While entirely unsuitable for primary analysis, under certain circumstances it may be possible to remove mortality due to the rare event; we would propose reporting this analysis as well.

Secondary Analyses of mortality.

All secondary analyses will be sharply distinguished from the primary prespecified anal-

ysis and will be identified as such.

First, secondary analyses will be conducted using alternative models. We will also report the results of negative binomial regression using a country-treatment interaction, and we will report the results of Poisson regression at the individual level, using each period of time. Age, treatment group, and country will be used as a covariates. In addition, negative binomial regression for each time period will be analyzed, using time, country, and treatment. A similar analysis will be conducted on individual level data. An analysis will also be performed contrasting the first and second years.

Secondary analyses will be conducted for each country, using data from that country only. This analysis will be negative binomial regression with treatment assignment for each village as the covariate.

Also, secondary analyses will also be conducted using alternative outcomes. An on-treatment analysis of mortality will be restricted to those taking the medication (or placebo) (i.e., comparing placebo-takers to medication-takers). A secondary analysis of those censused but too young to treat will be conducted.

Additional variables.

In the mortality trial, Verbal Autopsy data will also be collected. We propose to use clustered polytomous logistic regression to model the unordered categorical cause of death, using (1) village-level treatment assignment for each person, and (2) country as covariates. A random effect will be included for village. The primary hypothesis test will be permutation-based testing of the significance of the treatment variable.

Secondary analyses on-treatment comparing treated individuals in both arms will be conducted, but sharply distinguished from primary prespecified outcomes.

2.1.2 Morbidity Trials

Primary Analyses.

The morbidity analyses are to be conducted on a country-specific basis. Each outcome will be addressed in a separate analysis.

The outcome and analysis plan for all non-core studies must be submitted to the Steering Committee for approval.

Malaria. The prevalence of malaria at the village level, assessed by dried blood spots on a random sample of children, will be compared between the arms of the trial using ANCOVA. Results will be available at baseline and at the end of the trial. The baseline prevalence in each village will be a village-level predictor; individual children are not longitudinally followed, but are randomly selected at each visit. Permutation testing will yield the P-value. Influential observations will be assessed using leave-one-out jackknife analyses.

A similar procedure will be followed for hemoglobin measurements.

Nasopharyngeal swabs. The fraction of isolates of pneumococcus exhibiting drug resistance will be compared between the morbidity study arms using the same template as for the malaria prevalence analysis.

Nasal swabs. The fraction of isolates of *Staphyloccus* exhibiting drug resistance will be compared between the morbidity arms using the same template as for the malaria prevalence analysis.

Oropharyngeal swabs. The fraction of isolates of *Streptococcus pyogenes* exhibiting drug resistance will be compared between the morbidity arms using the same template as for the malaria prevalence analysis.

Stool specimens/Rectal swabs. The fraction of isolates of enterococci exhibiting drug resistance will be compared between the morbidity arms using the same template as for the malaria prevalence analysis.

Conjunctival exam. The fraction of examined individuals exhibiting the trachoma TF sign, and separately the trachoma TI sign, will be compared between the morbidity arms using the same template as for the malaria prevalence analysis.

Conjunctival swab. The fraction of PCR isolates yielding chlamydia will be compared between the morbidity arms using the same template as for the malaria prevalence analysis.

Clinical photography. The fraction of children yielding positively scored photographs for TF and TI will be compared between the morbidity arms using the same template as for

the malaria prevalence analysis.

Symptom questionnaire. The fraction of children exhibiting symptoms will be compared between the morbidity arms using the same template as for the malaria prevalence analysis.

Anthropometry. In the **AzithroPlus** and **ControlPlus**, anthropological measurements are available at baseline, 12, and 24 months, depending on the age of the child. Some children will contribute measurements at all three time points.

Linear mixed effects regression will be used to model the growth curve for height of children at 12 and 24 months, using baseline height and the square of height as covariates, and controlling for time. The arms of the trial are modeled as fixed effect predictors, using village as a random effect. The parameter of interest is the effect of treatment arm on height. Permutation testing will be used to yield the P-value. Models including interactions between baseline covariates and treatment, and between treatment and time, will be explored. Influential observations will be identified using leave-one-out procedures, and robust linear mixed effects regression conducted where appropriate.

More specifically, we propose to follow this procedure. We will include a random effect for both village and individual, and propose to statistically assess the interaction between arm and time.

The same procedure will be used to analyze weight, MUAC, WHZ, WAZ, and HAZ. The measurements of MUAC are conducted to permit referral of malnourished children, and while these data will be tabulated, reported, and analyzed, the MUAC analyses are to be considered incidental.

The analysis will follow these guiding principles: (1) All data from all available participants will be included, regardless of whether the patient contributed data to all time periods, or to only a subset of periods; (2) The analysis must reflect the longitudinal nature of the followup and the clustered nature of the design, (3) Permutation testing will be used to compute the P-value, (4) The model must be fully prespecified prior to data collection.

Additional arm for resistance monitoring. Longitudinal negative binomial regres-

sion will be conducted on counts of drug resistant isolates, using time and time-treatment arm interactions. A random effect will be used for village.

2.2 Interim Monitoring

2.2.1 Stopping Rules

Efficacy. The trial is designed specifically to collect a second year of data, because of interest in whether the treatment effect is durable in a second year. However, in case of a large effect found after one year in the first country to start, it may be desirable to expand treatment to all countries.

A single interim analysis conducted at alpha of 0.001 will be conducted. Specifically, the interim analysis will be conducted when mortality data are available for one third of the patient-time in the study, according to the following:

1. The first country has completed one year of follow-up
2. Each country has contributed at least six months of follow-up for half or more of the sites within each country

Note that the individual countries are not all proceeding in discrete waves six months apart, and it is not the case that six months of data will be produced during the first six months of the trial. Because the interim analysis must be completely prespecified, the exact rule will be announced once the start date for each country is determined.

Assuming there would be six months of data for the other two countries, such an analysis would have over 90% power to detect an effect of 24%.

The final analysis would be conducted at alpha 0.049.

The interim analysis will provide approximately 81% power to rule out treatment effects less than 35%, assuming a true effect of 50%. We have approximately 99% power to exclude an effect of 30% or smaller.

For a single country, an interim analysis at alpha of approximately 5×10^{-10} would provide 80% power to detect an effect size of 35%.

Futility. An interim analysis of futility is planned. Specifically, we propose to conduct negative binomial regression on village-level mortality counts comparing the treatment and control arms (using a common aggregation parameter) using only data from the first one third of information. The analytic template will be the same as for the primary outcome, except that country will not appear as a predictor. This analysis will be conducted using simulation. If the conditional power to detect a 25% effect drops below 20% (for the entire trial, assuming the same assumptions that were used for planning), discontinuation of the trial or other changes to the protocol may be made in consultation with the Data and Safety Monitoring Committee.

Interim analysis will be executed by the trial biostatistician at the central site.

2.3 General considerations

Individual level missing data (due to loss to follow-up or droputs) is expected. If an individual is lost to follow-up or otherwise unavailable during an interval of length D, then the individual is assumed to contribute the amount of person-time $D/2$ to the denominator (midpoint imputation).

Village-level missing data could occur for various reasons, including field error, withdrawal of support from village leadership, and so forth. While this has not happened in previous studies in our group, the analytic plan will be to analyze all available information (including data from any village prior to the time of dropout of that village). Sensitivity analysis will be conducted in which various values are imputed to the missing observations to determine how our conclusions would change.

3 Sample Size Planning

3.1 Mortality trial

We use the following formula (cited from [?]), formula 7.3, page 108.

$$c = 1 + \left(z_{\alpha/2} + z_\beta\right)^2 \frac{(\lambda_0 + \lambda_1)/y + k^2(\lambda_0^2 + \lambda_1^2)}{(\lambda_0 - \lambda_1)^2}$$

where

c is the number of clusters per arm,

y is the number of person-years per cluster,

λ_0 is the rate in the control group,

λ_1 is the rate in the treatment group,

k is the coefficient of variation in both groups,

α is the alpha level,

β and is the type II error rate.

3.1.1 Malawi

- The base rate for mortality is assumed to be 0.0148 per year in the target age range.
- We assume the same coefficient of variation for village-level mortality as for the Niger site, i.e. 0.51.
- We assume that 19% of the population is in the target range of 1–59 months (inclusive)for the Malawi site.
- We assume an average community size of 799 people.
- We assume 10% loss to follow-up per year.

- The effect size is 0.15.

The number of clusters per arm for Malawi is 310, the total number of clusters for Malawi is 620, and the expected total number of children involved is 94122.

3.1.2 Niger

The input assumptions are as follows:

- The base rate for mortality is assumed to be 0.02 per year in the target age range, lower than seen in the PRET study in Niger.
- We assume the coefficient of variation for village-level mortality is 0.51 (derived from PRET Niger data).
- We assume that 16.7% of the population is in the target range of 1–59 months (inclusive)for the Niger site. National statistics suggest a slightly higher fraction (19%), which would yield fewer communities than we are planning for [**?**].
- We assume an average community size of 668 people. The communities are known as *grappes*.
- We assume 10% loss to follow-up per year (source: PRET Niger, Z. Zhou, pers. comm.).
- The effect size is 0.15.

The number of clusters per arm for Niger was projected to be 312, the total number of clusters for Niger was projected to be 624, and the expected total number of children involved was projected to be 69472.

The final randomization list contained 646 communities, 30 of which were randomized to the morbidity study.

3.1.3 Tanzania

The input assumptions are as follows:

- Considerable uncertainty exists in the base rate for mortality, with national estimates substantially lower than estimates for nearby regions within the country. For planning, we chose a rate of 0.016 per year in the target age range, but recognize that if the true mortality rate is lower, we will have lower power.

- We assume a smaller coefficient of variation for village-level mortality than for the Niger site. Specifically, this value is 0.4, as determined by consultation with the Johns Hopkins team.

- We assume that 17% of the population is in the target range of 1–59 months (inclusive)for the Tanzania site.

- We assume an average community size of 600 people. The communities are known as *hamlets*.

- We assume 10% loss to follow-up per year.

- The effect size is 0.15.

The number of clusters per arm for Tanzania was projected to be 307, the total number of clusters for Tanzania was projected to be 614, and the expected total number of children involved was projected to be 62628.

The final randomization list contained 644 communities, of which 30 were randomized to the morbidity study.

3.2 Morbidity Trial

For the Morbidity trial, we computed the sample size based primarily on the malaria parasitemia outcome. We assumed a comparison of the proportion exhibiting malaria parasitemia (binary outcome), assuming 40 children per arm. We assume that in the control

arm, the fraction per village showing parasitemia is 0.25, a difference of 0.134 between the arms, and an ICC of 0.056. We assumed an alpha of 0.0167 and a power of 0.8. We find that 15 clusters per arm will provide the desired power.

4 Randomization

Simple random sampling will be used in each country. Within each country, a full list of eligible communities will be provided, and used to fill a column of a table. A second column will be populated with treatment arm names as follows. First, fifteen rows will be filled with the name **AzithroPlus**, and fifteen with the name **ControlPlus**. Then, one half of the remaining rows (rounding to even) will be filled with the name **Azithro**, and the remaining filled with the name **Control**. This column will be shuffled at random, yielding an assignment for each community.

Note that there is no stratification other than country, and the morbidity communities are randomized from the same pool as the mortality communities.

The randomization will be conducted using R. The function `sample` with option `replace=FALSE` will be used to conduct the random shuffling. Note that the choice of the random number seed completely determines the randomization. To ensure the integrity of the randomization, we will choose the seed as follows. We will use the units (least significant digit) of the daily high Fahrenheit temperature in eleven selected US cities on 15 July 2013 as reported by the National Weather Service. A confidential ordered list of these cities will be provided to the DSMC.

Masking will be strictly enforced. Each study site will provide one individual to whom the randomization list will be provided. Investigators and field teams must remain masked and must make no effort to determine which arm a community has been randomized to within the mortality trial or within the morbidity trial.

5 Abbreviations

ANCOVA Analysis of covariance

DSMC Data and Safety Monitoring Committee

HAZ height for age Z score

MUAC Mid upper arm circumference

SAP Statistical Analysis Plan

TF Trachomatous inflammation—follicular

TI Trachomatous inflammation—intense

WAZ weight for age Z score

WHZ weight for height Z score

Mortality Reduction after Oral Azithromycin

Statistical Analysis Plan

UCSF Francis I. Proctor Foundation

The Carter Center

Niger Ministry of Health

Johns Hopkins University Dana Center and Bloomberg School of Public Health

Tanzania National Institute for Medical Research and Muhumbili Medical Center

London School of Hygiene and Tropical Medicine

University of Malawi, Blantyre College of Medicine

Malawi Ministry of Health

Introduction

This document (Statistical Analysis Plan, SAP) describes the planned analysis and reporting for the clinical trial, **Mortality Reduction after Oral Azithromycin**. It includes specifications for the statistical analyses and tables to be prepared for the interim and final Clinical Study Report. This study is a Phase IV clinical trial to compare methods to reduce childhood mortality using mass administration of azithromycin (Pfizer, CAS 83905-01-5) compared to placebo. The content of this Statistical Analysis Plan meets the requirements stated by the US Food and Drug Administration and conforms to the American Statistical Association's Ethical Guidelines.

The following documents were reviewed in preparation of this Statistical Analysis Plan:

- Mortality Reduction after Oral Azithromycin, Protocol
- Mortality Reduction after Oral Azithromycin, Manual of Operations
- ICH Guidance on Statistical Principles for Clinical Trials

The planned analyses described in this SAP will be included in future manuscripts. Exploratory analyses not necessarily identified in this Statistical Analysis Plan may be performed to support the analysis. Unplanned analyses not delineated in this Statistical Analysis Plan will be documented as such in the final Clinical Study Report and manuscripts.

This document will be reviewed prior to the enrollment of patients. All subsequent changes will be indicated by detailed change log in the Appendix.

1 Summary

1.1 Mortality Trial

The trial profile is given in the Manual of Operations, Ch. 3. In the mortality trial, communities are randomized to two arms: **Azithro** and **Control**. The trial is a placebo controlled, double masked, community randomized, multicenter clinical trial.

1.2 Morbidity Trial

The trial profile is given in the Manual of Operations. In the morbidity trials, communities are randomized to two arms: **AzithroPlus**, or **ControlPlus**. At each study site, 15 communities each are randomized to **AzithroPlus** and to **ControlPlus**. The trial is a placebo controlled, double masked, community randomized, multicenter clinical trial. The profile is given in the Manual of Operations, Ch. 3.

2 Statistical Analysis

2.1 Planned Analyses

2.1.1 Mortality Trial

The primary prespecified analysis will use the number of deaths in each community as the outcome variable. For analysis, a death is counted when an individual in the target age range 1–59 months (inclusive) is on census and is not on the next census due to death. Census *0* takes place at the beginning of the study, and each subsequent census takes place approximately six months later. The outcome variable is derived by summing the deaths in each period.

Primary Analysis (Mortality Study).

The primary analysis will be conducted as negative binomial regression. The number of person-years (for persons in the target age range) is derived by summing the person-times for each period. The community-level predictor consists of treatment arm for the community. Specifically, the treatment variable is a community level variable, not an individual level variable. An additional categorical regressor will be included for the country. The likelihood-ratio will be computed, and the statistical significance assessed by permutation testing. Specifically, we will assess the significance of including treatment arm at a two-sided level of 0.05 (a mortality effect in either direction will be statistically significant). The number of permutations is chosen as 10,000, large enough to minimize Monte Carlo error; consecutive runs using a prespecified seed will be reported (and if the Monte Carlo confidence interval for the P-value includes the value 0.05, a new set of 100,000 runs will be conducted and the value reported). An estimated aggregation parameter over 30 will result in the choice of Poisson regression. Evidence of poor fit (based on deviance and/or score residuals) will result in reporting of additional models, beginning with estimation of country-specific aggregation parameters. (Note that the primary analysis remains the single multi-country negative binomial regression, with permutation-based significance testing.)

Individuals who are in the target age range at the beginning of any study period, but who are no longer in the target age range by the end, are considered to be fully enrolled and contribute the full amount of person-time for that period.

All three sites will be aggregated together for the single primary prespecified analysis. The intent is a single negative binomial regression for all three countries together, as the single outcome of the trial.

Model adequacy will always be checked (e.g. overdispersion for count models), and leave-one-out influence plots will be conducted to indentify highly influential single observations. All analyses in this trial will always account for the clustered (community randomized) design.

In the event of an unanticipated rare event (such as a natural disaster or bus accident) in part of an area, sensitivity analysis will be conducted. Analysis omitting this region will be reported, as well as a stratified permutation test in which we compare treatment and control within strata defined by the affected region. While entirely unsuitable for primary analysis, under certain circumstances it may be possible to remove mortality due to the rare event; we would propose reporting this analysis as well.

Secondary Analyses (Mortality study)

All secondary analyses will be sharply distinguished from the primary prespecified analysis and are as the following:

1. Age, treatment group, and country will be used as a covariates in the primary analysis model.

2. Negative binomial regression for each time period will be analyzed, using time, country, and treatment.
3. A Poisson regression at the individual level comparing treatment groups, using each period of time.
4. Negative binomial regression contrasting the first and second years.
5. Negative binomial regression using a country-treatment interaction.
6. Treatment effect by age will be analyzed using subroups of patients defined by the following age brackets: [1, 6), [6-12), [12-18), [18-24), [24, 60). Age of the child will be the age captured at enrollment.
7. We will compare the cause of death, as determined by Verbal Autopsy, between the two arms, using a 2 x k table, permuting by randomization unit.
8. Analyses will be conducted for each country, using data from that country only. This analysis will be negative binomial regression with treatment assignment for each village as the covariate.
9. An on-treatment analysis of mortality will be restricted to those taking the medication (or placebo) (i.e., comparing placebo-takers to medication-takers).
10. Analysis of those censused but too young to treat will be conducted.
11. Data will be formatted to include the Phase 24 vital status of all persons found alive at any of the Phases 0, 6, and 12 (with no indication of them dying in the interim) instead of ***only considering the vital status of persons alive at phase 18*** census.
12. Subgroup analysis of children eligible for 4 treatments, but receiving only 1, 2, 3, or 4 treatments by arm (1 vs. 1, 2 vs. 2, 3 vs. 3, and 4 vs. 4 treatments).
13. A secondary analysis will be conducted on every child who died and had a treatement (antibiotics or placebo) assessing if:

    a. The time of death differs between arms (within a year), using a test of equality of circular distributions (using the phase angle as the outcome variable), with a supplementary comparison conducted by fitting negative binomial regression based on linear predictor $A_1 \sin(\theta t + \phi)$, where the null hypothesis is that the phase offset ϕ is independent of treatment (similarly, we may compare equality of circular means using a two-sample Edwards test). Note that in this analysis, a test of the equality of the amplitude A_1 will also be reported. We will conduct goodness of fit tests using higher order trigonometric terms.

    b. The time between treatment and death differs between treatment arms will be compared by performing a survival analysis that accounts for country. Note that in order to do a survival analysis, we will include children that survived in the analysis.

c. The times of treatment (in children that died) between the two arms a Wilcoxon rank sum test comparing the time of treatment in the deceased between the two groups using the Wilcoxon rank sum test.

14. Alternative outcomes will be considered including:

    a. Verbal autopsy using permanova to test the significance of the treatment variable. Missing verbal autopsy data will not be imputed.

2.1.2 Morbidity Trials

Primary Analyses (Morbiditiy Study).

The morbidity analyses are to be conducted on a country-specific basis. Each outcome will be addressed in a separate analysis. Outcomes are taken at 0, 12, and 24 months.

The outcome and analysis plan for all non-core studies must be submitted to the Steering Committee for approval.

The outcomes for this study are listed on `www.clinicaltrials.gov`. Analyses may be conducted at the village level (accounting thus for clustering), or, when possible, at the individual level accounting for clustering by using mixed effects regression. The listed primary outcomes (4 February 2015) are:

1. Height over time in children aged 1-60 months (Niger & Malawi)
2. Weight, corrected for height over time in children aged 1-60 months (Niger & Malawi)
3. Fraction of macrolide resistance in nasopharyngeal swabs in children 1-60 months (core)
4. Fraction of macrolide resistance from stool specimens in children 1-60 months (core)
5. Fraction of macrolide resistance by nasal swabs in children 1-60 months (Niger only)
6. Presence of malaria parasites in children 1-60 months (core activity)
7. Fraction of conjunctival swabs yielding ocular chlamydia in children 1-60 months (Niger)

Nonnormality, heteroskedasticity, clustering, and outliers will be assessed appropriately.

Secondary Analyses (Morbiditiy Study).

Common templates will be used. Analysis will use cluster-level variables for malaria parasites, macrolide resistance in NP swabs, macrolide resistance in nasal swabs, macrolide resistance in stool specimens, and ocular chlamydia. Clustered logistic regression using baseline values will be used, with the outcome being the 12 and 24 month observations, and time and treatment as covariates in addition to baseline values. Permutation P-values will be reported.

Model checking will be performed to identify poorly fitting models, using the residuals versus fitted values plot and by finding high leverage points. Significance testing will be conducted using permutation tests.

The analysis will follow these guiding principles: (1) All data from all available participants will be included, regardless of whether the patient contributed data to all time periods, or to only a subset of periods; (2) The analysis must reflect the longitudinal nature of the followup and the clustered nature of the design, (3) Permutation testing will be used to compute the P-value, (4) The model must be fully prespecified prior to any analysis of any kind.

If a village randomized to the morbidity study has fewer than 40 children upon census, an additional morbidity village may be recruited in its place.

All secondary analyses are identified as the following:

1. Repeated measures of outcomes for children present at baseline and possibly followed for 24 months. using 0, 12, and 24-month measurements (or all time points processed).
2. Analyze additional outcomes: WHO z-values (height for age, weight for height, weight for age) and MUAC. P-value will be adjusted for these additional outcomes.
3. Separate outcome using Cross-sectional post-test children 25 months and under using 0.05 alpha. Analysis will be similar to longitudinal (height for age, weight for height, outliers, etc. Note that these children were not eligible for the primary analysis.
4. Community-level varaiables will be considered from CSI records.
5. Per-protocol analysis will be performed by arm, i.e. only including those who took the treatment.
6. Other outcomes by treatment arm at 24 months will include percent wasted and percent stunted. Permutation p-values will be computed here.

Sensitivity analysis including the outliers as well as dissociating the measurements in a repeated measures analysis will be conducted. Appropriate consideration will be analyzed for transfromation, heteroskedacity, and outliers.Additional prespecified secondary analyses are listed on www.clinicaltrials.gov.

2.1.3 Microbiome

2.1.3.1 Sample Types

There will be five types of samples collected for microbiome analysis, rectal, nasal-pharangeal, nasal, conjutiva, and blood.

2.1.3.2 Sequencing and Pooling

Samples will be processed using either RNA-sequencing or 16S sequencing. In some instances, specimens will be pooled in the lab by combining aliquots of equal nucleic acid content by QuBit measurement of randomly selected individual's swabs into one microbiome sample per village. There will be one pooled sample of randomly selected specimens (e.g. 10 specimens) for each of the 30 villages, giving us 30 samples total for sequencing.

2.1.3.3 Time Points

Samples for a pre-morbidity study also know internally as "mini-microbiome" were collected in a separate indivdually randomized trial. The Morbidity study will provide microbiome samples collected at 12-months and 24-months.

2.1.3.4 Primary MicrobiomeAnalysis

Primary Analysis for rectal swabs collected pre-morbidity study, 16S-sequencing

Pre-Morbidity RCT (a.k.a. "mini-microbiome") will analyze 80 1-60 month old kids, randomized to placebo or azithromycin. We will obtain pre-treatment rectal swabs on day 0 and post-treatment rectal swabs on day 5. We will compare diversity between arms at day 5, and between time points in each arm. Specific diversity measures to be examined are Shannon's Index (primary), Simpson's Index.

Primary Analysis for rectal swabs collected at 12-months, RNA-sequencing

Primary Analyses for the 12 month rectal swabs will conducted on pooled samples. We will use PERMANOVA on an **L2-norm** distance on bacterial **reads** to determine if treatment significantly explains the difference between the two groups.

PrimaryAnalysis for conjunctiva swabs, RNA-sequencing

Primary Analyses for the 12 month **conjunctiva swabs-RNA sequencing** will conducted on pooled samples. We will use PERMANOVA on an **L2-norm** distance on bacterial **reads** to determine if treatment significantly explains the difference between the two groups.

2.1.3.5 Secondary MicrobiomeAnalysis

Secondary Analysis for all types and timepoints of microbiome samples will include:

- L1-norm distance PERMANOVA on bacterial **reads**
- For RNA-sequenced data: L1, L2-norm distances on virus, parasites, fungi, etc

- Simpson's and Shannon's estimated gamma diversities (transformed into effective number) will be compared by arm using a permutation test without correcting for baseline (ie post test only)
- Conjunctiva swabs, 16S-sequencing at 12-months will be conducted on individual samples. We will use PERMANOVA on an L1-norm distance on bacterial OTUs to determine if treatment significantly explains the difference between the two groups.

2.1.3.6 Clinic-Based Case Findings

Clinic-based case finding will use consecutive cases from all CSIs in Loga district. Consecutive cases of infectious illnesses among 0-5 year-old children will be assessed from the case log from months 0 to 12. The number of cases of infectious illness per randomization unit will be compared for 1 calendar year between the intervention and control groups in a Poisson regression, adjusting for the number of cases of infectious illness recorded in the calendar year preceding the start of the intervention.

2.1.3.7 Clinic-Based NP swabs

Clinic-based NP swabs. We will collect nasopharyngeal swabs from children residing in morbidity communities who present to the CSI with respiratory complaints within the month of the annual morbidity assessment (i.e., month 0, 12, 24), and process swabs for macrolide resistance. The primary analysis will compare macrolide resistance in the azithromycin versus placebo groups using mixed effects regression as described above. As a secondary analysis, we will compare the prevalence of macrolide resistance in clinic-based swabs vs. community-collected swabs in a mixed effects regression, with a permuted p-value that accounts for the stratified nature of the data.

2.3 General considerations

Individual level missing data (due to loss to follow-up or droputs) is expected. If an individual is lost to follow-up or otherwise unavailable during an interval of length D, then the individual is assumed to contribute the amount of person-time $D/2$ to the denominator (midpoint imputation).

Village-level missing data could occur for various reasons, including field error, withdrawal of support from village leadership, and so forth. While this has not happened in previous studies in our group, the analytic plan will be to analyze all available information (including data from any village prior to the time of dropout of that village). Sensitivity analysis will be conducted in which various values are imputed to the missing observations to determine how our conclusions would change.

3 Sample Size Planning

3.1 Mortality trial

We use the following formula (cited from Cluster Randomised Trials Hayes RJ, Moulton LH (2009) ISBN: 978–1-58488-816-1; 315 pages), formula 7.3, page 108.

$$c=1+\left(z_{\alpha/2}+z_{\beta}\right)^{2}\frac{(\lambda_0+\lambda_1)/y+k^2(\lambda_0^2+\lambda_1^2)}{(\lambda_0-\lambda_1)^2}$$

where

c is the number of clusters per arm,

y is the number of person-years per cluster,

$\boldsymbol{\lambda_0}$ is the rate in the control group,

$\boldsymbol{\lambda_1}$ is the rate in the treatment group,

k is the coefficient of variation in both groups,

α is the alpha level,

β and is the type II error rate.

3.1.1 Malawi

- The base rate for mortality is assumed to be 0.0148 per year in the target age range.
- We assume the same coefficient of variation for village-level mortality as for the Niger site, i.e. 0.51.
- We assume that 19% of the population is in the target range of 1–59 months (inclusive)for the Malawi site.
- We assume an average community size of 799 people.
- We assume 10% loss to follow-up per year.
- The effect size is 0.15.
- The desired power is 80%.

The number of clusters per arm for Malawi is 310, the total number of clusters for Malawi is 620, and the expected total number of children involved is 94122. In practice, we allow that these are estimations and will depend on drug/placebo availability, with the request being for 83,333 doses per country, assuming 3 doses per 30ml bottle of suspension.

Supplementary assessment, June 2016: In practice the Malawi villages effective size is 1464 individuals, with a slightly higher baseline mortality rate of 0.005 per year. The sample size of 306 randomization units in both arms appears to give approximately 41% power for an effect size of 15% for Malawi alone.

3.1.2 Niger

The input assumptions are as follows:

- The base rate for mortality is assumed to be 0.02 per year in the target age range, lower than seen in the PRET study in Niger.
- We assume the coefficient of variation for village-level mortality is 0.51 (derived from PRET Niger data).
- We assume that 16.7% of the population is in the target range of 1–59 months (inclusive)for the Niger site. National statistics suggest a slightly higher fraction (19%), which would yield fewer communities than we are planning for [**Error! Reference source not found.**].
- We assume an average community size of 668 people. The communities are known as *grappes*.
- We assume 10% loss to follow-up per year (source: PRET Niger, Z. Zhou, pers. comm.).
- The effect size is 0.15.
- The desired power is 80%.

The number of clusters per arm for Niger was projected to be 312, the total number of clusters for Niger was projected to be 624, and the expected total number of children involved was projected to be 69472. In practice, we allow that these are estimations and will depend on drug/placebo availability, with the request being for 83,333 doses per country, assuming 3 doses per 30ml bottle of suspension. These may be underestimates because of the short interval and the lack of a rainy season.

The final randomization list contained 646 communities, 30 of which were randomized to the morbidity study.

Supplementary assessment, June 2016: In practice the Niger villages appear to have an average of 688 individuals, with a slightly higher baseline mortality rate of 0.025 per year. The sample size of 622 randomization units in both arms appears to give approximately 84% power for an effect size of 15% for Niger alone.

3.1.3 Tanzania

The input assumptions are as follows:

- Considerable uncertainty exists in the base rate for mortality, with national estimates substantially lower than estimates for nearby regions within the country. For planning, we chose a rate of 0.016 per year in the target age range, but recognize that if the true mortality rate is lower, we will have lower power.
- We assume a smaller coefficient of variation for village-level mortality than for the Niger site. Specifically, this value is 0.4, as determined by consultation with the Johns Hopkins team.
- We assume that 17% of the population is in the target range of 1–59 months (inclusive)for the Tanzania site.
- We assume an average community size of 600 people. The communities are known as *hamlets*.
- We assume 10% loss to follow-up per year.
- The effect size is 0.15.
- The desired power is 80%.

The number of clusters per arm for Tanzania was projected to be 307, the total number of clusters for Tanzania was projected to be 614, and the expected total number of children involved was projected to be 62628. In practice, we allow that these are estimations and will depend on drug/placebo availability, with the request being for 83,333 doses per country, assuming 3 doses per 30ml bottle of suspension.

The final randomization list contained 644 communities, of which 30 were randomized to the morbidity study.

Supplementary assessment, June 2016: In practice the Tanzania randomization units appear to have an effective size of individuals, with a slightly higher baseline mortality rate of 0.006 per year. The sample size of 631 randomization units in both arms appears to give approximately 31% power for an effect size of 15% for Tanzania alone.

Note that the primary analysis is all countries together. We anticipate more than 80% power to detect an effect size of approximately 0.10, or ten per-cent.

Update: 2016. We anticipate approximately 80% power to detect an overall effect of 14%, accounting for changes in recruitment and mortality.

3.2 Morbidity Trial

For the Morbidity trial, we computed the sample size based primarily on the malaria parasitemia outcome. We assumed a comparison of the proportion exhibiting malaria parasitemia (binary outcome), assuming 40 children per arm. We assume that in the control arm, the fraction per village showing parasitemia is 0.25, a difference of 0.134 between the arms, and an ICC of 0.056. We assumed an alpha of 0.0167 and a power of 0.8. We find that 15 clusters per arm will provide the desired power.

4 Randomization

Simple random sampling will be used in each country. Within each country, a full list of eligible communities will be provided, and used to fill a column of a table. A second column will be populated with treatment arm names as follows. First, fifteen rows will be filled with the name **AzithroPlus**, and fifteen with the name **ControlPlus**. Then, one half of the remaining rows (rounding to even) will be filled with the name **Azithro**, and the remaining filled with the name **Control**. This column will be shuffled at random, yielding an assignment for each community.

Note that there is no stratification other than country, and the morbidity communities are randomized from the same pool as the mortality communities.

The randomization will be conducted using R. The function `sample` with option `replace=FALSE` will be used to conduct the random shuffling. Note that the choice of the random number seed completely determines the randomization. To ensure the integrity of the randomization, we will choose the seed as follows. We will use the units (least significant digit) of the daily high Fahrenheit temperature in eleven selected US cities on 15 July 2013 as reported by the National Weather Service. A confidential ordered list of these cities will be provided to the DSMC.

Masking will be strictly enforced. Each study site will provide one individual to whom the randomization list will be provided. Investigators and field teams must remain masked and must make no effort to determine which arm a community has been randomized to within the mortality trial or within the morbidity trial.

5 Abbreviations

ANCOVA Analysis of covariance

DSMC Data and Safety Monitoring Committee

HAZ height for age Z score

MUAC Mid upper arm circumference

SAP Statistical Analysis Plan

TF Trachomatous inflammation—follicular

TI Trachomatous inflammation—intense

WAZ weight for age Z score

WHZ weight for height Z score

6 Revisions History

1. **3 March 2016.** Clarified the planned interim analysis for efficacy.
2. **26 April 2016.** Clarification of timing of verbal autopsy collection.
3. **28 June 2016.** Clarification of original power calculations, plus supplementary assessment now that trial is underway.
4. **1 Feb 2017.** Clarified and added secondary analyses for Mortality (page 5).
5. **1 Feb 2017.** Added secondary analyses for Morbidity (page 6).
6. **1 Feb 2017.** Added statstical information for the microbiome analyses for Morbidity (page 7).
7. **6 April 2017.** Page 5, Section 2.1.1. Added the secondary analysis after the DSMC meeting due to the fact that some persons may not be captured at all censuses, but still be alive or dead at the end of phase24. :

A secondary analysis will be conducted by doing the following. Data will be formatted to include the Phase 24 vital status of all persons found alive at any of the Phases 0, 6, and 12 (with no indication of them dying in the interim) instead of ***only considering the vital status of persons alive at phase 18*** *census*

8. **24 May 2017 Page 7, Section 2.1.3 Microbiome** Added the primary analysis for the 12-month MB samples.

Primary Analyses for the 12 month rectal swabs will conducted on pooled samples. Samples will be pooled in the lab by combining equal aliquots of 10 individual's rectal swabs into one microbiome sample per village. There will be one pooled sample of 10 specimens for each of the 30 villages, giving us 30 samples total. We will test for Alpha diversity of the sample (or Gamma diversity of the village) comparing the two arms using a permutation test and not correcting for baseline (post test only).

9. **5 June 2017 Page 7, Section 2.1.3 Microbiome (revised).**

Primary Analyses for the 12 month rectal swabs will conducted on pooled samples. Samples will be pooled in the lab by combining aliquots of equal nucleic acid content by QuBit measurement of 10 individual's rectal swabs into one microbiome sample per village. There will be one pooled sample of 10 specimens for each of the 30 villages, giving us 30 samples total. We will use PERMANOVA on an L1-norm distance on bacterial OTUs (which have undergone rarefication) to determine if treatment significantly explains the difference between the two groups.

Secondary Analysis will include:

- *L2-norm distance PERMANOVA on bacterial OTUs*
- *virus, parasites, fungi, etc*
- *Simpson's and Shannon's estimated gamma diversities (transformed into effective number) will be compared by arm using a permutation test without correcting for baseline (ie post test only).*

10. 10 June 2017 Page 7, Section 2.1.3 Microbiome (revised).

Analysis for rectal swabs, RNA-sequencing

Primary Analyses for the 12 month rectal swabs will conducted on pooled samples. Samples will be pooled in the lab by combining aliquots of equal nucleic acid content by QuBit measurement of 10 individual's rectal swabs into one microbiome sample per village. There will be one pooled sample of 10 specimens for each of the 30 villages, giving us 30 samples total for the RNA-seq processing. We will use PERMANOVA on an ***L2-norm*** *distance on bacterial* ***reads*** *to determine if treatment significantly explains the difference between the two groups.*

Secondary Analysis will include:

- ***L1-norm*** *distance PERMANOVA on bacterial* ***reads***
- *virus, parasites, fungi, etc*
- *Simpson's and Shannon's estimated gamma diversities (transformed into effective number) will be compared by arm using a permutation test without correcting for baseline (ie post test only).*

Analysis for conjunctiva swabs, RNA-sequencing

Primary Analyses for the 12 month ***conjunctiva swabs-RNA sequencing*** *will conducted on pooled samples. Samples will be pooled in the lab by combining aliquots of equal nucleic acid content by QuBit measurement of 10 individual's* ***conjunctival*** *swabs into one microbiome sample per village. There will be one pooled sample of 10 specimens for each of the 30 villages, giving us 30 samples total for RNA sequencing. We will use PERMANOVA on an L2-norm distance on bacterial* ***reads*** *to determine if treatment significantly explains the difference between the two groups.*

Secondary Analysis will include:

- *L1-norm distance PERMANOVA on bacterial* ***reads***
- *virus, parasites, fungi, etc*

- *Simpson's and Shannon's estimated gamma diversities (transformed into effective number) will be compared by arm using a permutation test without correcting for baseline (ie post test only).*

Analysis for conjunctiva swabs, 16S-sequencing

Primary Analyses for the 12 month ***conjunctiva swabs-16 sequencing*** *will be conducted on individual samples. We will use PERMANOVA on an L1-norm distance on bacterial OTUs to determine if treatment significantly explains the difference between the two groups.*

Secondary Analysis will include:

- *L2-norm distance PERMANOVA on bacterial OTUs*
- *Simpson's and Shannon's estimated gamma diversities (transformed into effective number) will be compared by arm using a permutation test without correcting for baseline (ie post test only).*

11. 29 June 2017 Pages: 4-6 Secondary Analysis for Mortality study were numerated, and clarified. Secondary Analysis for Morbidity study were also numerated, and clarified. Microbiome Section was divided into 4 sections and modified.
