# Supplementary material for: Causes of death after biannual azithromycin treatment: A community-level randomized clinical trial
Source: PLoS One. 2021 Sep 24;16(9):e0250197. doi: 10.1371/journal.pone.0250197 (PMC8462712; doi:10.1371/journal.pone.0250197)
Supplement: S2 File — (DOCX) [file pone.0250197.s004.docx]

Table 1: Mortality all causes by age group and arm

| Age group | Placebo | | | Azithromycin | | | Incidence rate Ratio*  (95% CI) | Reduction in mortality | P-value |
| --- | --- | --- | --- | --- | --- | --- | --- | --- | --- |
|  | # deaths | Person years | Incidence rate per 100 person years | # deaths | Person years | Incidence rate per 100 person years |  |  |  |
| 1-5 months | 32 | 2230.5 | 1.43 | 25 | 2190.1 | 1.14 | 0.80  (0.46 – 1.39) | 20%  (-39% to 54%) | 0.42 |
| 6-11 months | 45 | 3597.1 | 1.25 | 42 | 3599.2 | 1.17 | 0.92  (0.57 – 1.48) | 8%  (-48% to 43%) | 0.73 |
| 12-23 months | 60 | 7926.0 | 0.76 | 59 | 7564.1 | 0.78 | 1.03  (0.72 – 1.48) | -3%  (-48% to 38%) | 0.87 |
| 24 -59 months | 63 | 19888.5 | 0.32 | 64 | 19165.5 | 0.33 | 1.05  (0.74 – 1.50) | -5%  (-50% to 26%) | 0.78 |

From age group specific negative binomial regression models at the community level

Table 2. Mortality due to pneumonia/ diarrhea by age group and arm

| Age group | Placebo | | | Azithromycin | | | Incidence rate Ratio*  (95% CI) | Reduction in mortality | P-value |
| --- | --- | --- | --- | --- | --- | --- | --- | --- | --- |
|  | # deaths | Person years | Incidence rate per 100 person years | # deaths | Person years | Incidence rate per 100 person years |  |  |  |
| 1-5 months | 20 | 2230.5 | 0.90 | 10 | 2190.1 | 0.46 | 0.51  (0.23 – 1.13) | 49%  (-13% to 77%) | 0.10 |
| 6-11 months | 20 | 3597.1 | 0.56 | 17 | 3599.2 | 0.47 | 0.81  (0.40 – 1.67) | 19%  (-67% to 60%) | 0.57 |
| 12-23 months | 23 | 7926.0 | 0.29 | 22 | 7564.1 | 0.29 | 1.00  (0.56 – 1.80) | 0%  (-80% to 44%) | 0.99 |
| 24 -59 months | 20 | 19888.5 | 0.10 | 28 | 19165.5 | 0.15 | 1.45  (0.81 – 2.59) | -45%  (-159% to 19%) | 0.21 |

*From age group specific negative binomial regression models at the community level
